# Structural and Electronic Complexities of a Sulfur‐Bridged Di‐Iron Complex Composed of Mono‐ and Di‐Nitrosyl Units

**DOI:** 10.1002/advs.202513976

**Published:** 2025-10-27

**Authors:** Sarnali Sanfui, Manuel Quiroz, Jialu Li, Yang Ha, Feipeng Yang, Jinghua Guo, Nattamai Bhuvanesh, Brad S. Pierce, Perla B. Balbuena, Paul A. Lindahl, Michael B. Hall, Marcetta Y. Darensbourg

**Affiliations:** ^1^ Department of Chemistry Texas A&M University College Station TX 77843 USA; ^2^ Advanced Light Source Lawrence Berkeley National Laboratory Berkeley CA 94720 USA; ^3^ National Synchrotron Light Source II Brookhaven National Laboratory Upton NY 11973 USA; ^4^ Department of Chemistry & Biochemistry University of Alabama Tuscaloosa AL 35487 USA; ^5^ Department of Chemical Engineering Department of Materials Science and Engineering Texas A&M University College Station TX 77843 USA

**Keywords:** bimetallic complex, DNIC, Enemark–Feltham, iron nitrosyl complex, metallodithiolate

## Abstract

The delocalized, thermodynamically stable cation, [(N_2_S_2_)Fe(NO)•Fe(NO)_2_]^+^, an adduct of mono‐nitrosyl and dinitrosyl iron units, is analyzed to address the unusual stability of the sulfur‐bridged diiron complex in its three overall redox levels, +, 0, and −. X‐ray diffraction and myriad spectroscopic techniques probe products of sequential electron uptake in the corresponding neutral and anionic species. Conundrums include unified blueshifts of the overall 3‐band, ν(NO), pattern with added electrons. One‐electron reduction changes the anti‐ferromagnetically coupled, S = 0, cationic diiron species to the neutral analog, S = ½, with unpaired spin mainly localized on the MNIU, which decreases its ∠Fe–N–O angle by 10 degrees in response to the extra electron density. Subsequent reduction to the anionic species, S = 1, involves a major geometric change at the MNIU, which moves the Fe in {Fe(NO)}^8^ out of the N_2_S_2_ plane. Site‐specific ^15^N labeling of nitrosyl in the MNIU confirms the IR analysis and shows rapid NO exchange between the MNIU/DNIU (mono‐nitrosyl iron unit/dinitrosyl iron unit) pairs during its synthesis at RT. Mössbauer spectroscopy, S K‐edge XAS, and molecular orbital calculations confirm the ability of NO and the versatility of sulfur bridges to buffer and distribute electrons, a key to their major importance in metalloenzymes.

## Graphical abstract

Two NO‐containing iron units, biologically relevant as NO storage/release centers, connected by sulfur, display characteristics arising from multiple quantum levels in their extensively delocalized arrangement. Analysis in three charge levels finds the electron distribution almost substantially spread over all atoms of its composition: 2S, 2Fe, and 3NO.

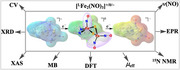

## 1 Introduction

Diatomic ligands such as CO, CN^−^, and NO^+/0/−^ are central to the development of molecular structure and bonding principles related to atomic properties of the metals to which they are bound.^[^ ^1^, ^2^, ^3^ ^]^ Carbon monoxide and cyanide are important natural components in primordial enzyme active sites, e.g. the hydrogenases.^[^ ^4^, ^5^ ^]^ Prominent in biochemistry as well as human physiology, nitric oxide and its ability to traverse three redox states (NO^+^, NO, and NO^−^) are acknowledged as an endogenous signaling agent widely applied to vasodilation, cell death, and immune response.^[^ ^6^, ^7^, ^8^, ^9^, ^10^, ^11^, ^12^, ^13^ ^]^ The physical properties of exogenous NO‐delivery agents are actively researched for the purpose of precise targeting of disease states and control of effective concentrations.^[^ ^14^, ^15^ ^]^

**Figure 1:**
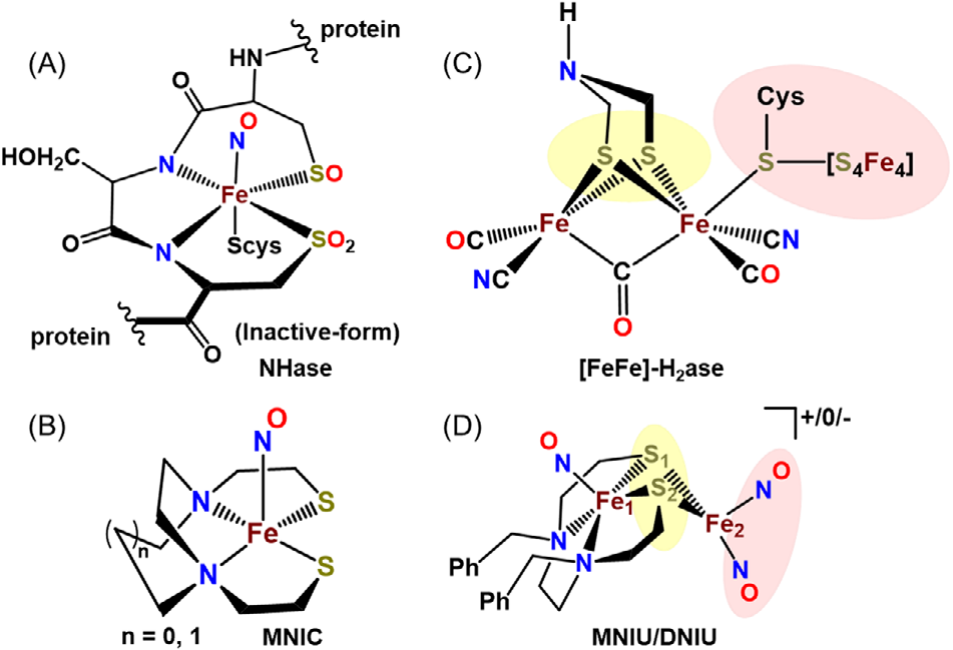
A) Schematic of NO‐deactivated nitrile hydratase (NHase)^[^ ^17^ ^]^; B) Minimal model of deactivated NHase, MNIC (mono nitrosyl iron comlplex); N to N connectors include N,N’‐bis(2‐mercaptoethyl)‐1,5‐diazacycloheptane (**L1**) and N,N’‐bis(2‐mercaptoethyl) diazamethylethane (**L2**);^[^ ^8^ ^]^ C) Schematic of Fe‐Fe hydrogenase ([Fe‐Fe]‐H_2_ase) active site,^[^ ^38^ ^]^ connected to redox active 4Fe4S cluster; D) A Fe_2_(NO)_3_ model of [Fe‐Fe]‐H_2_ase envisions MNIU sulfur bridged to a DNIU, the redox active NO is assumed to model the redox‐active 4Fe4S cluster.

The affinity of nitric oxide for iron is expressed in a broad swath of biological units such as hemoglobin^[^ ^2^, ^16^ ^]^ (Fe‐heme, FeN_4_); nitrile hydratase^[^ ^17^, ^18^ ^]^ (Fe‐NHase, FeN_2_S_2_(O)_3_) or FeN_2_SOSO_2_); Nitric Oxide Reductase^[^ ^16^, ^19^, ^20^ ^]^ (NOR) and flavin nitric oxide reductase^[^ ^21^, ^22^ ^]^ (FNOR as a subclass of NOR). The mono‐iron nitrosyl centered in the N_4_‐heme ligand is regarded as the ultimate resting site responsible for several functions of NO related to vasodilation. The Fe‐NHase active site, in which Cys‐Ser‐Cys sulfurs are partially oxygenated, finds nitrosylated Fe, FeNO, in the inactive form, **Figure** **1**A. A related tripeptide Cys‐Gly‐Cys binding site for one nickel in [Ni‐Ni′]‐Acetyl‐coA synthase may be described as a NiN_2_S_2_ metallodithiolate site attached through thiolate sulfurs to the second, catalytically‐active Ni′.^[^ ^23^, ^24^, ^25^, ^26^, ^27^ ^]^ We have explored the binding of transition metals to biomimetic MN_2_S_2_ complexes as S‐bridge synthons for heterobi‐ and polymetallic complexes in numerous compositions and topological forms; NiN_2_S_2_ has been particularly fruitful in these developments.^[^ ^23^, ^24^, ^25^, ^26^, ^27^, ^28^, ^29^ ^]^ The [Fe(NO)]^2+^ unit is an attractive analog of the NiN_2_S_2_ metallodithiolate ligand, especially for its ν(NO) vibrational marker, its magnetic S = ½ signal, and its electron‐delocalizing potential through the redox‐active {Fe(NO)}^7/8^ unit.^[^ ^25^, ^30^ ^]^ It is shown in Figure 1B as a [Fe(NO)]^2+^ center within an N_2_S_2_ ligand field, a mono‐nitrosyl iron complex, MNIC. Various “receiver” units form bi‐ or polymetallics with S‐bridged structures that are interpretable as donor‐acceptor coordination complexes according to the requirements of the transition metal receiver. These receivers have been Group 10 metal dications, (η^5−^C_5_H_5_)Fe(CO)^+^, [Ni(S_2_C_2_Ph_2_)], and the stability and structural features of the bimetallics have been facilitated by the bridging sulfur from the metallodithiolate.^[^ ^23^, ^26^ ^]^

**Figure 2:**
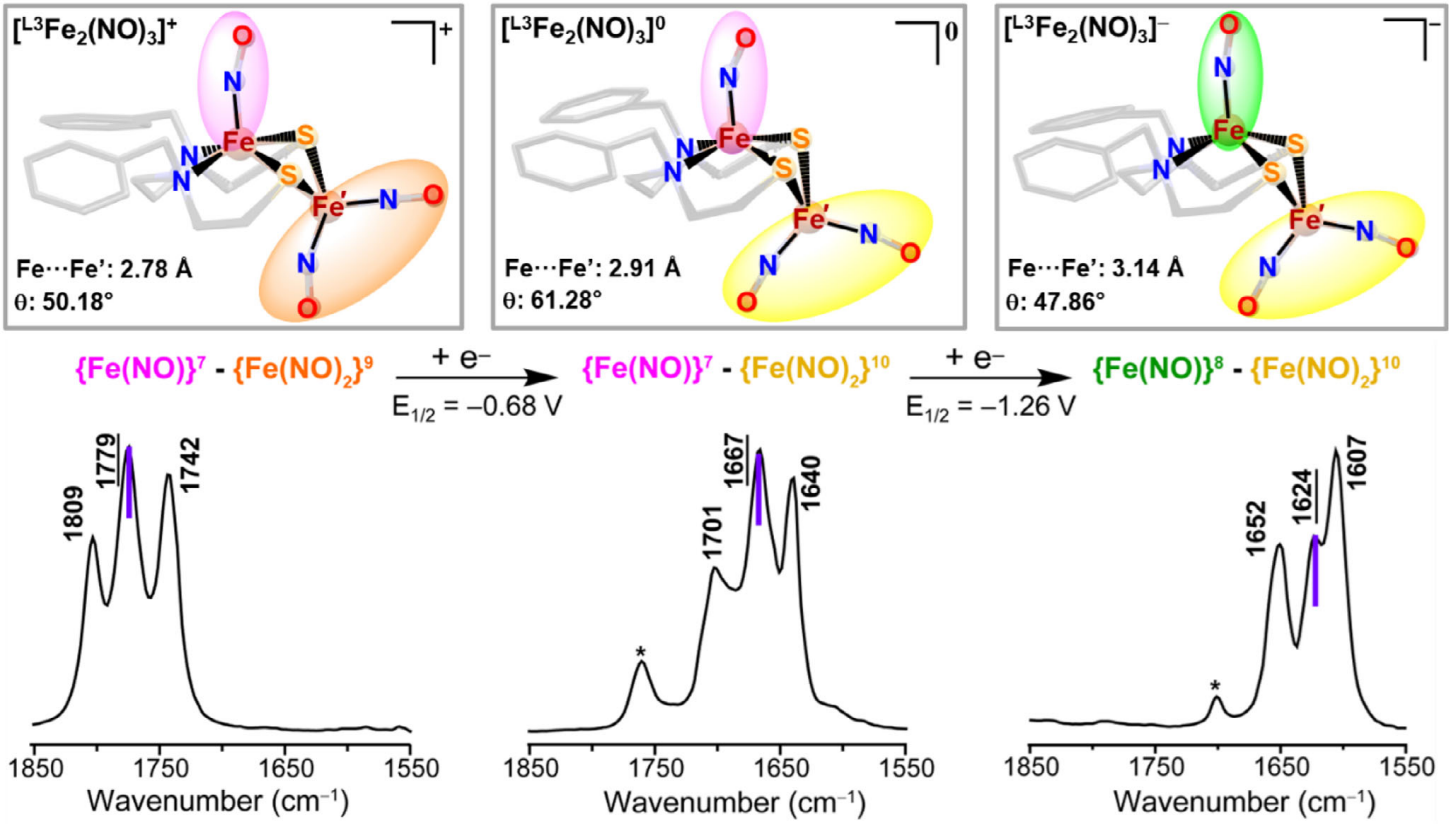
Display of SC‐XRD‐derived molecular structures of **^L3^Fe_2_(NO)_3_** complex in three charge levels; the color code for iron nitrosyls refers to primary positions of electron uptake: {Fe(NO)}^7^ pink, {Fe(NO)}^8^ green; {Fe(NO)_2_}^9^ orange; {Fe(NO)_2_}^10^ yellow). The ν(NO) IR pattern and positions; the verticle purple line in the spectra indicate the stretching frequency of the MNIU. An asterisk ^*^ on the IR spectra is assigned to an undefined impurity.

During the process of fully characterizing the (N_2_S_2_)Fe(NO) complex as a metallodithiolate ligand for several receivers, we encountered an unusually stable and persistent diiron trinitrosyl, (N_2_S_2_)Fe_2_(NO)_3_, cation.^[^ ^31^, ^32^, ^33^ ^]^ This NO‐rich diiron complex contains two iron nitrosyl moieties of biological interest, **Figure** **2**. The mono‐nitrosyl iron side, MNIU (mono nitrosyl iron unit), is a nominal mimic of the inactive form of nitrile hydratase described above. The dinitrosyliron side is the well‐known DNIC (dinitrosyl iron complex), which is usually found within a 4‐coordinate dinitrosyl iron complex whose tetrahedral coordination geometry is completed by myriad donors, including biological ligands. The DNIC is argued to be “a working form of nitric oxide in living organisms,”^[^ ^34^ ^]^ as the great reactivity of the free NO radical requires a carrier for the storage, delivery, transport, and targeted release of NO.^[^ ^35^ ^]^

Various Lewis bases that occupy the two available binding sites of the Fe(NO)_2_ species lead to the well‐known, characteristic EPR signal at g = 2.03 (in the oxidized [Fe(NO)_2_]^+^ form, the paramagnetic {Fe(NO)_2_}^9^ by the Enemark–Feltham notation).^[^ ^36^ ^]^ This signal indicates the presence of {Fe(NO)_2_}^9^ in a variety of biological settings, such as the DNIC‐bound glutathione reductase and particularly in the NO degradation of iron‐sulfur clusters.^[^ ^14^, ^34^, ^35^, ^37^ ^]^

The combination of the DNIU and the MNIU via the thiolate sulfur connection presents as the anti‐ferromagnetically coupled Fe···Fe′ within a dynamical, thermodynamic valley. It self‐assembles according to various synthetic routes, whether by design or by serendipity, dispelling the signals from radicals on both units.^[^ ^33^ ^]^ Most notably, the bridging sulfur platform in this MNIU/DNIU adduct accommodates stability and isolation of Fe_2_(NO)_3_ in three redox levels while maintaining the integrity of the diiron structure.^[^ ^33^ ^]^ The cationic diiron species is the most stable under ambient conditions; nevertheless, the more reactive one‐ and two‐electron reduced analogs are, with care, isolable.^[^ ^32^, ^33^ ^]^ The rich physical/spectroscopic/magnetism probes for iron, NO, N, and S offer an opportunity to understand the complicated electronic structure of such sulfur‐bridged iron nitrosyl systems, and the effects of the redox non‐innocent NO ligand over two metal centers as oxidation/reduction processes take place within the Fe_2_(NO)_3_ complex. Despite considerable prior attention to the common methods of characterization (FTIR analysis, EPR, SC‐XRD, and DFT), a full understanding of the interconnected factors that control the complex interrelationships between stability, structure, electronic, and magnetic properties remains illusive.

Here we report the isolation and characterization of a third member of this family of complexes, based on a dibenzylated N_2_S_2_ ligand. The new redox‐active complexes were further characterized by various techniques (FTIR analysis, EPR, SC‐XRD, and DFT) as well as sulfur K‐edge X‐ray absorption spectroscopy, ^15^N NMR, and Mössbauer spectroscopy. Our results indicate non‐innocent redox characteristics involving both iron and NO in various spin‐coupled arrangements. Unanswered questions/conundrums are clarified through DFT calculations/analysis, and the S‐K‐edge XAS data identify involvement of the bridging sulfur orbitals as a major feature in the charge repository and delocalization between the dissimilar iron centers.

## 2 Results and Discussion

Nitric oxide is well known as a model redox‐active, diatomic ligand, highly responsive to the ligand field of the metal to which it is bound. It can be polarized and chemically extracted from carriers in the form of NO^+^, •NO, or NO^−^. The N_2_S_2_ ligand that accommodates the diiron complex system described above in three synthetically accessible redox levels, [(N_2_S_2_)Fe(NO)•Fe(NO)_2_]^n^, or **[^L^Fe_2_(NO)_3_]** ^n^, n = +1, 0, or −1, has been examined in two N_2_S_2_ backbones, represented as **L1** (*N,N’*‐bis(2‐mercaptoethyl)‐1,5‐diazacycloheptane)) and **L2** (*N,N’*‐bis(2‐mercaptoethyl) diazamethylethane)).^[^ ^32^, ^33^ ^]^ There are multiple synthetic routes leading to the spontaneous assembly of [(N_2_S_2_)Fe(NO)•Fe(NO)_2_]^+^ (Scheme S1). Here we focus on *N,N’*‐bis(2‐mercaptoethyl)‐diazaphenylethane), (**L3)**, used to prepare [(N_2_S_2_)Fe(NO)]. Reaction of the MNIC (mono nitrosyl iron complex) with Fe(CO)_2_(NO)_2_ in dichloromethane at 22 °C yielded the neutral [(N_2_S_2_)Fe(NO)•Fe(NO)_2_]^0^ which was, in a one‐pot reaction, oxidized to the more stable **[^L3^Fe_2_(NO)_3_]^+^** cation by (NO)^+^BF_4_^−^. Figure S1 (Supporting Information) shows the schematic structures of three different ligand systems and their corresponding iron dimers, which provide a visual comparison of their coordination environments. A detailed comparison of the key structural and geometrical parameters, highlighting the differences and similarities among the three systems, which are crucial for understanding how ligand environments influence the geometry and bonding of the iron dimers (see Table S1, Supporting Information).

The cyclic voltammogram recorded for the **[^L3^Fe_2_(NO)_3_]^+^** cation displayed two fully reversible events similar to those previously reported for the **L1** and **L2** ligand systems, Figure S2 (Supporting Information).^[^ ^16^, ^21^ ^]^ The more positive potential at −0.68 V was assigned to the DNIC, {Fe(NO)_2_}^9/10^ couple, and the more negative, −1.26 V to [Fe(NO)]^7/8^. These assignments are consistent with reference compounds, noting that the E_1/2_ of the MNIU in the diiron cation shifts positively from its isolated mono‐iron unit,^[^ ^32^, ^39^ ^]^ and the E_1/2_ of the DNIU is similar to reference complexes of the {Fe(NO)_2_}^9/10^.^[^ ^33^, ^40^ ^]^ That is, the uptake of electrons in the MNIU is facilitated within the diiron complex.

The electrochemical results were considered in choosing an reductant. Stoichiometric amounts (one or two equivalents) of reductants, K^+^HBEt_3_^− ^[1 (m) in THF] or KC_8_, produced neutral and anionic‐charged species, **[^L3^Fe_2_(NO)_3_]^0/−^**, which were isolated from THF solutions at −35 °C. Detailed protocols for the synthesis, isolation, and crystallization of **[^L3^Fe_2_(NO)_3_]^+/0/−^** series are described in the Supporting Information.

### 2.1 Single Crystal X‐Ray Diffraction Studies

The **[^L3^Fe_2_(NO)_3_]^+/0/−^** redox congeners were isolated as crystals and characterized through SC‐XRD techniques; the molecular structures are shown in Figure 2. The full structure reports are given in Supporting Information, including Figures S3–S10 (Supporting Information) for molecular packing and Tables S2–S6 (Supporting Information). CIF files of the structures are available in the Cambridge Crystallographic Database (deposit numbers: 2374364 (the dimeric **(^L3^Fe)_2_** precursor to the nitrosylated species), 2322823, 2326021, and 2374365).

The consistency of the results on physical characterizations for the three ligand platforms, **L1**, **L2**, and **L3**, indicates that the Fe_2_(NO)_3_ unit, as found in the MNIU‐DNIU combination, is a persistent, unified thermodynamic unit of interest for analysis of features that dictate its stability, as well as for further modifications for specific applications. In the three redox congeners, the mono‐iron unit is a square pyramid with NO in the apical position relative to the regular N_2_S_2_ planar base, exposing the cis‐dithiolates for chelation to the Fe(NO)_2_ addendum. In the diamagnetic cationic form, the hinge angle at the bridging thiolate sulfur positions the two iron centers, {Fe(NO)}^7^ and {Fe(NO)_2_}^9^, at a Fe···Fe′ distance of 2.78 Å, consistent with strong antiferromagnetic coupling of two S = ½ units to generate an S = 0 system spin. On addition of an electron, lodging on the dinitrosyl iron unit and rendering it diamagnetic as {Fe(NO)_2_}^10^ (see below), the hinge angle increases and the Fe···Fe′ distance lengthens to 2.91 Å, beyond bonding distance. This exposes the radical on the {Fe(NO)}^7^, which is characterized by EPR spectroscopy and consistent with the overall *S* = ½ system spin for the diiron unit. Despite the localization of the added spin on the DNIU, all three ν(NO) vibrational mode positions, according to computational vibrational mode assignments and site‐specific ^15^NO incorporation, vide infra, are shifted equally to lower wavenumbers, indicating unified vibrational coupling between MNIC and DNIC units, presumably a consequence of a dramatic redistribution of electrons through the Fe(µ‐SR)_2_Fe’ bridge and, considerably onto the NO's.

The second electron added to the neutral [(N_2_S_2_)Fe(NO)•Fe(NO)_2_]^0^, generates anionic [(N_2_S_2_)Fe(NO)•Fe(NO)_2_]^−^, and results in an increase in the Fe···Fe′ distance to 3.13 Å, a change in magnetism from S = ½ to S = 1, as well as a displacement of the iron in the Fe(NO) from ≈0.52 to 0.84 Å out of the best N_2_S_2_ plane. The increased linearity of the Fe‐N‐O angle is consistent with molecular orbital analysis and the assignment of a triplet state, {Fe(NO)}^8^, with the majority spin located on the Fe from a combination of a high‐spin (S = 2) Fe^2+^ and a triplet (S = 1) NO^–^.^[^ ^34^ ^]^ Again, the additional electron density is taken up mainly by the nitrosyls and sulfurs. The paucity of reports of examples of reduced iron mononitrosyl, especially an S = 1 state, {Fe(NO)}^8^, speaks to the stabilizing effect of the {Fe(NO)_2_}^10^ adduct on its neighboring N_2_S_2_ binding site.^[^ ^23^ ^]^ The extra electron density generates routes for oxidative degradation; however, any such products have not been identified.

Interestingly, the **[Fe_2_(NO)_3_]⁺** complex could adopt a higher C_s_ symmetry; however, the bending of the nitrosyls (the two ∠Fe–N–O bond angles in the DNIC units are significantly different, 160.3(8)° and 176.5(8)°, respectively), and the N substituents on the N_2_S_2_ backbone create an unexpected dissymmetry. The Fe1–S1 and Fe1–S2 bond distances are 2.261(3) and 2.259(3) Å, respectively, and Fe2–S1 and Fe2–S2 bond distances are 2.256(3) and 2.242(3) Å, respectively. These structural parameters indicate that the two sulfur atoms are chemically non‐equivalent. This conclusion is further supported by the differing Mulliken charges on the sulfur atoms (see DFT section below).

### 2.2 Infrared ν(NO) Band Assignment

**Figure 3:**
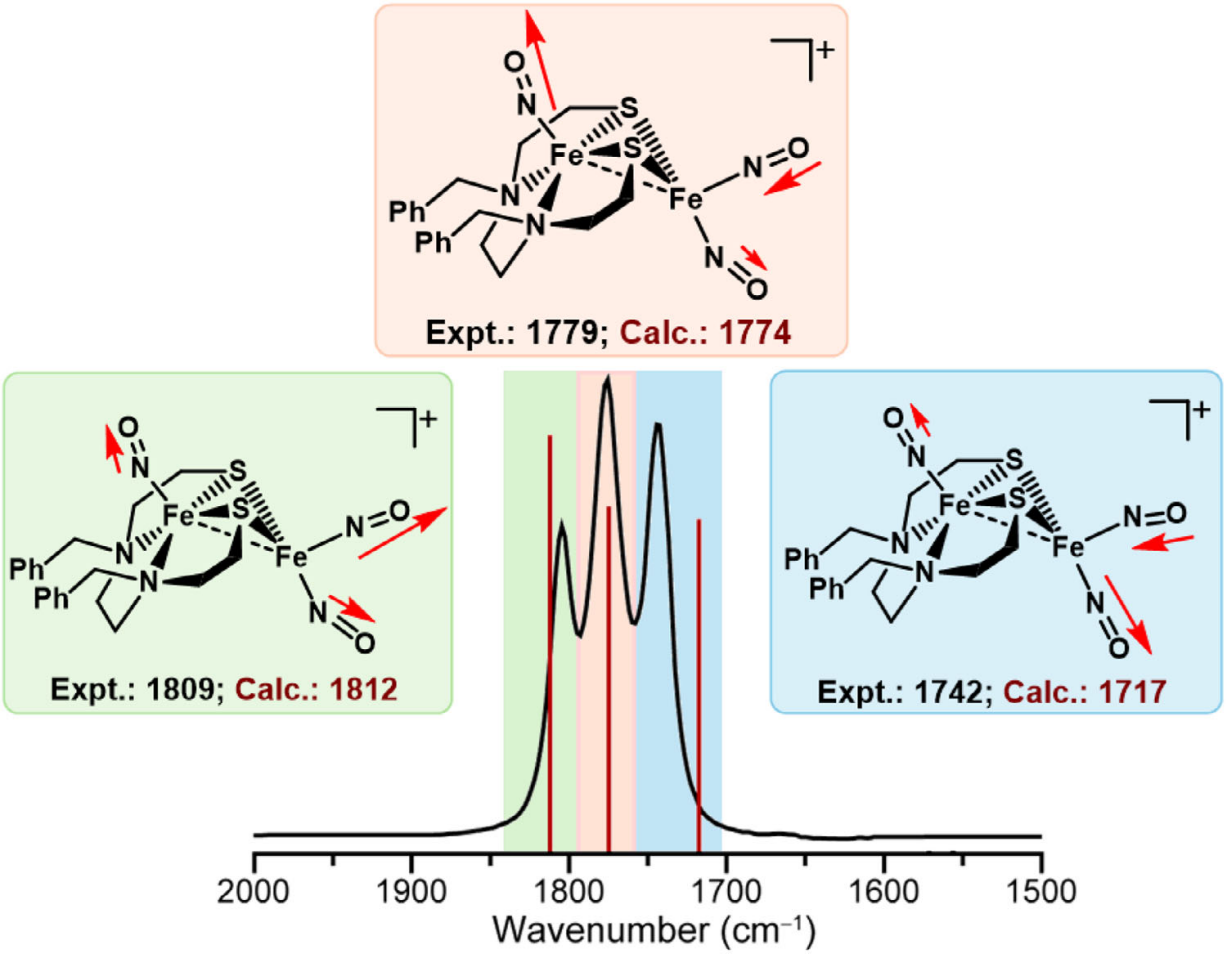
FTIR spectrum of **[** ^**L3**^ **Fe**_**2**_ **(NO**_**3**_ **)]** ^**+**^ with vibrational modes shown; the largest arrow represents the most intense nitrosyl stretch, and smaller stretches and contractions are represented by smaller arrows. The length of the arrows is roughly drawn to scale.

The contributions of the three nitrosyls to the three distinct IR bands or vibrational modes in the cationic species, **[^L^Fe_2_(NO)_3_]^+^** (**L = L1, L2**, and **L3**), were determined by frequency calculations of the optimized ground state structures of the three **[^L^Fe_2_(NO)_3_]^+^** cations. Vibrational modes for the **[^L3^Fe_2_(NO)_3_]^+/0/−^** redox congeners are shown in **Figure** **3** and Figures S11–S13 (Supporting Information). The bands at ≈1809 and 1742 cm^−1^ are the symmetric and asymmetric NO stretching modes of the DNIC unit, respectively, with noteworthy mono‐nitrosyl (MNIC) contributions observed in both.^[^ ^41^ ^]^ Confirmed by site‐specific ^15^NO labeling, vide infra, the central band at 1779 cm^−1^ corresponds largely to the MNIC ν(NO) stretch, with a minor contribution from the asymmetric stretch of the apical NO of the DNIC unit (Figure 3). DFT calculations reproduced IR stretches for all **[^L3^Fe_2_(NO)_3_]^+/0/−^** species; the ν(NO) values closely matching those found by the experiment and the trend of ν(NO) absorption shifts upon reduction (Table S7, Supporting Information). Upon reduction from cation → neutral → anion, the N–O bond distances increase, and bond strength decreases, leading to the decrease in the IR stretching frequencies of NO's across the reduction series (Figure 2; Table S7, Supporting Information).

### 2.3 Site‐Specific ^15^NO Labeling

**Scheme 1:**
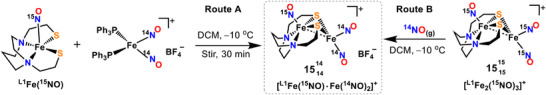
Routes for the synthesis of **[^L1^Fe(^15^NO)∙Fe(^14^NO)_2_]^+^**.

**Figure 4:**
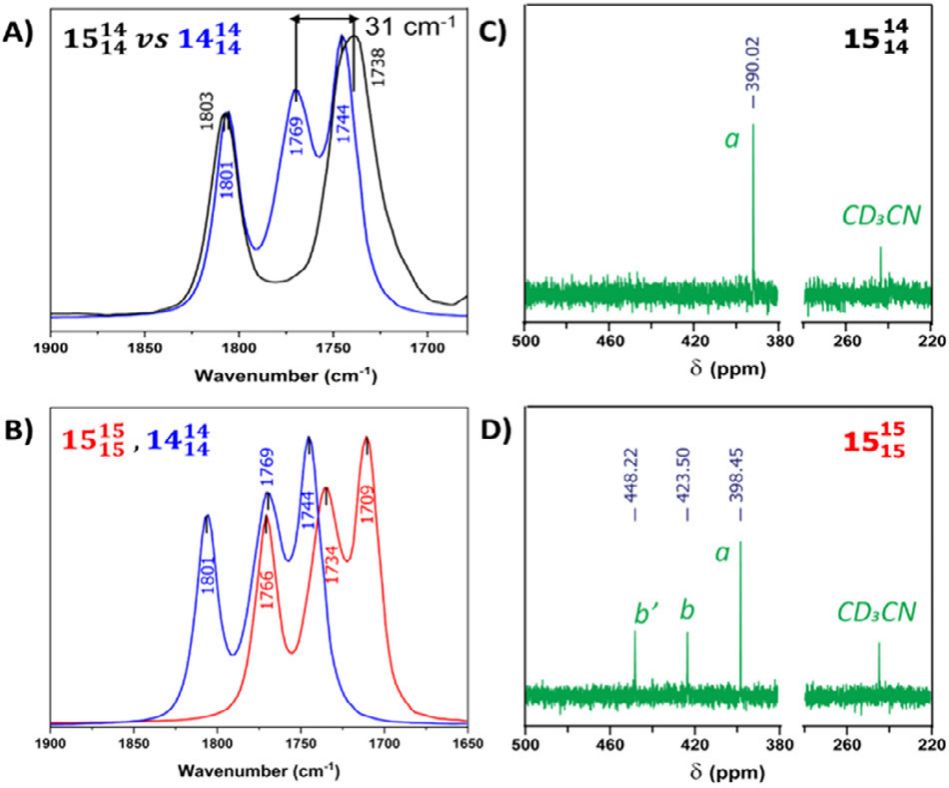
IR spectra of A) **[^L1^Fe(^15^NO)∙Fe(^14^NO)_2_]^+^** and **[^L1^Fe_2_(^14^NO)_3_]^+^**,B) **[^L1^Fe_2_(^14^NO)_3_]^+^** and **[^L1^Fe_2_(^15^NO)_3_]^+^** and N‐15 NMR of C) **[^L1^Fe(^15^NO)∙Fe(^14^NO)_2_]^+^**, D) **[^L3^Fe(^15^NO)_3_]^+^**. The nomenclature of labeled complexes is given in the figures.

To confirm the assignment of the 3‐band ν(NO) spectra, we labeled the mono‐nitrosyl unit of [**^L1^Fe_2_(NO)_3_]^+^** with ^15^NO. The reaction shown in **Scheme** **1**, Route A, occurs at –10 °C and utilizes an {Fe(NO)_2_}^9^ source configured with two labile triphenylphosphine ligands,^[^ ^42^ ^]^ which are readily replaced by the ^15^NO‐labeled bidentate metallodithiolate, **^L1^Fe(^15^NO**). Mass spectroscopy noted only one position for the ^15^NO label, assigned as the mononitrosyliron, consistent with the ν(NO) IR data (**Figure** **4**A; Figures S14, Supporting Information). By another route, the controlled addition of ^14^NO_(g)_ to the dichloromethane solution of all N‐15 **[^L1^Fe_2_(^15^NO)_3_]^+^** at –10 °C also produced the single ^15^NO‐labeled **[^L1^Fe(^15^NO)·Fe(^14^NO)_2_]^+^** species (Scheme 1, Route B). In that the MNIU label is retained, we conclude that, even at −10 °C, there is greater NO lability within the DNIU.

Figure 4A displays the IR spectrum obtained from the site‐specific ^15^NO labeled product shown in Scheme 1, confirming that the central IR band of the 3‐band ν(NO) spectrum arises from the mono‐nitrosyl unit of the **[^L1^Fe_2_(NO)_3_]^+^** species, as had been concluded from the analysis of the all N‐14 species described above. The IR patterns remained the same at −10 °C; however, at room temperature, the ^15^NO in the metallodithiolate ligand exchanges with the ^14^NO in Fe(NO)_2_ (Figure S15, Supporting Information). A similar spectral signature was observed immediately after mixing the **[^L1^Fe_2_(^14^NO)_3_]^+^** with all **^15^NO** labeled, i.e., **[^L1^Fe_2_(^15^NO)_3_]^+^**, in CH_2_Cl_2_; at room temperature, the pattern remained the same up to 12 h (Figure S16, Supporting Information). That means NO scrambling is very fast at RT; ESI‐mass spectrometry of the isolated products was consistent with this conclusion (Figure S17, Supporting Information). However, whether an inter‐ or intramolecular mechanism applies to the NO exchange is uncertain. Figure 4B compares the IR spectra of all 15‐N and all 14‐N cationic species.

### 2.4 ^15^N Nuclear Magnetic Resonance Spectroscopy and NO Scrambling

The ^15^NO‐labeled cationic complexes (**[^L1^Fe_2_(^15^NO)_3_]^+^**, **[^L2^Fe_2_(^15^NO)_3_]^+^**, **[^L3^Fe_2_(^15^NO)_3_]^+^**, and **[^L1^Fe(^15^NO)∙Fe(^14^NO)_2_]^+^**) give access to characterization by ^15^N (I = ½) NMR spectroscopy.^[^ ^43^ ^]^ The selectively labeled **[^L1^Fe(^15^NO)∙Fe(^14^NO)_2_]^+^** identifies the MNIU signal at 390.02 ppm relative to the CD_3_CN standard^[^ ^44^ ^]^ (Figure 4C). All fully labeled complexes (containing three ^15^NO) show three signals in their N‐15 NMR spectra. The **[^L1^Fe_2_(^15^NO)_3_]^+^**, **[^L2^Fe_2_(^15^NO)_3_]^+^**, and **[^L3^Fe_2_(^15^NO)_3_]^+^** show one resonance generated from labeled MNIU, ({Fe(^15^NO)}^7^) and two generated from the labeled DNIU ({Fe(^15^NO)_2_}^9^) (Figure 4D; Figure S18, Supporting Information).

The N‐15 NMR resonance positions for the cationic diiron complexes are tabulated in Table S8 (Supporting Information). From this measure, we note that there is consistency in ligand effects on chemical shifts in both the Fe(NO) and Fe(NO)_2_ units; discernible changes in peak positions are observed and the order of up‐field shifting in peak position varies as: **[^L3^Fe_2_(^15^NO)_3_]^+^** < **[^L2^Fe_2_(^15^NO)_3_]^+^** < **[^L1^Fe_2_(^15^NO)_3_]^+^**. Note that the signals from the MNIU appear in a more shielded region than those from the DNIU. A simple interpretation of this observation is that the {Fe(NO)}^7^ (MNIU) has more electron density on NO as it is embedded within the 8‐electron‐donating N_2_S_2_ pocket. Moreover, the additional *π*−delocalization within the DNIU is expected to lead to greater de‐shielding.

### 2.5 Magnetic Susceptibility Measurements and EPR Results

Application of the Evans method for the determination of *µ*_eff_ in solution at room temperature (22 °C) (see Supporting Information) provides a value of ≈1.78 BM for **[^L3^Fe_2_(NO)_3_]^0^** and ≈2.91 BM for the **[^L3^Fe_2_(NO)_3_]^−^** species (Figures S19 and S20, Supporting Information). The former *µ*_eff_ implies that **[^L3^Fe_2_(NO)_3_]** **^0^** contains one unpaired electron, i.e., an *S* = ½ spin state system, and the latter value ≈2.91 BM suggests **[^L3^Fe_2_(NO)_3_]^−^** contains two unpaired electrons, i.e., an *S* = 1 spin state system. In contrast, the cationic **[^L3^Fe_2_(NO)_3_]^+^** species was found to be diamagnetic. These results are consistent with those obtained for the individual redox congeners of all **[^L^Fe_2_(NO)_3_]** thus far studied.^[^ ^32^, ^33^ ^]^

The magnetic susceptibility results are corroborated by EPR measurements. The anti‐ferromagnetic coupling of the two radicals from {Fe(NO)}^7^ and {Fe(NO)_2_}^9^ at Fe···Fe′ (2.78Å) in the cationic species **[^L3^Fe_2_(NO)_3_]^+^** renders it EPR silent (Figure S21, Supporting Information). The neutral species, **[^L3^Fe_2_(NO)_3_]^0^**, presents a *g*_iso_ = 2.02 signal, confirming the *S* = ½ spin state as expected from the {Fe(NO)}^7^ unit when the DNIU becomes spin‐paired {Fe(NO)_2_}^10^ (Figure S22, Supporting Information). Upon further one‐electron reduction of **[^L3^Fe_2_(NO)_3_]^0^**, the peak intensity at *g* = 2.02 approaches zero (Figure S23, Supporting Information), predictive of the reduced species (**[^L3^Fe_2_(NO)_3_]^−^**) having an integer spin (*S* = 1), which is not detectable in perpendicular mode EPR spectroscopy. However, in the parallel EPR, the analogous species, **[^L2^Fe_2_(NO)_3_]^0^**, exhibits a signal similar to that observed, while the two‐electron reduced anionic species, **[^L2^Fe_2_(NO)_3_]^−^**, displays EPR features in perpendicular EPR mode that were assigned to the S = 1 state.^[^ ^33^ ^]^ The delocalization of an unpaired electron in the neutral **[^L2^Fe_2_(NO)_3_]^0^** complex between (N_2_S_2_)Fe(NO) and Fe(NO)_2_ units was shown in the previous report.^[^ ^33^ ^]^ The spin density plot of the neutral **[^L3^Fe_2_(NO)_3_]^0^** and anionic **[^L3^Fe_2_(NO)_3_]^−^** species here (Figure S24, Supporting Information) is largely the same as in the earlier study, see discussion in Hall et al.^[^ ^33^ ^]^

### 2.6 Mössbauer (MB) Spectroscopy

**Figure 5:**
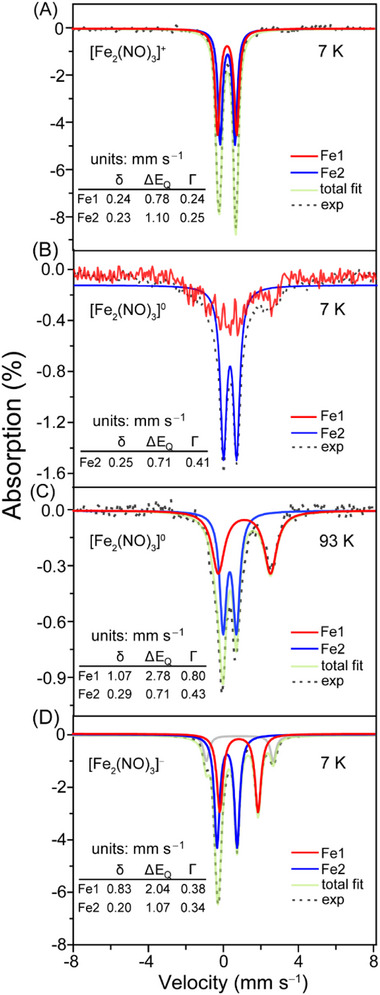
Low‐field Mossbauer Spectra of A) **[^L3^Fe_2_(NO)_3_]^+^**, B) **[^L3^Fe_2_(NO)_3_]^0^** at 7 K, C) **[^L3^Fe_2_(NO)_3_]^0^** at 93 K, and D) **[^L3^Fe_2_(NO)_3_]^‐^** at 7 K; L3 = (N,N’‐bis(2‐mercaptoethyl)‐diazaphenylethane)). The gray solid line in D is assigned to an undefined impurity.

Spectra of concentrated solutions of the **[^L3^Fe_2_(NO)_3_]^+/0/−^** redox series were collected (CH_3_CN for cationic species and THF for neutral and anionic species) at 5–7 K and 0.05 T, and quadrupole doublets were simulated using WMOSS4 software.^[^ ^45^ ^]^ The cation exhibited two narrow doublets (**Figure** **5**A), each representing ≈ 50% of the overall intensity. Doublet I (red line) has an isomer shift δ = 0.24 mm s^−1^ and a quadrupole splitting ∆E_Q_ = 0.78 mm s^−1^. Doublet II (blue line) has δ = 0.23 mm/s and ∆E_Q_ = 1.10 mm s^−1^. The two sets of parameters are sufficiently similar to suggest a common effective (average) oxidation state for both irons.^[^ ^46^, ^47^ ^]^

The sample of the one‐electron‐reduced S = ½ neutral state was most difficult to handle, and each of the 5 preparations exhibited somewhat different Mössbauer spectra (These are described in Supporting Information, Mössbauer section.) In all cases, the spectral properties of one iron, to be called **Fe_red_** (Fe1), changed significantly, while those of the other iron, to be called **Fe_ox_** (Fe2), changed minorly. Each Fe represented ≈ 50% of the overall spectral intensity. In all spectra, **Fe_red_** exhibited significant magnetic hyperfine interactions at 5–7 K (Fe1 in Figure 5B; Figure S25, Supporting Information). Figure 5B; the subtraction of **Fe_ox_** from the overall spectrum reveals the spectral contribution of the **Fe_red_** center. At high temperature (93 K, Figure 5C), the hyperfine collapsed, revealing a broad doublet with isomer shift and quadrupole splitting values of δ = 1.07 mm s^−1^ and ∆E_Q_ = 2.78 mm s^−1^. In all spectra of the neutral species, **Fe_ox_** exhibited minor changes in isomer shift or quadrupole splitting, relative to the diamagnetic cationic state, namely to δ = 0.29 mm s^−1^ and ∆E_Q_ = 0.71 mm s^−1^ (Fe2 in Figure 5B,C). We conclude that **Fe_red_** is reduced in the neutral state relative to the cationic state, whereas **Fe_ox_** remained in the same effective oxidation state as in the cationic state. Spectral variations among neutral samples included whether **Fe_ox_** exhibited magnetic hyperfine interactions at low temperatures, and uncertainty as to the temperature at which such interactions in **Fe_red_** collapsed (see Figure S25, Supporting Information).

Two spin‐coupling scenarios seem consistent with the MB observations for the neutral species. In both scenarios, the system spin is S = ½ and the local spin of the **Fe_ox_** group (Fe including the bound NO/NO's) is S = ½. In Scenario 1, the **Fe_red_** group has a local spin S = 1 and the system is antiferromagnetically coupled to the **Fe_ox_** group (S = ½) to yield an overall spin S = ½. In Scenario 2, the **Fe_red_** group has local spin S = 0.

The corresponding 7 K MB spectrum of the anionic species **[^L3^Fe_2_(NO)_3_]^−^** exhibited two major doublets (Figure 5D) and a third 10% intensity doublet that is likely reflecting a contaminant. One doublet, with 40% overall intensity, had parameters similar to that of **Fe_red_** in the neutral spectrum, namely δ = 0.83 mm s^−1^; ∆E_Q_ = 2.04 mm s^−1^.

However, that doublet was significantly sharper (Linewidth, Γ = 0.80 mm s^−1^ for that of the neutral species, and 0.38 mm s^−1^ for the anion species). We conclude that the effective oxidation state of that iron was unchanged relative to **Fe_red_**. The other doublet, representing ≈50% of spectral intensity, had parameters similar to that of **Fe_ox_** of the neutral state, with δ = 0.20 mm s^−1^ and ∆E_Q_ = 1.07 mm s^−1^. Again, we assumed that this iron has the same effective oxidation state as **Fe_ox_** in the neutral state. The major difference, relative to the spectrum of the neutral species, was that neither doublet exhibited magnetic hyperfine interactions, suggesting either a diamagnetic system spin, an antiferromagnetically coupled system spin with S = 0, or perhaps an integer spin state. Extending Scenario 1 above, the **Fe_red_** group (MNIU) in the neutral species remains S = 1, whereas the **Fe_ox_** group (DNIU) might be reduced to generate an S = 0 spin state (Fe and NO coupled). Extending Scenario 2 above, **Fe_red_** might remain S = 0, while **Fe_ox_** might be reduced to generate an S = 1 spin state (Fe and NO coupled). Accordingly, for both Scenarios, the system spin of the anion would be S = 1, as observed by magnetic susceptibility. Our electrochemistry and infrared results, and DFT calculations, vide infra, suggest that **Fe_red_** in the neutral species originates from the DNIC fragment and **Fe_ox_** from the MNIU fragment, an assignment that is consistent with Scenario 2. In both scenarios, electrons would need to be shifted to the NO and sulfurs in order to maintain an essentially unchanged MB shift.

### 2.7 X‐Ray Absorption Spectroscopy (XAS)

In a series of studies from the Solomon/Hodgson/Hedman group, sulfur K‐edge XAS was used to investigate the electronic structure of non‐heme iron in {Fe(NO)}^7^ systems. A monomeric (N_2_S_2_)Fe(NO) complex was contrasted to that in the six‐coordinate (N_4_S)Fe(NO); both of these, in ligand‐reduced forms, are related to Fe(O_2_) systems.^[^ ^48^, ^49^ ^]^ The complexities of electron assignment are amplified in the diiron complexes studied here, in that the {Fe(NO)}^7^, the monoiron nitrosyl, MNIU, is bridged by thiolate sulfur to a second iron within a dinitrosyl iron unit. The DNIU provides even more electronic structure possibilities with unknown effect on the MNIU side of the Fe_2_(NO)_3_. The DNIU, whether in oxidized {Fe(NO)_2_}^9^ or reduced {Fe(NO)_2_}^10^ forms, competes with the (N_2_S_2_)Fe(NO) for the sulfur electron density needed by the MNIC. The shifts in positions of the pre‐edge and following edge absorbances, even if slight, are ill‐defined but are to be expected.

**Figure 6:**
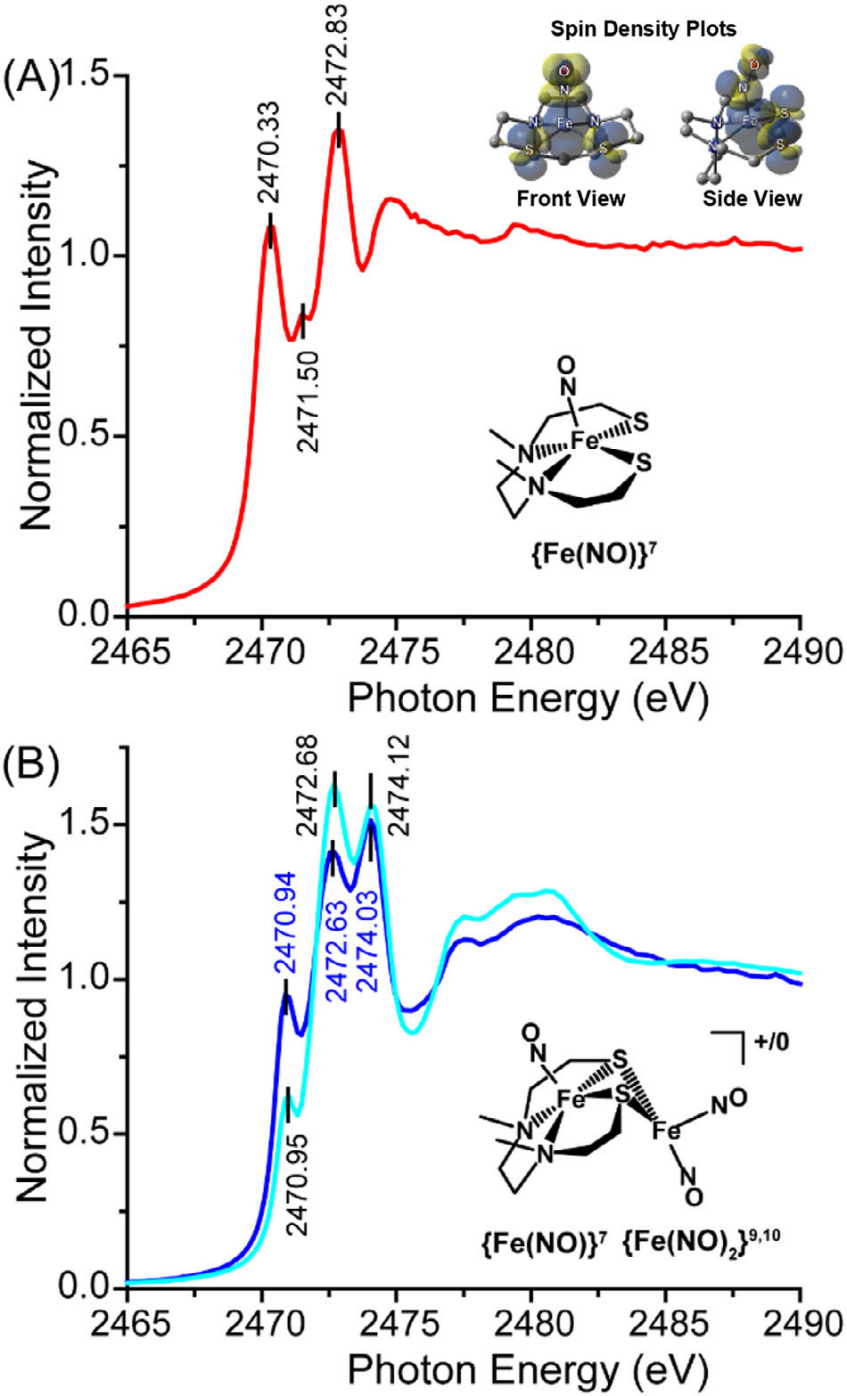
S K‐edge XAS spectra of A) **^L2^Fe(NO)**  and B) **[^L2^Fe_2_(NO)_3_]^+^** (blue line), and **[^L2^Fe_2_(NO)_3_]^0^** (cyan line).

The conclusions from the Solomon et al. study were based on an N_2_S_2_ framework that is only slightly different from the one in our current study. The pre‐edge features (≈2470 eV) and the two subsequent absorptions (≈2472, ≈2474 eV) are very similar. The complexity in the spin‐density plots shown in the insert of **Figure** **6**A, emphasizes the covalency of the S‐Fe(NO)S manifold of orbital overlap. As the S pre‐edge energy derives from the oxidation state of the iron and the energy splitting patterns of the pre‐edge transition reflect spin state, we can assume that our MNIC has electronic properties substantially identical to the reported one.^[^ ^48^ ^]^ The interpretation by Solomon et al., supported by theory, is the starting point of our interpretation of the sulfur‐bridged Fe_2_(NO)_3_.

Serving as a reference point for the diiron complexes, the isolated monomeric ^L2^Fe(NO) species was found to exhibit two pre‐edge features at 2470.3 and one of very low intensity, at 2471.5 eV derived from the S_1s_ → Fe_3d_ transition; the former shows significantly higher intensity compared to the latter. The feature at 2472.8 eV, Figure 6A, is assigned to the S K‐edge absorption, i.e., S_1s_ → S_C–S σ*_. Both cationic **[^L2^Fe_2_(NO)_3_]^+^ **and neutral **[^L2^Fe_2_(NO)_3_]^0^** diiron complexes show two distinct, but similar edge features, one at 2472.6/2472.7 and a second at 2474/2474.1 eV, Figure 6B. These S K‐edge peaks are derived from S_1s_ → S_C–S σ*_ transitions. The two diiron species display practically identical pre‐edge transitions at 2470.9/2471 eV, respectively. They have lost the low‐intensity feature seen for the reference monomeric ^L2^Fe(NO), and in both redox levels examined for the diiron species, the observable single feature is slightly shifted to higher energies.^[^ ^35^, ^36^ ^]^ The shift to higher energy is indicative of the influence of the second iron from the DNIC, which in this case renders both irons within the diiron unit to be virtually identical regardless of overall charge on the **[^L2^Fe_2_(NO)_3_]^+/0^**, however, the sulfurs are slightly different, *vide infa*. We then conclude that the 2071 eV feature that was observed for the monomeric ^L2^Fe(NO) species is hidden underneath the absorption at 2472 eV seen for the diiron complexes.

The TDDFT calculated S K‐edge XAS spectra are shown in Figure S26 (Supporting Information). When the **[^L2^Fe_2_(NO)_3_]^+^** is one‐electron reduced to the neutral compound, the pre‐edge intensity decreases significantly. Presumably, this is because the added electron is placed in the originally unoccupied orbitals with Fe_d_‐S_p_ σ^*^ contributions.

### 2.8 Computational Modeling

Geometrical structural parameters, vibrational frequencies, and energies of the **[^L3^Fe_2_(NO)_3_]^+/0/−^** complexes were calculated by DFT methodologies with Gaussian 16, Version D01.^[^ ^50^, ^51^ ^]^ Both the TPSS functional and the TPSSh functional, a hybrid functional with 10% Hartree–Fock exchange, were employed.^[^ ^52^ ^]^ The 6‐311++G(d,p) basis set was used for non‐metal atoms, while the Wachters–Hay basis set under the designation 6‐311++G(d,p),^[^ ^52^, ^53^, ^54^, ^55^ ^]^ was used for iron. Calculations were made at the crystal structure geometries and with full geometric optimization in vacuum, and the nature of each optimized stationary point was verified by frequency calculations to have appropriate numbers of imaginary vibrational modes. The TPSS functional shows less spin‐contamination for the three redox species compared to the TPSSh functional (Table S9, Supporting Information).

For the cationic species, **[^L3^Fe_2_(NO)_3_]^+^**, computations predict the broken symmetry (BS) singlet (M_s_ = 0, S = 0.22), to be 0.83 kcal mol^−1^ more stable than the closed‐shell (CS) singlet (S = 0), and 10.26 kcal mol^−1^ more stable than the triplet (M_s_ = 1, S = 1.04). The BS singlet state exhibits antiferromagnetically coupled {Fe(NO)}^7^ and {Fe(NO)_2_}^9^ spin centers, with the iron atoms separated by 2.623 Å (expt'l is 2.780 Å). For the neutral complex, the doublet (M_S_ = ½, S = 0.51) state is 4.4 kcal mol^−1^ more stable than the higher spin state (M_S_ = 3/2); for the anionic complex, the triplet (M_S_ = 1, S = 1.08) is 16.9 kcal mol^−1^ more stable than the CS singlet (S = 0) state. Calculated structural parameters of **[^L3^Fe_2_(NO)_3_]^+/0/−^** are well matched with the experimental results (Table S10, Supporting Information).

**Figure 7:**
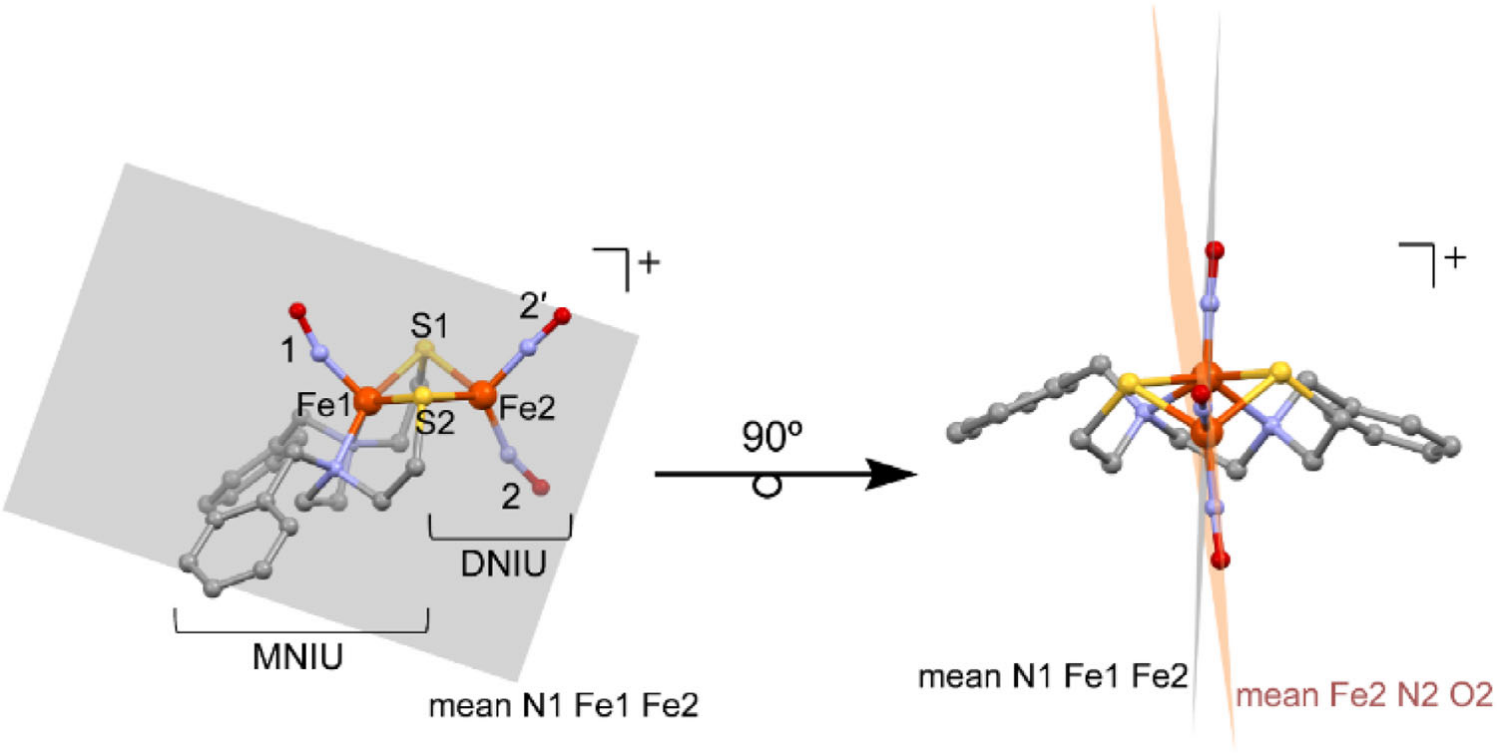
Structure of **[^L3^Fe_2_(NO)_3_]^+^** derived from XRD parameters overlaid by the gray transparent N1‐Fe1‐Fe2 plane. Clockwise rotation by 90° emphasizes the slight displacement or out‐of‐plane positions of Fe‐N‐O interplanar dihedral angles.

Mulliken charges and spin populations from the TPSS and TPSSh calculations, displayed in **Table** **1** and Table S11 (Supporting Information), respectively, are assigned to individual atoms/diatomic and collected into separate MNIU and DNIU values. The bridging sulfur values are shared equally by the two units, tabulated in columns 1 and 2 (Table 1), respectively, according to the designation shown in **Figure** **7**. As electrons are added, the two fragments MNIU and DNIU split the additional negative charge nearly equally. For both the MNIU and the DNIU, most of the additional charge is taken up by the NO and sulfur.

**Table 1:** Mulliken charge and spin population of **[^L3^Fe_2_(NO)_3_]^+/0/‐^** complexes calculated from single‐point calculation using the TPSS functional. Identification of atoms given in Figure 7.

*Mulliken charge*	MNIU^a^ ^)^	DNIU^a^ ^)^	Fe1	[NO]1	Fe2	[NO]2	[NO]2′	S1	S2
**[^L3^Fe_2_(NO)_3_]^+^**	1.12	−0.12	2.06	−0.32	1.03	−0.27	−0.28	−0.46	−0.73
**[^L3^Fe_2_(NO)_3_]^0^**	0.62	−0.62	2.14	−0.26	1.36	−0.42	−0.52	−0.99	−1.08
**[^L3^Fe_2_(NO)_3_]^−^**	−0.14	−0.86	2.29	−0.87	0.71	−0.45	−0.49	−0.48	−0.76
*Mulliken spin population*									
**[^L3^Fe_2_(NO)_3_]^+^**	0.45	−0.44	0.50	−0.01	−0.70	0.14	0.13	−0.02	−0.02
**[^L3^Fe_2_(NO)_3_]^0^**	0.79	0.21	0.80	−0.02	0.20	0.02	−0.03	0.03	0.04
**[^L3^Fe_2_(NO)_3_]^−^**	1.81	0.19	2.25	−0.61	0.08	0.03	−0.03	0.11	0.09

^a)^ The sums of charges and spins of sulfur 1 and sulfur 2 are split between Fe1(all) and Fe2(all) defined as in the brackets above.

Note that Fe1 has a positive charge in all three redox levels; in fact, it increases slightly with each reduction; significant structural changes at Fe1 account for this non‐intuitive result. Note, however, that. the NO of Fe1‐NO becomes more negative with each reduction. Compared to **[^L3^Fe_2_(NO)_3_]^+^**, the ∠Fe‐N‐O angle in **[^L3^Fe_2_(NO)_3_]^0^** decreases significantly, then upon reduction to **[^L3^Fe_2_(NO)_3_]^‐^**, the Fe1(NO) unit moves out of the N_2_S_2_ plane by an additional ≈0.3 Å, and the ∠Fe‐N‐O angle becomes more linear. Thus, the total charge on FeNO in the MNIU changes very little over the two‐electron reduction: 1.74, 1.88, and 1.42. In the **[^L3^Fe_2_(NO)_3_]^+/0/−^** series, the two sulfurs are different, shown in Figure 7. Although the simple rendition of the crystal **[^L3^Fe_2_(NO)_3_]^+/0/−^** series in Figure 2 appear to have a mirror plane containing Fe(NO)_2_ and Fe(NO), splitting the ∠N‐Fe‐N and ∠S‐Fe‐S angles, closer analysis finds a minor dissymmetry shown in Figure 7; the planes containing N1 Fe1 Fe2 and Fe1 Fe2 N2 have a dihedral angle of 8.36° for the cationic species.

**Figure 8:**
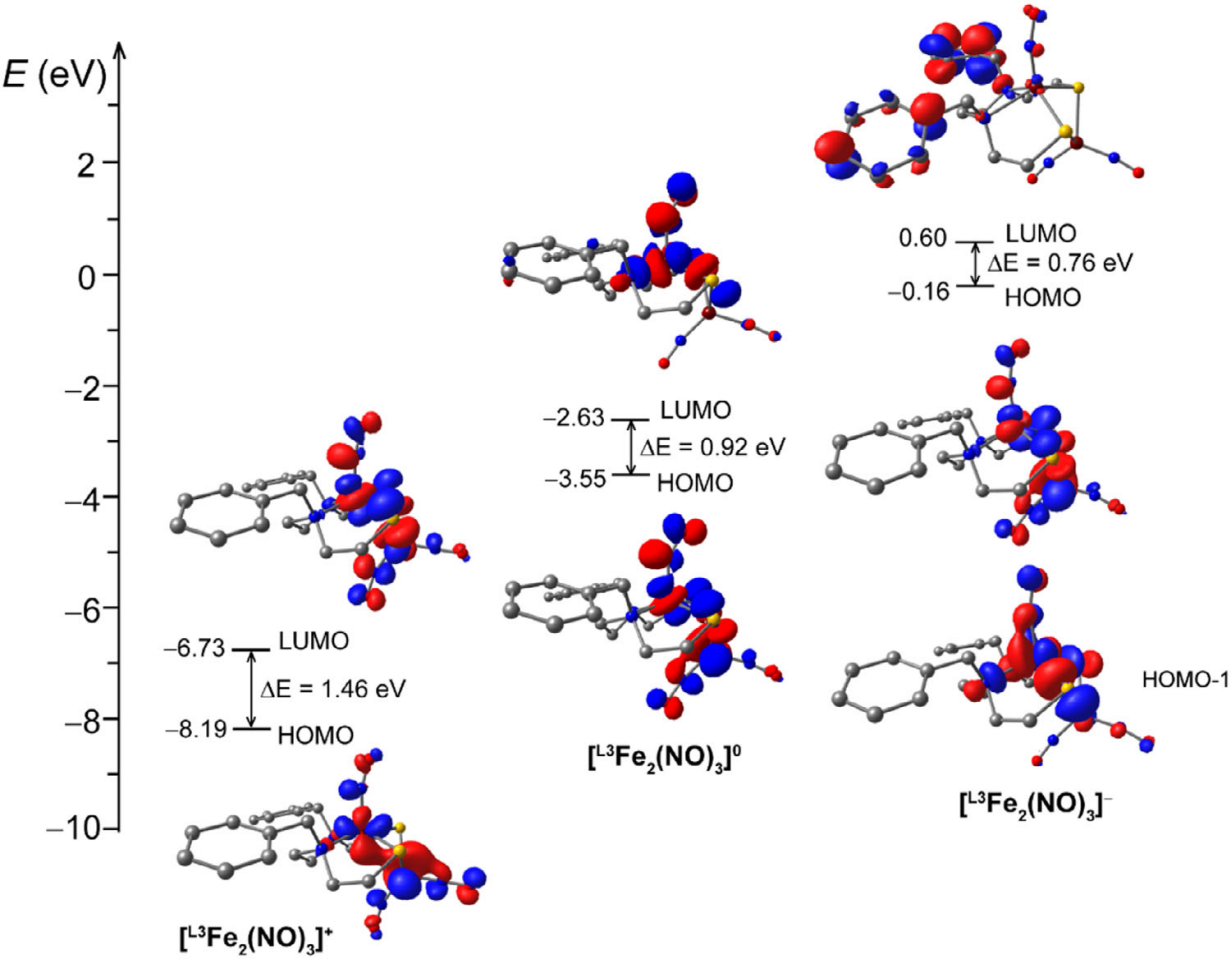
Energy diagrams and selected Kohn–Sham orbitals^[^ ^56^ ^]^ of **[^L3^Fe_2_(NO)_3_]^+/0/‐^** series (contour value = 0.04).

The frontier molecular orbitals are frequently thought of as constructs that define modifications in the valence electron occupations upon reduction; however, there may be significant rearrangement in the orbital structure, in part arising from the geometric changes, for example, the Fe···Fe distances and the ∠Fe‐N‐O bond angle. As shown in **Figure** **8**, the LUMO of the cationic species is indeed comparable to the HOMO of the neutral species, but the LUMO of the neutral species is not the HOMO of the anionic species; however, it appears similar to the HOMO‐1 (Figure 8). The alpha and beta HOMOs and LUMOs for the **[^L3^Fe_2_(NO)_3_]^+/0/−^** series are provided in the Tables S12–S14 (Supporting Information). Significantly, the most electropositive center in the diiron construct is in all cases the mononitrosyl, Fe(NO), with a nearly constant charge. Yet the thermodynamically preferred position for the first‐added electron is to the {Fe(NO)_2_}^9^. Having saturated the orbitals of the DNIU, the second added electron must then go to the {Fe(NO)}^7^. With frontier orbitals of both iron nitrosyls satisfied, the LUMO of the anion resides on the benzyl substituents of the ligand framework. Overall, the calculations support Scenario 2 as described in the ***Mössbauer (MB) spectroscopy*** section, and as assumed in earlier studies. They also account for the uniform shifting of the ν(NO) IR bands upon addition of electrons, suggesting that the nitrosyl and sulfur groups distribute the added electron density significantly, and the latter share and transfer electrons between the two Fe sites.

## 3 Conclusion

**Figure 9:**
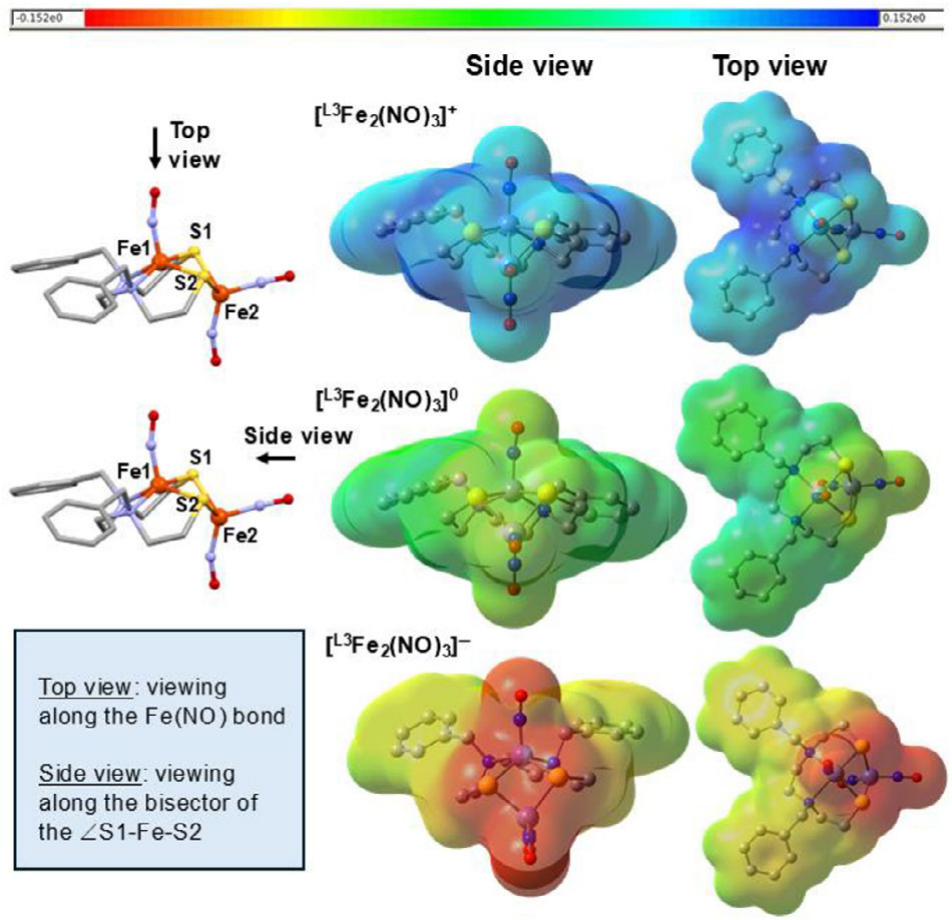
Molecular electrostatic potential (MEP) maps of **[^L3^Fe_2_(NO)_3_]^+/0/−^** calculated using TPSS/6‐311++G(d,p) level. Contour = 0.001, and the same color scale used for all the maps.

Despite widespread agreement that many transition metal nitrosyls, especially iron nitrosyls, comprise highly covalent M‐N‐O interactions which do not easily yield to deconvolution into classical localized oxidation states, efforts continue to be directed toward a more definite identification of the redox state(s) of their components, particularly the chemically and biochemically ubiquitous dinitrosyl iron unit, DNIU, as well as the mononitrosyl iron, MNIC. The convenient Enemark‐Feltham notation (for redox levels of typical MNIC, {Fe(NO)}^7/8^, and typical DNIC {Fe(NO)_2_}^9/10^) are useful starting points in this regard, but as spectroscopic and computational tools progress, the scientific community is more qualified, and determined, to address the ambiguity associated with specific electronic distribution in MNICs and DNICs. With a tetrahedral primary coordination environment completed with harder (i.e., smaller and less polarizable) ligands like phenoxide, alkoxides, and imidazoles, the DNIU, {Fe(NO)_2_}^9^, is typically defined as an Fe^III^ coordinated to two spin‐coupled nitroxyl (NO¯) ligands, with an EPR active S = ½ signal ≈g = 2.03.^[^ ^57^, ^58^ ^]^ With greater covalency in the primary coordination environment (i.e., larger and more polarizable S‐donor thiolate ligands), the {Fe(NO)_2_}^9^ may be considered as a resonance hybrid between two states: Fe^III^(NO¯)(NO¯)↔Fe^II^(NO·)(NO¯), based on the electronic occupancy of the NO ligands as determined by V2C XES^[^ ^46^ ^]^ and the oxidation state of iron as determined by XAS.^[^ ^59^ ^]^ Clearly, the spectral features are intricate, as are assignments. In our (µ‐S)_2_Fe_2_(NO)_3_ system, the overall ligand effect is profound and adjustable by the redox level of the metallo‐ligand field. Information from multiple physical techniques and correlation via theoretical (DFT) analysis, summarized in the electrostatic potential map plots, **Figure** **9**, converges on the conclusion of extensive delocalization of charge on two irons, three nitrosyls, and, most definitely, the two sulfurs. The immediate takeaway meaning of such delocalization is the obvious stability of the structural platform for which the useful Enemark–Felton notation for individual iron nitrosyl centers might be expanded, merging the Fe(NO) and Fe(NO)_2_ units into {Fe_2_(NO)_3_}^16,17,18^ for cation, neutral, and anionic units, respectively. While the expanded E‐F notation recognizes the delocalization of charge, it overlooks the localization of spin, and it cannot address the extensive role(s) of sulfur. The capability of the two dithiolate sulfur bridges to balance between 2‐ and 4‐electron bridge donors, each calling on a subset of its 4 lone pairs is not quantitatively established; XAS experiments of an expanded set of related complexes are needed in future studies. Currently we surmise that the donor/acceptor qualities of sulfur in the Fe(µ‐SR)_2_Fe’ bridges are a dynamic, ill‐defined flux, likely accounting for the unified shift in ν(NO) IR values for the one‐electron reductions in the series.

**Figure 10:**
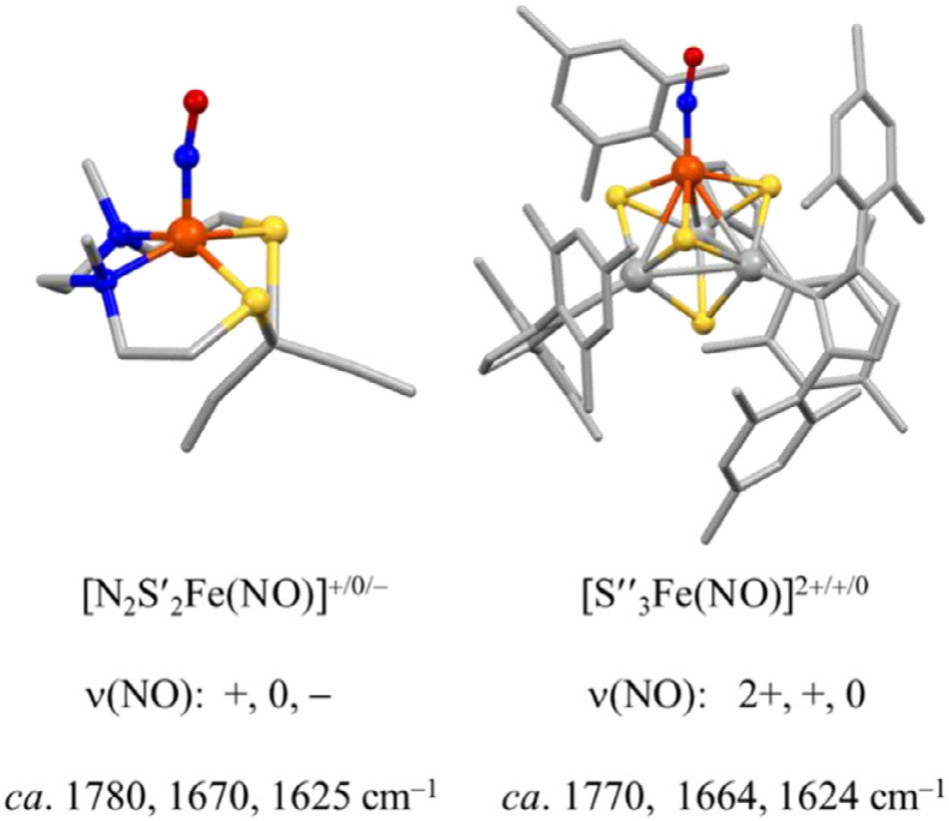
Structure emphasizing the MNIU within the **Fe_2_(NO)_3_** complex of this study and that in Suess's site differentiated the 4Fe4S manifold.

An interesting comparison of the electron‐delocalizing ability arising from the appended DNIU in the Fe_2_(NO)_3_ system explored above can be made with a 4Fe4S cube‐cluster in which three irons are site‐differentiated by bulky carbene ligands (**Figure** **10**), while the fourth is available to hold a nitrosyl, [L_3_Fe_3_S_4_Fe′(NO)]^n^.^[^ ^60^ ^]^ This species, a model of the nitrosylated [Fe_4_S_4_] cluster in Endonuclease III,^[^ ^14^ ^]^ was characterized in three charge levels, *n* = 0, +1, 2+, finding ν(NO) infrared shifts from ≈1624 to 1664 to 1770 cm^−1^, concomitant with spin changes from S = ½ to S = 1, and, in the di‐cation, returning to S = ½. The two Fe(NO) coordination environments, N_2_S_2_ in our studies, versus S_3_ in the Suess complex, are each adaptable by the addenda to the S, rendering the spectroscopic signals sensitive to the immediate donor atoms in the ligand field, as well as the further attachments to those donor atoms. In our trinitrosyl complex, the ν(NO) signals from the mononitrosyl iron side, Fe(NO), are surprisingly similar, accountable to delocalization by quite different secondary coordination spheres mediated by sulfur. It is significant that, from the DFT analysis, the electron charges at the bridging thiolate sulfurs in the ^L3^Fe_2_(NO)_3_ increase as much or more than the components within the Fe(NO) manifold. A more recent success in the biomimicry of 4Fe4S‐bound NO has used a tris‐thiolate ligand that holds the cluster similarly to the cysteinate arrangement in biology.^[^ ^60^, ^61^ ^]^

Related to this report, the famous Roussin's Red Ester (RRE), a dimeric DNIC, [RSFe(NO)_2_]_2_, has also been studied in three (0, 1−, 2−) redox levels.^[^ ^62^, ^63^ ^]^ Rather than the hinge at the bridging thiolates, as in the S_2_Fe_2_(NO)_3_ system, the 2Fe2S core is a planar rhomb in RRE, hinting at a greater delocalization of electron density. Indeed the structural differences relating to the redox levels traverse Fe···Fe separations of 2.69Å (the neutral {Fe(NO)_2_}^9^{Fe(NO)_2_}^9^), to 2.85Å in the mono‐anion {Fe(NO)_2_}^9^{Fe(NO)_2_}^10^, to 3.61Å in the dianion, {Fe(NO)_2_}^10^{Fe(NO)_2_}^10^ species with corresponding ∠Fe‐S‐Fe angle enlarging concomitantly. As the majority of the frontier electron density is localized in the diamond 2Fe‐2S core, there is the possibility for NO to be chemically excised in all three of its oxidation states: NO¯, NO·, and NO^+^. This report points to the fact that the characterization of such delocalized systems cannot predict polarization from exogenous agents or thermodynamic/kinetic control of products. Indeed, the delineating, intricate features for the needed control of NO storage and transfer in biological settings seem to require information derived from advanced synthetic targets, including mixed donor sites.^[^ ^63^, ^64^ ^]^

## Conflict of Interest

The authors declare no conflict of interest.

## Author Contributions

S.S. designed the project and carried out all experimental work, including the isolation and characterization of complexes, SC‐XRD data collection, simulation and plotting of Mössbauer spectra, and computational calculations. S.S. also organized the initial draft and revised subsequent versions. M.Q. recorded the EPR data. J.L., Y.H., F.Y., and J.G. conducted the XAS measurements. N.B. helping in solving the X‐ray Structures. P.A.L. performed and analyzed the Mössbauer measurements, reviewed the data, and contributed to the manuscript drafting. B.S.P. and P.B.B. provided guidance for the general understanding of the study. M.B.H. designed the computational approach, supervised the analysis and interpretation of results, and contributed to drafting the manuscript. M.Y.D. conceptualized the project, interpreted results, provided overall supervision, and reviewed, edited, and finalized the manuscript. All authors reviewed and approved the final version of the manuscript for submission.

## Supporting information



Supporting Information

## Data Availability

The data that support the findings of this study are available in the supplementary material of this article.
