# Supplementary material for: Structural and Electronic Complexities of a Sulfur‐Bridged Di‐Iron Complex Composed of Mono‐ and Di‐Nitrosyl Units
Source: Adv Sci (Weinh). 2025 Oct 27;13(1):e13976. doi: 10.1002/advs.202513976 (PMC12767009; doi:10.1002/advs.202513976)
Supplement: Supplementary file 1 — Supporting Information [file ADVS-13-e13976-s001.docx]

**Structural and Electronic Complexities of a Sulfur-Bridged Di-Iron Complex Composed of Mono- and Di-nitrosyl Units**

Sarnali Sanfui,^a^ Manuel Quiroz,^a^ Jialu Li,^b^ Yang Ha,^b^ Feipeng Yang,^c^ Jinghua Guo,^b^ Nattamai Bhuvanesh,^a^ Brad S. Pierce,^d^ Perla B. Balbuena,*^a,e^* Paul A. Lindahl,^a^ Michael B. Hall,^a^ and Marcetta Y. Darensbourg^*a^

*^a^Department of Chemistry, Texas A&M University, College Station, TX 77843, USA*

*^b^Advanced Light Source, Lawrence Berkeley National Laboratory, Berkeley, CA 94720, USA*

*^c^National Synchrotron Light Source II, Brookhaven National Laboratory, Upton, NY 11973, USA*

*^d^Department of Chemistry & Biochemistry, University of Alabama, Tuscaloosa, AL 35487, USA*

*^e^Department of Chemical Engineering; Department of Materials Science and Engineering, Texas A&M University, College Station, TX 77843, USA*

*Email:* [*marcetta@chem.tamu.edu*](mailto:marcetta@chem.tamu.edu)

**CONTENTS**

| 1. General Procedures  1.1. Methods and Materials  1.2. Instrumentation  1.3. X-ray Diffraction Analyses  1.4. Electrochemistry | S2  S2  S2  S2  S3 |
| --- | --- |
| 2. Experimental Procedures and Characterizations  2.1. Synthesis of [^L3^Fe]_2_  2.2. Synthesis of [^L3^Fe_2_(NO)_3_][BF_4_]  2.3. Synthesis of [^L3^Fe_2_(NO)_3_]^0^  2.4. Synthesis of [^L3^Fe_2_(NO)_3_][K(18-crown-6)]  2.5. Synthesis of [Fe(CO)_3_(^15^NO)][Na(18-crown-6)]  2.6. Preparation of [^15^NO][BF_4_]  2.7. Synthesis of [^L1^Fe_2_(^15^NO)_3_][BF_4_]  2.8. Synthesis of [^L2^Fe_2_(^15^NO)_3_][BF_4_]  2.9. Synthesis of [^L3^Fe_2_(^15^NO)_3_][BF_4_]  2.10. Synthesis of [^L1^Fe(^15^NO)·Fe(^14^NO)_2_][BF_4_] | S3  S3  S4  S4  S5  S5  S6  S6  S7  S7  S8 |
| 3. Structural comparison | S9 |
| 4. Electrochemical Studies | S11 |
| 5. Crystallographic characterization | S11 |
| 6. IR Spectroscopy | S22 |
| 7. ESI-Mass Spectrometry | S25 |
| 8. ^15^N NMR Spectroscopy | S28 |
| 9. Calculation of Magnetic Susceptibility using the Evans Method | S29 |
| 10. EPR Spectroscopy | S31 |
| 11. Mössbauer Spectroscopy. | S35 |
| 12. S K-edge XAS | S36 |
| 13. Computational Modelling | S36 |
| 14. References | S44 |

1. **General Procedures**
   1. **Methods and Materials.**

Solvents (CH_2_Cl_2_, CH_3_CN, Et_2_O, pentane, and hexane) used for the reaction were purified and dried by the Alcoa F200 activated alumina desiccant in the MBraun Manual Solvent Purification System. The manipulation and reactions were carried out in anaerobic conditions using a standard Schlenk or glove box technique under the N_2_ atmosphere. The following ligand and the compounds used in the present study were synthesized following the reported procedure: (L1 = *N,N’*-bis(2- mercaptoethyl)-1,5-diazacycloheptane),^1,2^ (L2 = *N,N’*-bis(2-mercaptoethyl) diazamethylethane),^4^ L3 (L3 = *N,N’*-bis(2-mercaptoethyl)-diazaphenylethane)^5^, **[^L1^Fe]_2_**^1,3^, **[^L2^Fe]_2_**^4^, **^L1^Fe(NO)**^6^, **^L2^Fe(NO)**^7^, **[Fe(CO)_3_(NO)][Na(18-crown-6)**^8^, **[^L1^Fe_2_(NO)_3_][BF_4_]**,^8^ **[^L2^Fe_2_(NO)_3_][BF_4_]**^9^. The reagent grade [NO][BF_4_], [n-Bu_4_N][PF_6_], KHBEt_3_ (1 M, THF), and OEP (octaethylporphyrin) were purchased from Sigma-Aldrich.

- 1. **Instrumentation.**

The solution phase infrared (IR) spectra were recorded in the Bruker Tensor 37 Fourier transform IR (FTIR) using the CaF2 cell having a 0.2 mm path length. The ESI-mass spectra of the complexes were recorded in the laboratory for Biological Mass Spectrometry at Texas A&M University. ^1^H, ^15^N, and ^19^F NMR spectra were recorded on the Inova 400 MHz superconducting NMR instrument. The X-band EPR spectra were collected on a Bruker Elexsys E500 spectrometer, and Mössbauer spectra were collected on an MS4 WRC spectrometer (SEE Co) at 5 to 6 K and 0.05 T, with the magnetic field applied parallel to the radiation. An a-iron foil was used for RT calibration. MB simulations were obtained using WMOSS software. The mathematical model was simulated using Wolfram Mathematica.

- 1. **X-ray Diffraction Analyses.**

Single-crystal X-ray diffraction data for **[^L3^Fe]_2_**, **[^L3^Fe_2_(NO)_3_][BF_4_]**, **[^L3^Fe_2_(NO)_3_]**, and **[^L3^Fe_2_(NO)_3_][K(18-crown-6]** were collected at 100 K on the BRUKER APEX 3 X-ray (three-circle) diffractometer with Mo sealed X-ray tube (K_α_ = 0.70173Å). Single-crystal X-ray diffraction data for the complexes were collected at 100 K on the BRUKER Venture X-ray (kappa geometry) diffractometer with Cu-Iμs X-ray tube (K_α_ = 1.5418Å). The data integration and reduction were processed with SAINT software.^10^ An absorption correction was applied using the SADABS program.^11^ Hydrogen atoms were placed at idealized positions and were refined by fixed isotropic displacement parameters. Anisotropic displacement parameters were employed for all non-hydrogen atoms. All structures were solved and refined by the direct method using SHELXS/XT and were refined on *F*^2^ by the full-matrix least-squares technique.^11^ The final pictorial presentation of structures was generated in Olex2.^12^ Crystallographic data are deposited in the Cambridge Crystallographic Data Centre for the complexes **[^L3^Fe]_2_** (CCDC 2374364), **[^L3^Fe_2_(NO)_3_].[BF_4_]** (CCDC 2322823), **[^L3^Fe_2_(NO)_3_]** (CCDC 2326021), **[^L3^Fe_2_(NO)_3_][K(18-crown-6]** (CCDC 2374365).

- 1. **Electrochemistry.**

Cyclic voltammograms were recorded on HCH instruments, Inc., using a CHI600E electrochemical analyzer in dichloromethane or/and acetonitrile with 0.1 M tetrabutylammonium hexafluorophosphate (TBAH) as a supporting electrolyte at room temperature under the Ar atmosphere. An electrochemical cell fitted with three electrodes was used for the cyclic voltammetric study. These three electrodes are the reference electrode (a CH_3_CN solution of Ag/AgNO_3_ in a Vycor-tipped glass tube), the auxiliary electrode (platinum wire), and the working electrode (0.071 cm^2^ glassy carbon electrode). The concentration of the complex was of the order of 10^−3^ M. Potentials for all the complexes were given relative to the Fc/Fc^+^ couple at 0.00 V.

1. **Experimental Procedures and Characterizations**

**2.1. Synthesis of [^L3^Fe]_2_:**

In a 50 mL round bottom flask, *N,N’*-bis(2- mercaptoethyl)-1,5-diazabenzylethane (bme-dabz, **L3**) ligand (1.08 g, 0.003 mol) was taken and dissolved in 10 mL of toluene. A toluene solution (10 mL) of iron(III) acetylacetonate (0.530 g, 0.0015 mol) was added to the toluene solution of the **L3** ligand. Then the reaction was allowed to stir overnight at room temperature under a N_2_ atmosphere. With time, the light orange precipitation developed in the solution. The resulting light orange powder was collected on the Buchner funnel under a N_2_ atmosphere and washed with a copious amount of toluene. Yield: 0.89 g (73 %). The CH_2_Cl_2_ solution of **[^L3^Fe]_2_** was layered with cold ether to yield orange X-ray quality crystals.

**2.2. Synthesis of** **[^L3^Fe_2_(NO)_3_][BF_4_]:**

In a 40 mL glass vial, 0.083 g (0.10 mmol) of **[^L3^Fe]_2_**, 0.092 g (0.20 mmol) of [Fe(CO)_3_NO][Na(18-crown-6)], and 0.047 g (0.40 mmol) of [NO][BF_4_] were taken and then the contents were dissolved in 20 mL of freshly distilled dry CH_2_Cl_2_. The reaction was allowed to stir overnight at room temperature, and completion of the reaction was monitored through IR spectroscopy. The product was precipitated out by adding 15 mL of diethyl ether and then washed 3 times with 15 mL of diethyl ether. The solid product was redissolved with 10 mL of CH_2_Cl_2_ and filtered through a small pad (2 cm) of celite. The CH_2_Cl_2_ solution of the product was layered with cold hexane to yield reddish brown X-ray quality crystals of **[^L3^Fe_2_(NO)_3_]^+­^**. Isolated yield: 0.054 g (80 %). IR (THF) ν(NO): 1809, 1779, 1742 cm^−1^. Elem. Anal. Calcd for C_20_H_26_BF_4_Fe_2_N_5_O_3_S_2_: C, 37.12; H, 4.05; N, 10.82. Found: C, 37.08; H, 4.06; N, 10.77.

**2.3. Synthesis of [^L3^Fe_2_(NO)_3_]^0^:**

In a 40 mL glass vial, 0.068 g (0.10 mmol) of **[^L3^Fe_2_(NO)_3_]^+^** was taken and dissolved with 10 mL of freshly distilled dry THF. The solution was cooled down to −10 ^o^C. Then 0.1 mL (0.1 mmol) of 1 M (THF) KHBEt_3_ or KC_8_ (0.014 mg, 0.1 mmol) was added and stirred for 10 min at 0 ^o^C. The reaction mixture turned a black forest green color. The product was precipitated out by adding 20 mL of diethyl ether and then washed 2 times with 15 mL of cold diethyl ether. The solids were dissolved in cold CH_2_Cl_2_ and filtered through a small pad of celite (2 cm). Layering the CH_2_Cl_2_ solution of the product with hexane yielded black forest green X-ray quality crystals of **[^L3^Fe_2_(NO)_3_]^0^** at −35 ^o^C. Isolated yield: 0.034 g (61%). IR (THF) ν(NO): 1701, 1667, 1640 cm^−1^.

**2.4. Synthesis of [^L3^Fe_2_(NO)_3_][K(18-crown-6)]:**

The product **[^L3^Fe_2_(NO)_3_]^−^** was synthesized in a similar manner as 0.068 g (0.10 mmol) of **[^L3^Fe_2_(NO)_3_]^+^** was sequentially reduced with 0.1 mL (0.1 mmol) of 1 M (THF) KHBEt_3_ or KC_8_ (0.014 mg, 0.10 mmol) to obtain **[^L3^Fe_2_(NO)_3_]^0^** at −10 ^o^C. A second equivalent addition of 1 M (THF) KHBEt_3_ (0.13 mL, 0.13 mmol) or KC_8_ (0.014 g, 0.10 mmol) and 18-crown-6 (0.026 g, 0.10 mmol) to the in situ generated **[^L3^Fe_2_(NO)_3_]^0^** was done at −35 ^o^C. The completion of the reaction was monitored using IR spectroscopy. The product was precipitated out by adding 20 mL of diethyl ether and then washed 2 times with 15 mL of cold diethyl ether. The THF solution was filtered over a small pad (2 cm) of celite. Layering the THF solution of the product with cold hexane produced dark-orange X-ray quality crystals of **[^L3^Fe_2_(NO)_3_]^−^** at −35 ^o^C. Isolated yield: 0.046 g (50 %). IR (THF) ν(NO): 1655, 1624, 1607 cm^−1^.

**2.5. Synthesis of [Fe(CO)_3_(^15^NO)][Na(18-crown-6)]:**

In a 100 mL Schlenk flask, 1.26 g (0.0034 mol) of [Na^+^(18-crown-6)]^15^NO_2_^−^ was taken, and 20 mL of THF was added to it. Then it was cooled down to −10 ^o^C under a N_2_ atmosphere, and 1.12 g (0.0057 mol) of iron pentacarbonyl was added dropwise by cannula to the Schlenk flask. The reaction mixture was stirred for an hour and then filtered anaerobically through a celite plug. Concentrate the solution by evaporation anaerobically, followed by the addition of diethyl ether, precipitated out the amber-colored powder, and then it was washed three times with diethyl ether and dried under vacuum. Yield: 0.087 g (56 %). IR (CH_3_CN) ν(NO): 1615 cm^−1^, ν(CO): 1885 and 1987 cm^−1^.

**2.6. Preparation of [^15^NO][BF_4_]:**

The preparation of [^15^NO][BF_4_] was done following the modification of a reported literature procedure.^13^ In a 20 mL vial, 0.25 mL of H_2_O and 2.5 mL (13.9 mmol) of *n*-pentanol were taken and cooled the mixture to 0 ^o^C. Then 0.35 mL of conc. H_2_SO_4_ was added dropwise to the mixture and stirred for 5 min. The mixture was then cooled to −10 ^o^C, and a cooled (0 ^o^C) aqueous solution (4 mL) of Na[^15^NO_2_] (0.95 g, 13.8 mmol) was added dropwise, and the mixture to stirred for another 3-4 h. The upper pale-yellow layer of crude *n*-pentyl nitrite was decanted off from precipitated Na_2_SO_4_ and dried over anhydrous MgSO_4_ for 1 h. Then the crude *n*-pentyl nitrite was used for the next step. A 20 mL DCM solution containing 0.25 mL (1.86 mmol) of *n*-pentyl nitrite was cooled to −10 °C, and 0.5 mL (1.86 mmol) of ~55 % w/w HBF_4_•Et_2_O was added dropwise. The reaction mixture was stirred for 30 min at −10 °C. The white solid product was collected on a fritted glass Buchner filtering funnel and was washed with copious amounts of cold DCM. The product was transferred to a vial and dried under vacuum. Yield: 0.175 g (80%).

**2.7. Synthesis of [^L1^Fe_2_(^15^NO)_3_][BF_4_]:**

In a 40 mL glass vial, 0.055 g (0.10 mmol) of **[^L1^Fe]_2_**, 0.092 g (0.20 mmol) of [Fe(CO)_3_(^15^NO)][Na(18-crown-6)], and 0.047 g (0.40 mmol) of [^15^NO][BF_4_] were taken and then the contents were dissolved in 20 mL of freshly distilled dry CH_2_Cl_2_. The reaction was allowed to stir overnight at room temperature, and the completion of the reaction was monitored through IR spectroscopy. The product was precipitated out by adding 15 mL of diethyl ether and then washed 3 times with 15 mL of diethyl ether. The solid product was redissolved with 10 mL of CH_2_Cl_2_, filtered through a small pad (2 cm) of celite, and layered with cold hexane to yield a dark red crystalline product of **[^L1^Fe_2_(NO)_3_]^+^**. Isolated yield: 0.042 g (76 %). IR (CH_3_CN) ν(NO): 1765, 1734, 1709 cm^−1^. ^15^N NMR (CD_3_CN, 400 MHz): δ 390.77, 414.42, 435.06 ppm.

**2.8. Synthesis of [^L2^Fe_2_(^15^NO)_3_][BF_4_]:**

In a 40 mL glass vial, 0.052 g (0.10 mmol) of **(^L2^Fe)_2_**, 0.092 g (0.20 mmol) of [Fe(CO)_3_(^15^NO)][Na(18-crown-6)], and 0.047 g (0.40 mmol) of [^15^NO][BF_4_] were taken and then the contents were dissolved in 20 mL of freshly distilled dry CH_2_Cl_2_. The reaction was allowed to stir overnight at room temperature, and completion of the reaction was monitored through IR spectroscopy. The product was precipitated out by adding 15 mL of diethyl ether and then washed 3 times with 15 mL of diethyl ether. The solid product was redissolved with 10 mL of CH_2_Cl_2_, filtered through a small pad (2 cm) of celite, and layered with cold hexane to yield a dark red crystalline product of **[^L2^Fe_2_(^15^NO)_3_]^+^**. Isolated yield: 0.042 g (78 %). IR (CH_3_CN) ν(NO): 1767, 1740, 1707 cm^−1^. ^15^N NMR (CD_3_CN, 400 MHz): δ 392.91, 423.25, 448.44 ppm.

**2.9. Synthesis of [^L3^Fe_2_(^15^NO)_3_][BF_4_]:**

In a 40 mL glass vial, 0.083 g (0.10 mmol) of **[^L3^Fe]_2_**, 0.092 g (0.20 mmol) of [Fe(CO)_3_(^15^NO)][Na(18-crown-6)], and 0.047 g (0.40 mmol) of [^15^NO][BF_4_] were taken and then the contents were dissolved in 20 mL of freshly distilled dry CH_2_Cl_2_. The reaction was allowed to stir overnight at room temperature, and completion of the reaction was monitored through IR spectroscopy. The product was precipitated out by adding 15 mL of diethyl ether and then washed 3 times with 15 mL of diethyl ether. The solid product was redissolved with 10 mL of CH_2_Cl_2_, filtered through a small pad (2 cm) of celite, and layered with cold hexane to yield a dark red crystalline product of **[^L3^Fe_2_(^15^NO)_3_]^+^**. Isolated yield: 0.055 g (79 %). IR (CH_3_CN) ν(NO): 1768, 1740, 1707 cm^−1^. ^15^N NMR (CD_3_CN, 400 MHz): δ 398.45, 423.50, 448.22 ppm.

**2.10. Synthesis of [^L1^Fe(^15^NO)**·**Fe(^14^NO)_2_][BF_4_]:**

In a 50 mL Schlenk flask, 0.031 g (0.10 mmol) of **^L1^Fe(^15^NO)** was taken and dissolved in dichloromethane, and then the dichloromethane solution of 0.077 g (0.10 mmol) of [Fe(PPh_3_)_2_(NO)_2_][BF_4_] was added by cannula, maintaining the reaction temperature at −10 ^o^C. Then the reaction was allowed to stir for 30 min at −10 ^o^C and the completion of the reaction was monitored through IR spectroscopy. The product was precipitated out by adding diethyl ether and then washed 3 times with 20 mL of diethyl ether. The solid product was redissolved with 10 mL of CH_2_Cl_2_, filtered through a small pad (2 cm) of celite, and layered with cold hexane to yield a dark red crystalline product of **[^L1^Fe(^15^NO)⸳Fe(^14^NO)_2_]^+^** at −30 ^o^C. Isolated yield: 0.039 g (70 %). IR (CH_2_Cl_2_) ν(NO): 1760 and 1711 cm^−1^. ESI-MS: *m/z* 420.9520 ([M]^+^). ^15^N NMR (CD_3_CN, 400 MHz): δ 390.02 ppm.

**Scheme S1.** Synthetic outline of **[^L^Fe_2_(NO)_3_]^+^** following routes A), B), C), and D) for designated ligands **L1**, **L2**, and **L3**.

1. **Structural comparison Between Three Ligand Systems:**

*N,N’*-bis(2-mercaptoethyl)-1,5-diazacycloheptane (**L1**) is a closed chain N_2_S_2_ ligand whereas *N,N’*-bis(2-mercaptoethyl) diazamethylethane (**L2**) and *N,N’*-bis(2-mercaptoethyl)-diazaphenylethane (**L3**) are open chain N_2_S_2_ ligand systems. Structural and bonding properties largely depend on the ligand’s rigidity. Here, the structural differences of iron complexes comprised of **L1**, **L2**, and **L3** ligand systems are shown in Table S1 to highlight the differences between the three systems. The major noticeable changes were observed in the bond angles ∠S-Fe-S and ∠N-Fe-N. When the ligand is a closed-chain rigid system (**L1**), the ∠S-Fe-S angle in **[^L1^Fe]_2_** is 108.38°, which is large compared to open-chain systems **[^L2^Fe]_2_** (97.01°) and **[^L3^Fe]_2_** (97.42°). In addition, the ∠N-Fe-N angle for the closed-chain system, **[^L1^Fe]_2_** is smaller (72°) compared to the open-chain systems (**[^L2^Fe]_2_** and **[^L3^Fe]_2_**). The distance between the iron and the bridging sulfur in closed system is larger than in open chain systems. However, the other bond parameters, such as Fe-N, Fe-S, etc., are very similar (Table S1). Depending on the bite angle, that is, the ∠S-Fe-S angle here, MN_2_S_2_ binds differently with the exogenous metal synthons.^2,14^


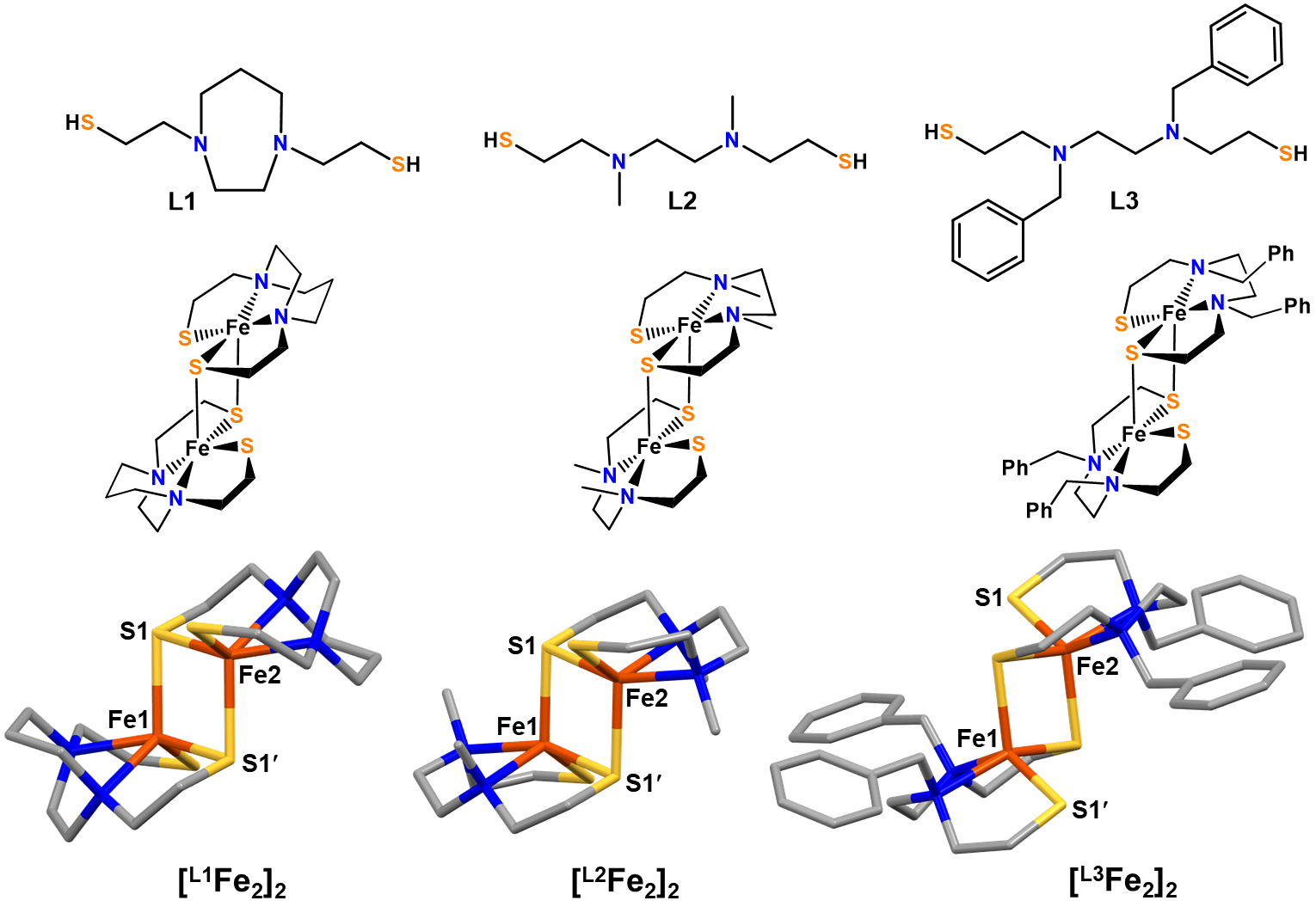


**Figure S1.** ChemDraw structures of ligands and the corresponding iron complexes along their SC-XRD structures; color codes: C: grey, S: yellow, Fe: orange; hydrogens are omitted for clarity.

**Table S1.** Structural parameters of iron dimers

|  | **[^L1^Fe]_2_** | **[^L2^Fe]_2_** | **[^L3^Fe]_2_** |
| --- | --- | --- | --- |
| Fe···Fe/Å | 3.174 | 3.205 | 3.130 |
| Fe-N_av_/Å | 2.265 | 2.257 | 2.264 |
| Fe-S_api_/Å | 2.327 | 2.304 | 2.323 |
| Fe-S′_api_(Fe)/Å | 2.443 | 2.471 | 2.447 |
| Fe-S_brid_(Fe)/Å | 2.410 | 2.379 | 2.385 |
| ∠S-Fe-S/° | 108.38 | 97.01 | 97.42 |
| ∠N-Fe-N/° | 72 | 79.28 | 79.86 |
| τ_5_ | 0.47 | 0.44 | 0.33 |
| *^a^*Δ^Fe^/Å | 0.68 | 0.67 | 0.66 |
| *^b^*Ref. | 15 | 16 | Tw |

*^a^*Fe displacement from N_2_S_2_ plane; *^b^*Reference; Tw: This work.

1. **Electrochemical Studies**.


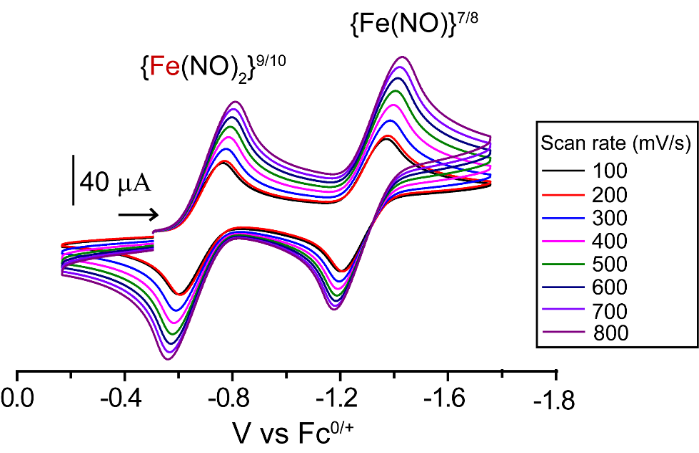


**Figure S2.** The cyclic voltammograms of **[^L3^Fe_2_(NO)_3_]^+^** as a BF_4_ salt, in a 2 mM CH_2_Cl_2_ solution, showing two one-electron reversible events at −0.68 and −1.26 V, assigned for {Fe(NO)_2_}^9/10^ and {Fe(NO)}^7/8^ couples, respectively, vs. Fc^0/+^, at different scan rates of 100 - 800 mV/s, using glassy carbon as working electrode containing 0.1 M of [^t^Bu_4_N][PF_6_] as supporting electrolyte.

1. **Crystallographic Characterization**.

Iron dimer **[^L3^Fe]_2_** and diiron trinitrosyl complexes **[^L3^Fe_2_(NO)_3_]^+^**, **[^L3^Fe_2_(NO)_3_]^0^**, and **[^L3^Fe_2_(NO)_3_]^−^** are characterized by X-ray crystallography. Complexes **[^L3^Fe]_2_** and **[^L3^Fe_2_(NO)_3_]^+^** have been crystallized from dichloromethane solutions of the respective complexes upon layering with diethyl ether after 3-5 days. Layering a THF solution of **[^L3^Fe_2_(NO)_3_]^0^** with cold *n*-hexane produced dark-orange X-ray quality crystals at −35 ^o^C, and the CH_2_Cl_2_ solution of **[^L3^Fe_2_(NO)_3_]^−^** layered with *n*-hexane yielded black forest green X-ray quality crystals at −35 ^o^C. The X-ray crystallographic analysis unambiguously elucidated the structure of **[^L3^Fe]_2_**, **[^L3^Fe_2_(NO)_3_]^+^**, **[^L3^Fe_2_(NO)_3_]^0^**, and **[^L3^Fe_2_(NO)_3_]^−^**.

The molecular structure and the molecular packing diagrams for complexes are shown in Figures S3-S10. Crystallographic parameters of the complexes are given in Tables S2-S6, and selected structural parameters of the complexes are tabulated in Table S6. The Fe1···Fe2 distance in **[^L3^Fe_2_(NO)_3_]^+^** is 2.7794(18) Å, consistent with the strong antiferromagnetic, i.e., metal-metal bonding interaction, described earlier. The displacement of Fe1 from the best N_2_S_2_ plane is 0.05 Å, and the Fe1-N-O is 162.6^o^ in the **[^L3^Fe_2_(NO)_3_]^+^**. The addition of one electron produces a singly reduced species, **[^L3^Fe_2_(NO)_3_]^0^**, where the Fe1···Fe2 distance elongates to 2.912 Å, suggesting almost no metal-metal interaction is present. The displacement of Fe1 from the best N_2_S_2_ plane is similar to **[^L3^Fe_2_(NO)_3_]^+^** (0.053 Å) but the $\boldsymbol{\angle}$Fe1-N-O angle in **[^L3^Fe_2_(NO)_3_]^0^** is decreased by ~10 ^o^ compared to **[^L3^Fe_2_(NO)_3_]^+^**. In the case of the two-electron reduced species, the structural parameters are changed significantly compared to the other two congeners. The Fe1 displacement from the best N_2_S_2_ plane is here 0.85 Å, analogous to the large displacements observed in the other examples of **[^L^Fe_2_(NO)_3_]^−^** anions. As a result, the Fe1···Fe2 distance is enlarged (3.138 Å) and the $\boldsymbol{\angle}$Fe1-N-O angle changes towards linearity, 166.4 ^o^, in **[^L3^Fe_2_(NO)_3_]^−^** compared to **[^L3^Fe_2_(NO)_3_]^0^**.


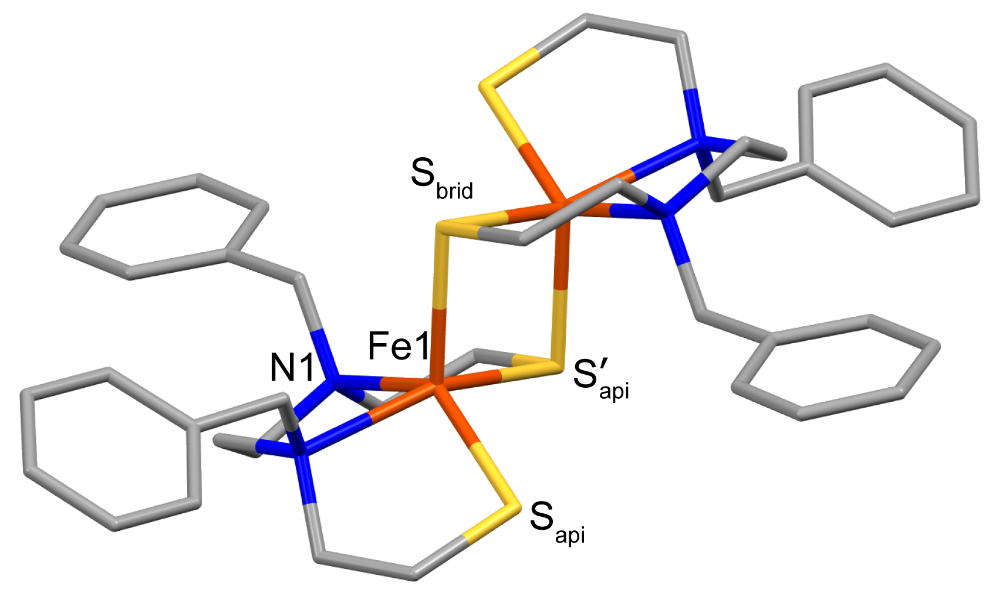


**Figure S3.** Molecular structure of **[^L3^Fe]_2_** (H atoms have been omitted for clarity).


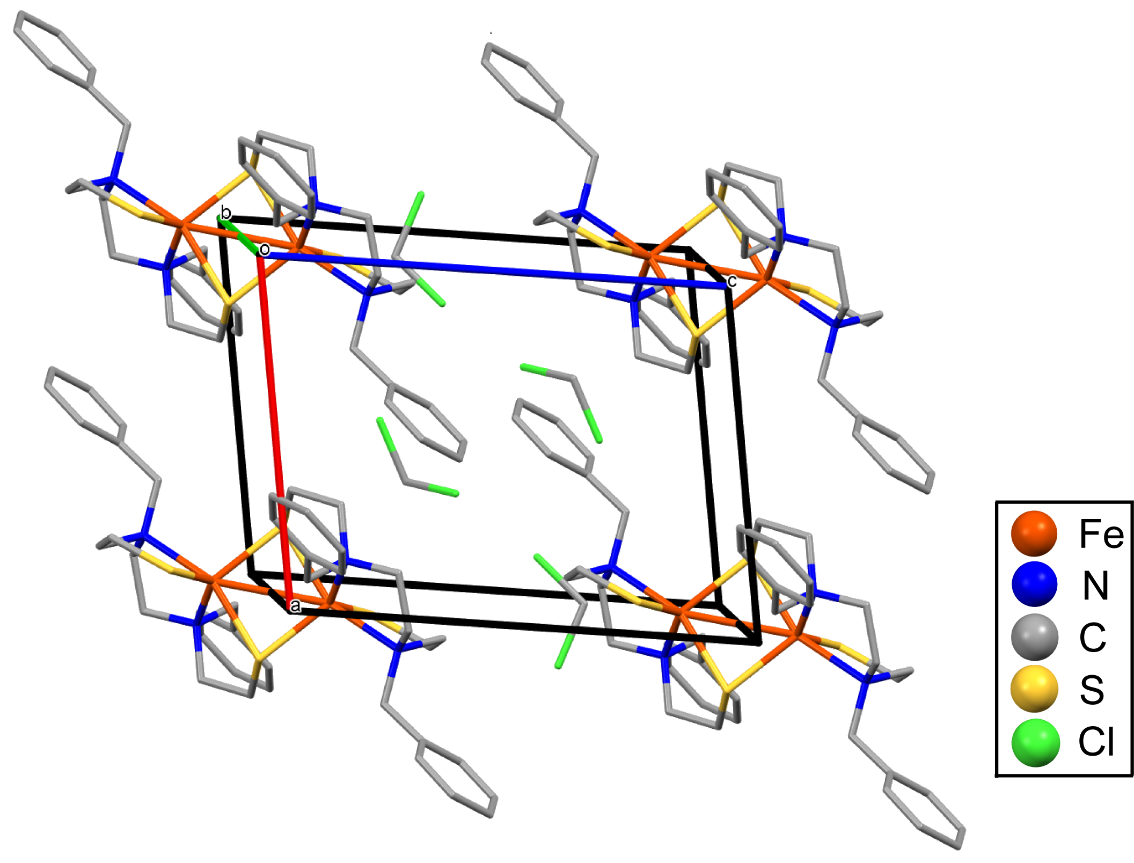


**Figure S4.** Molecular packing diagram of **[^L3^Fe]_2_** (H atoms have been omitted for clarity).


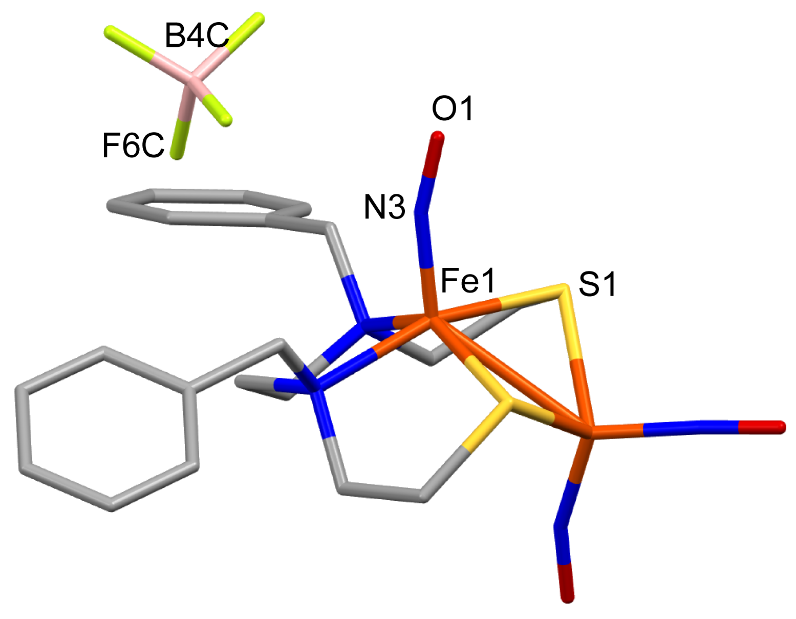


**Figure S5.** Molecular structure of **[^L3^Fe_2_(NO)_3_]^+^** (hydrogen atoms have been omitted for clarity).


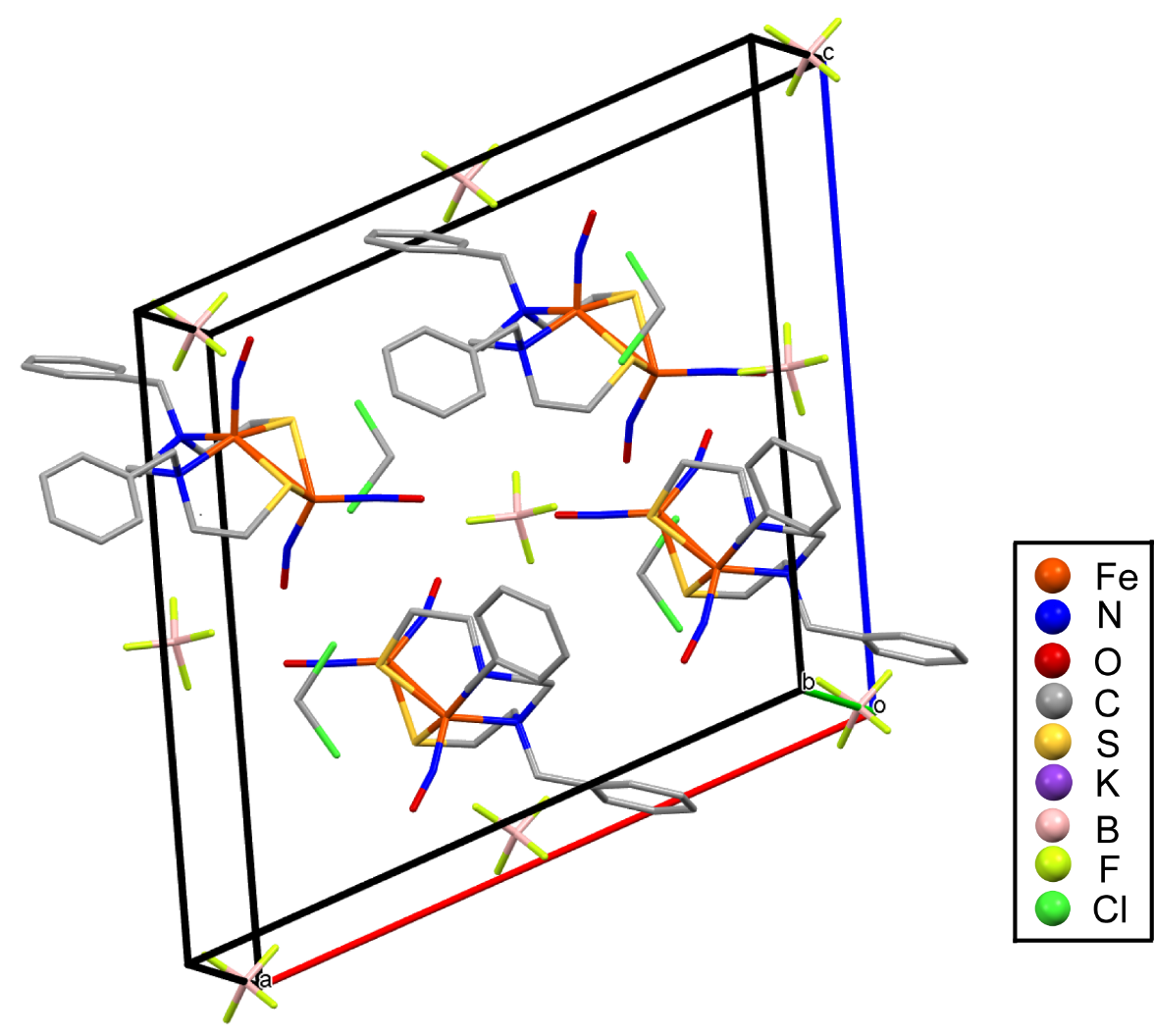


**Figure S6.** Molecular packing diagram of **[^L3^Fe_2_(NO)_3_]^+^** (hydrogen atoms have been omitted for clarity).


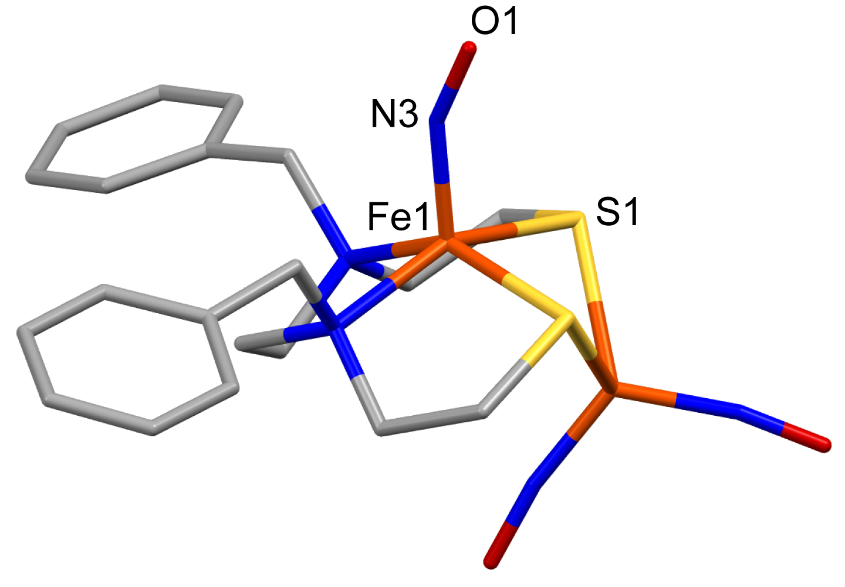


**Figure S7.** Molecular structure of **[^L3^Fe_2_(NO)_3_]^0^** (hydrogen atoms have been omitted for clarity).


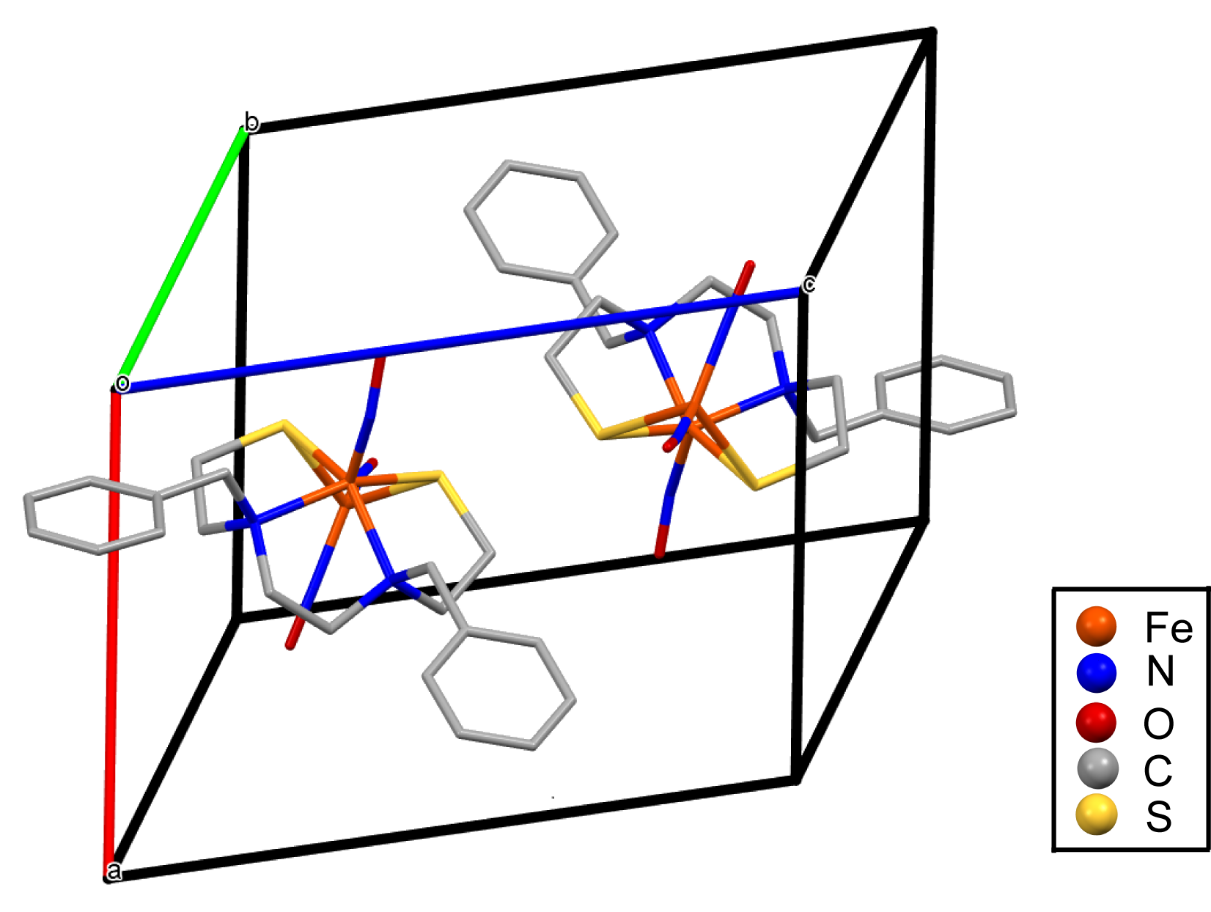


**Figure S8.** Molecular packing diagram of **[^L3^Fe_2_(NO)_3_]^0^** (hydrogen atoms have been omitted for clarity).


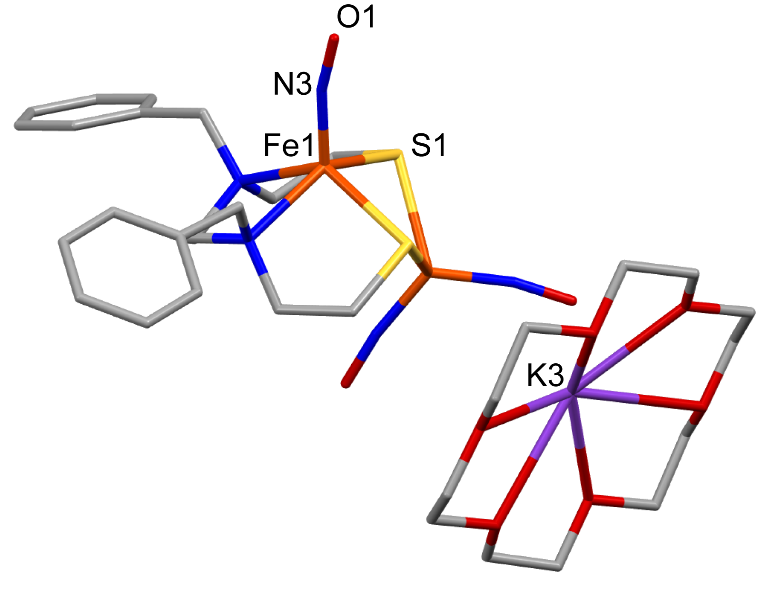


**Figure S9.** Molecular structure of **[^L3^Fe_2_(NO)_3_]^−^** (hydrogen atoms have been omitted for clarity).


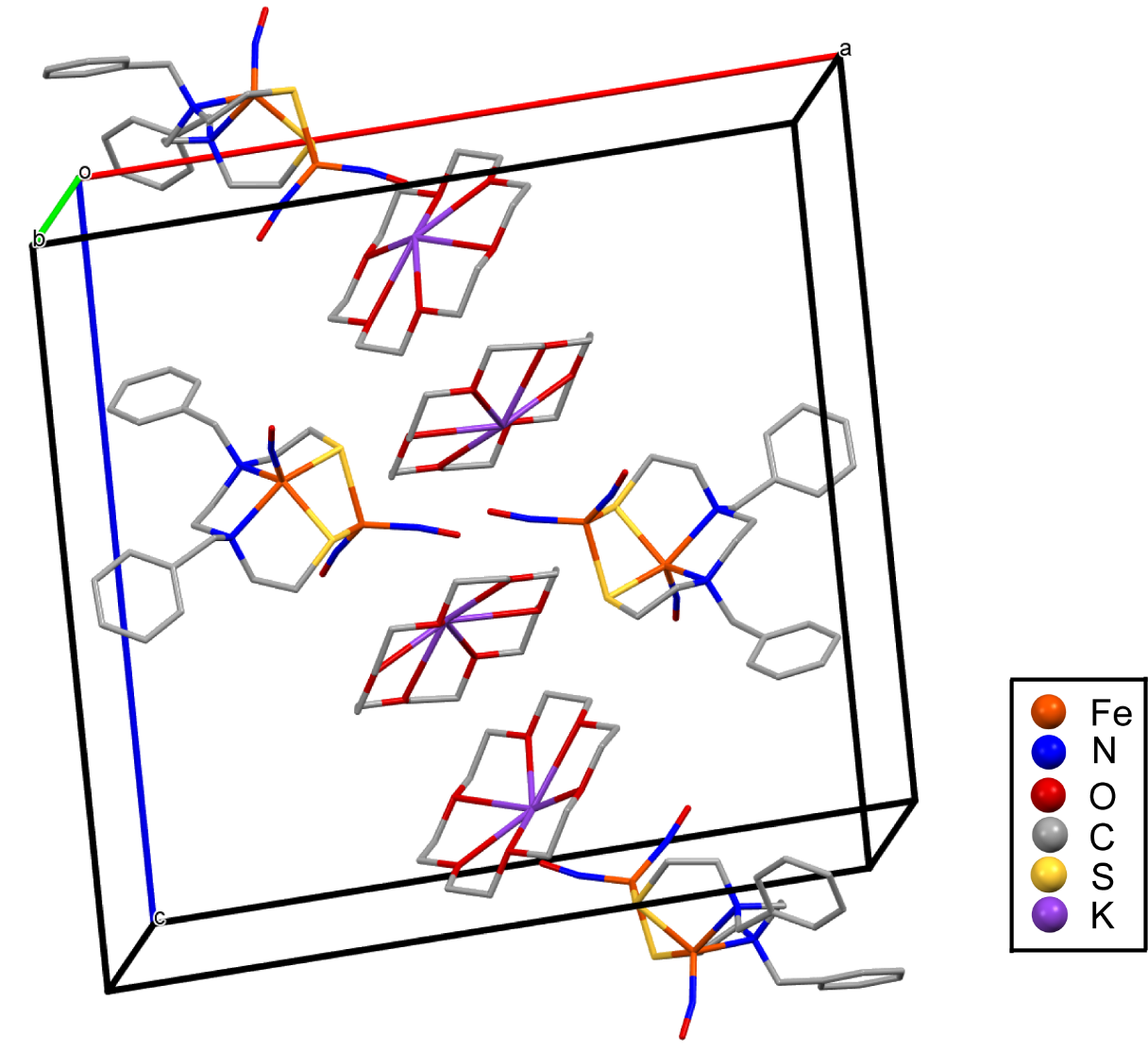


**Figure S10.** Molecular packing diagram of **[^L3^Fe_2_(NO)_3_]^−^** (hydrogen atoms have been omitted for clarity).

**Table S2.** Crystal data and structure refinement for **[^L3^Fe]_2_** (CCDC 2374364).

| Empirical formula | C_44_H_60_Cl_8_Fe_2_N_4_S_4_ | |
| --- | --- | --- |
| Formula weight | 1168.50 | |
| Temperature | 110 K | |
| Crystal system | Triclinic | |
| Space group | *P*-1 | |
| Unit cell dimensions | *a* = 9.4912(5) Å | *α* = 83.248(3)^o^ |
|  | *b* = 11.2950(6) Å | *β* = 79.447(2)^o^ |
|  | *c* = 12.5752(7) Å | *γ* = 85.726(3)^o^ |
| Volume | 1314.17(12) Å^3^ | |
| *Z* | 1 | |
| Radiation | Mo Kα (λ = 0.71073 Å) | |
| Density (calculated) | 1.476 g cm^−3^ | |
| Absorption coefficient (*μ*) | 9.929 mm^−1^ | |
| F(000) | 604 | |
| Crystal size | 0.229 x 0.208 x 0.15 mm^3^ | |
| Theta range for data collection | 3.595 to 70.391^o^ | |
| Index ranges | −11<=h<=11, −13<=k<=13, −14<=l<=15 | |
| Reflections collected | 34866 | |
| Independent reflections | 5016 [R(int) = 0.0586] | |
| Completeness to theta = 67.679 ^o^ | 99.8 % | |
| Refinement method | Full-matrix least-squares on *F*^2^ | |
| Data/restraints/parameters | 5016 / 22 / 304 | |
| Goodness-of-fit on *F*^2^ (GooF) | 0.0586 | |
| Final R indices [I>2σ(I)] | *R_1_* = 0.0510, *wR_2_* = 0.1346 | |
| R indices (all data) | *R_1_* = 0.0544, *wR_2_* = 0.1380 | |
| Largest diff. peak and hole | 0.912 and −0.658 e. Å^−3^ | |

^a^R_1_=Σ(||*F_o_*|-|*F_c_*||)/Σ|*F_o_*|. ^b^wR_2_=[Σ[*w*(*F_o_*^2^-*F_c_*^2^ )^2^]/Σ[*w*(*F_o_*^2^)^2^]]^1/2^, *w*=1/[σ^2^(*F_o_*^2^)+(*ap*)^2^+*bp*], where *p*=[max(*F_o_*^2^ , 0)+2*F_c_*^2^]/3.

**Table S3.** Crystal data and structure refinement for **[^L3^Fe_2_(NO)_3_]^+^** (CCDC 2322823).

| Empirical formula | C_21_H_28_BCl_2_F_4_Fe_2_N_5_O_3_S_2_ | |
| --- | --- | --- |
| Formula weight | 732.01 | |
| Temperature | 110 K | |
| Crystal system | Monoclinic | |
| Space group | *C*2 | |
| Unit cell dimensions | *a* = 17.778(3) Å | *α* = 90^o^ |
|  | *b* = 9.7895(13) Å | *β* = 108.671(4)^o^ |
|  | *c* = 17.156(2) Å | *γ* = 90^o^ |
| Volume | 2828.7(7) Å^3^ | |
| *Z* | 4 | |
| Radiation | Mo Kα (λ = 0.71073 Å) | |
| Density (calculated) | 1.719 g cm^−3^ | |
| Absorption coefficient (*μ*) | 1.424 mm^−1^ | |
| F(000) | 1488 | |
| Crystal size | 0.117 x 0.078 x 0.028 mm^3^ | |
| Theta range for data collection | 2.341 to 22.428 ^o^ | |
| Index ranges | −19<=h<=19, −10<=k<=10, −18<=l<=18 | |
| Reflections collected | 16869 | |
| Independent reflections | 3694 [R(int) = 0.0987] | |
| Completeness to theta = 22.498 ^o^ | 99.7 % | |
| Refinement method | Full-matrix least-squares on *F*^2^ | |
| Data/restraints/parameters | 3694 / 1 / 363 | |
| Goodness-of-fit on *F*^2^ (GooF) | 1.081 | |
| Final R indices [I>2σ(I)] | *^a^R_1_* = 0.0435, *^b^wR_2_* = 0.0667 | |
| R indices (all data) | *^a^R_1_* = 0.0595, *^b^wR_2_* = 0.0729 | |
| Largest diff. peak and hole | 0.442 and −0.429 e. Å^−3^ | |

^a^R_1_=Σ(||*F_o_*|-|*F_c_*||)/Σ|*F_o_*|. ^b^wR_2_=[Σ[*w*(*F_o_*^2^-*F_c_*^2^ )^2^]/Σ[*w*(*F_o_*^2^)^2^]]^1/2^, *w*=1/[σ^2^(*F_o_*^2^)+(*ap*)^2^+*bp*], where *p*=[max(*F_o_*^2^ , 0)+2*F_c_*^2^]/3.

**Table S4.** Crystal data and structure refinement for **[^L3^Fe_2_(NO)_3_]^0^** (CCDC 2326021).

| Empirical formula | C_20_H_26_Fe_2_N_5_O_3_S_2_ | |
| --- | --- | --- |
| Formula weight | 560.28 | |
| Temperature | 110 K | |
| Crystal system | Triclinic | |
| Space group | *P*-1 | |
| Unit cell dimensions | *a* = 9.1698(8) Å | *α* = 93.185(2)^o^ |
|  | *b* = 10.2663(9) Å | *β* = 99.444(2)^o^ |
|  | *c* = 14.1459(13) Å | *γ* = 115.774(2)^o^ |
| Volume | 1170.82(18) Å^3^ | |
| *Z* | 2 | |
| Radiation | Mo Kα (λ = 0.71073 Å) | |
| Density (calculated) | 1.589 g cm^−3^ | |
| Absorption coefficient (*μ*) | 1.450 mm^−1^ | |
| F(000) | 1488 | |
| Crystal size | 0.24 × 0.14 × 0.04 mm^3^ | |
| Theta range for data collection | 2.642 to 29.642^o^ | |
| Index ranges | −12<=h<=12, −14<=k<=14, −19<=l<=19 | |
| Reflections collected | 32215 | |
| Independent reflections | 6459 [R(int) = 0.0229] | |
| Completeness to theta = 29.642^o^ | 96.50 % | |
| Refinement method | Full-matrix least-squares on *F*^2^ | |
| Data/restraints/parameters | 6459 / 0 / 289 | |
| Goodness-of-fit on *F*^2^ (GooF) | 1.037 | |
| Final R indices [I>2σ(I)] | *R_1_* = 0.0366, *wR_2_* = 0.0848 | |
| R indices (all data) | *R_1_* = 0.0412, *wR_2_* = 0.0890 | |
| Largest diff. peak and hole | 1.144 and −0.708 e. Å^−3^ | |

^a^R_1_=Σ(||*F_o_*|-|*F_c_*||)/Σ|*F_o_*|. ^b^wR_2_=[Σ[*w*(*F_o_*^2^-*F_c_*^2^ )^2^]/Σ[*w*(*F_o_*^2^)^2^]]^1/2^, *w*=1/[σ^2^(*F_o_*^2^)+(*ap*)^2^+*bp*], where *p*=[max(*F_o_*^2^ , 0)+2*F_c_*^2^]/3.

**Table S5.** Crystal data and structure refinement for **[^L3^Fe_2_(NO)_3_]^−^** (CCDC 2374365).

| Empirical formula | C_35.90_H_58.87_C_l1.30_Fe_2_KN_5_O_9_S_2_ | |
| --- | --- | --- |
| Formula weight | 965.55 | |
| Temperature | 100 K | |
| Crystal system | Monoclinic | |
| Space group | *P*21/c | |
| Unit cell dimensions | *a* = 23.2994(7) Å | *α* = 90^o^ |
|  | *b* = 8.3294(2) Å | *β* = 95.9020(10)^o^ |
|  | *c* = 22.7333(7) Å | *γ* = 90^o^ |
| Volume | 4388.5(2) Å^3^ | |
| *Z* | 4 | |
| Radiation | Mo Kα (λ = 0.71073 Å) | |
| Density (calculated) | 1.461 g cm^−3^ | |
| Absorption coefficient (*μ*) | 8.232 mm^−1^ | |
| F(000) | 2025 | |
| Crystal size | 0.247 x 0.061 x 0.023 mm^3^ | |
| Theta range for data collection | 3.814 to 70.180^o^ | |
| Index ranges | −28<=h<=28, −10<=k<=9, −27<=l<=27 | |
| Reflections collected | 60296 | |
| Independent reflections | 8310 [R(int) = 0.0744] | |
| Completeness to theta = 67.679 ^o^ | 99.7 % | |
| Refinement method | Full-matrix least-squares on *F*^2^ | |
| Data/restraints/parameters | 8310 / 582 / 521 | |
| Goodness-of-fit on *F*^2^ (GooF) | 1.061 | |
| Final R indices [I>2σ(I)] | *R_1_* = 0.0680, *wR_2_* = 0.1964 | |
| R indices (all data) | *R_1_* = 0.0781, *wR_2_* = 0.2078 | |
| Largest diff. peak and hole | 1.539 and −0.749 e. Å^−3^ | |

^a^R_1_=Σ(||*F_o_*|-|*F_c_*||)/Σ|*F_o_*|. ^b^wR_2_=[Σ[*w*(*F_o_*^2^-*F_c_*^2^ )^2^]/Σ[*w*(*F_o_*^2^)^2^]]^1/2^, *w*=1/[σ^2^(*F_o_*^2^)+(*ap*)^2^+*bp*], where *p*=[max(*F_o_*^2^ , 0)+2*F_c_*^2^]/3.

| Complex | Fe1···Fe2/Å | Fe1-N1  Fe1-N2  /Å | Fe1-S1  Fe1-S2/Å | Fe2-S1  Fe2-S2  /Å | Δ^Fe^/Å | $\angle$Fe1-N3-O1/^o^ | $\angle$Fe2-N4-O2/^o^ | $\angle$Fe2-N5-O3/^o^ |
| --- | --- | --- | --- | --- | --- | --- | --- | --- |
| **[^L1^Fe_2_(NO)_3_]^+^** | 2.7857(8) | 1.669(2)  1.673(2) | 2.2516(9)  2.2469(9) | 2.2435(8)  2.2590(8) | 0.52 | 165.8(2) | 166.6(2) | 174.40(19) |
| **[^L1^Fe_2_(NO)_3_]^0^** | 3.006 | 2.038(2)  2.034(2) | 2.2416(11)  2.2495(11) | 2.3238(13)  2.3377(12) | 0.55 | 154.74(19) | 174.8(2) | 167.5(2) |
| **[^L2^Fe_2_(NO)_3_]^+^** | 2.7130(6) | 2.0556(18)  2.0668(19) | 2.2401(6)  2.2429(6) | 2.2591(7)  2.2516(7) | 0.52 | 171.04(18) | 164.9(2) | 175.5(2) |
| **[^L2^Fe_2_(NO)_3_]^0^** | 2.923 | 2.058(4)  2.061(4) | 2.2341(13)  2.2385(13) | 2.3432(13)  2.3326(14) | 0.53 | 149.9(4) | 171.8(4) | 165.7(5) |
| **[^L2^Fe_2_(NO)_3_]^−^** | 3.168 | 2.241(8)  2.247(7) | 2.380(2)  2.344(2) | 2.372(2)  2.362(2) | 0.85 | 171.4(7) | 172.0(6) | 164.1(7) |
| **[^L3^Fe_2_(NO)_3_]^+^** | 2.7794(18) | 2.072(8)  2.056(8) | 2.261(3)  2.259(3) | 2.256(3)  2.242(3) | 0.50 | 162.7(7) | 160.3(8) | 176.5(8) |
| **[^L3^Fe_2_(NO)_3_]^0^** | 2.912 | 2.0787(16)  2.0759(16) | 2.2431(6)  2.2334(5) | 2.3154(6)  2.3283(6) | 0.54 | 153.09(16) | 170.37(16) | 167.4(2) |
| **[^L3^Fe_2_(NO)_3_]^−^** | 3.138 | 2.297(4)  2.267(4) | 2.3396(13)  2.3508(13) | 2.3692(15)  2.3482(14) | 0.83 | 166.4(5) | 171.9(5) | 168.3(3) |

**Table S6.** Structural parameters of **[^L1^Fe_2_(NO)_3_]^+^**^/0/−^ series.

1. **IR Spectroscopy**.


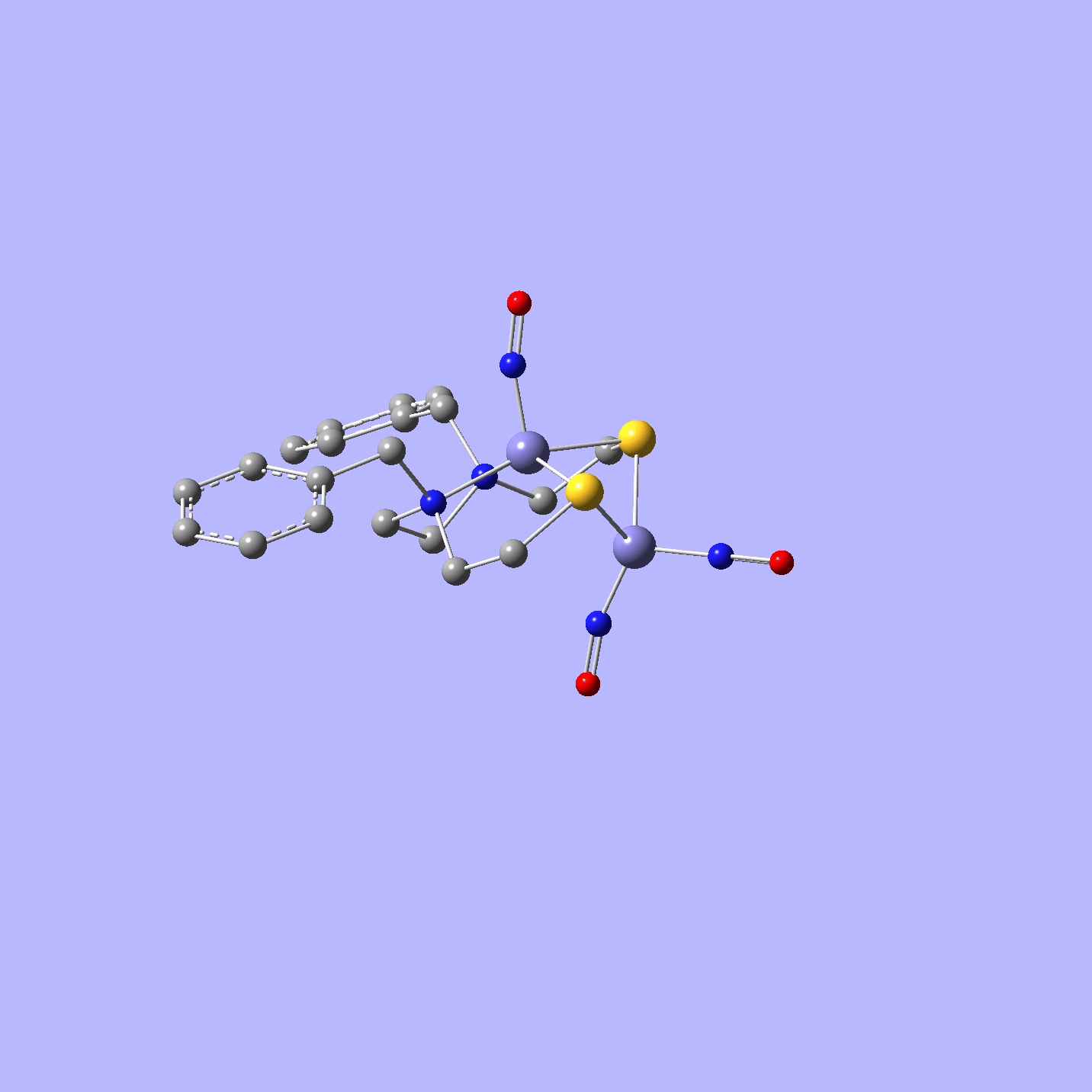

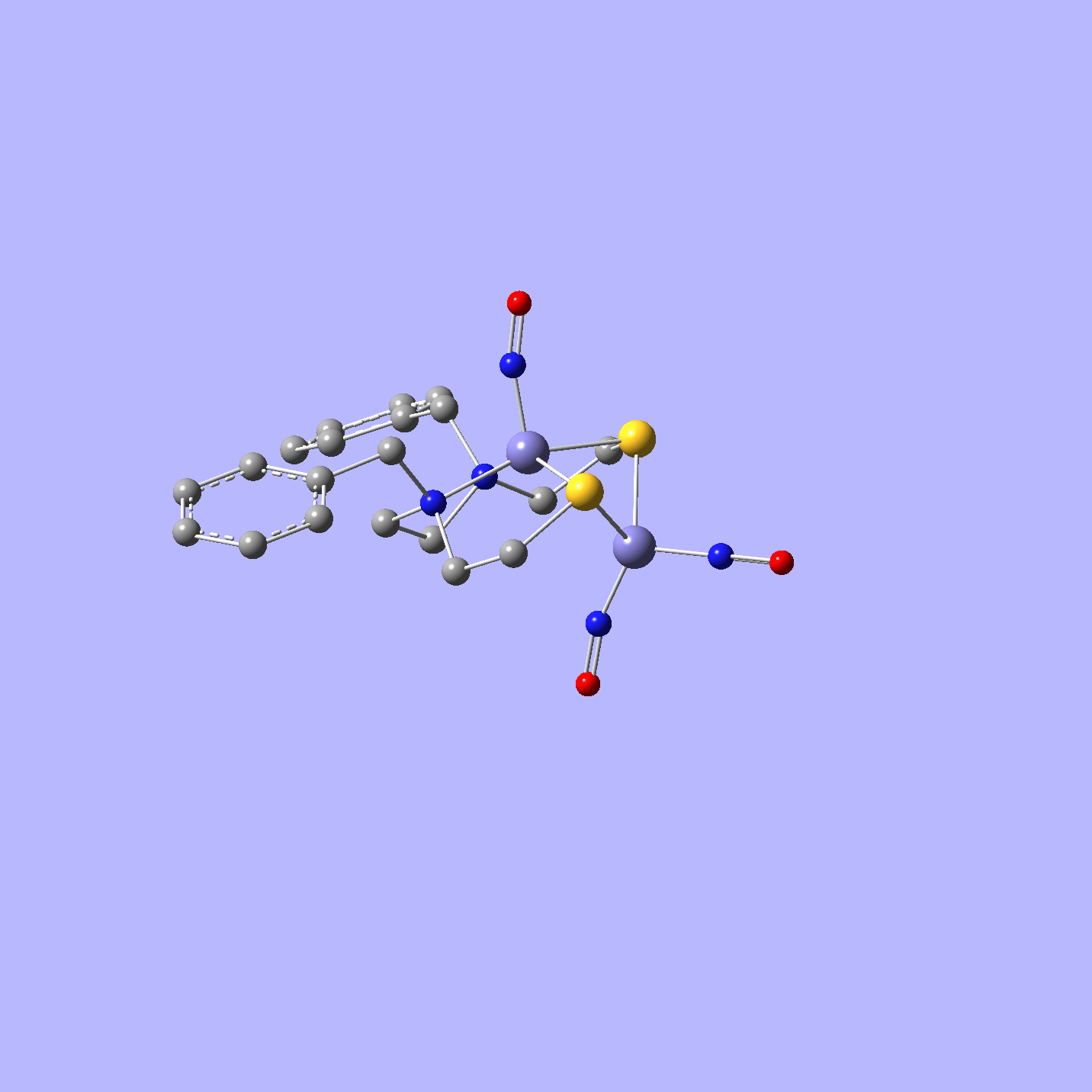

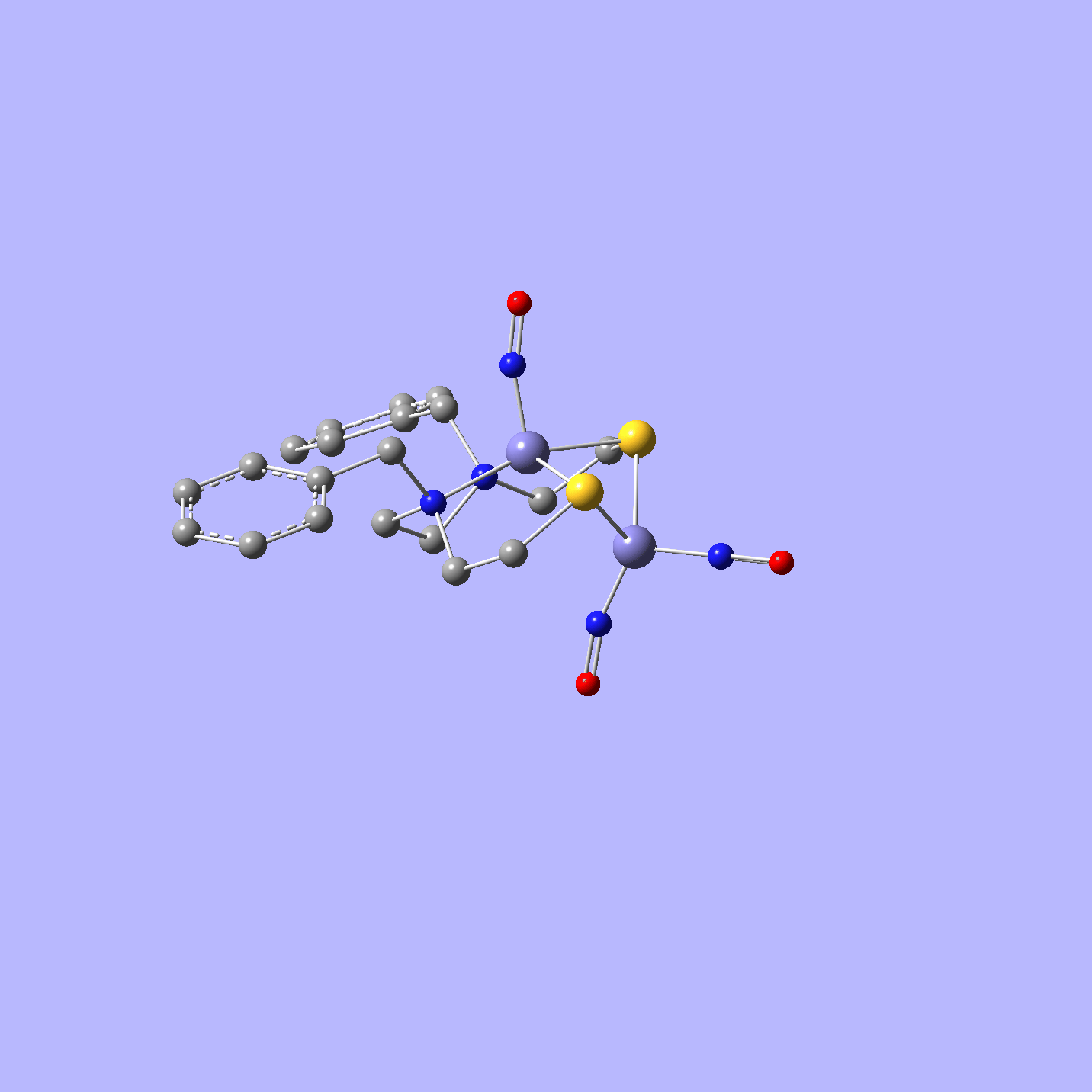


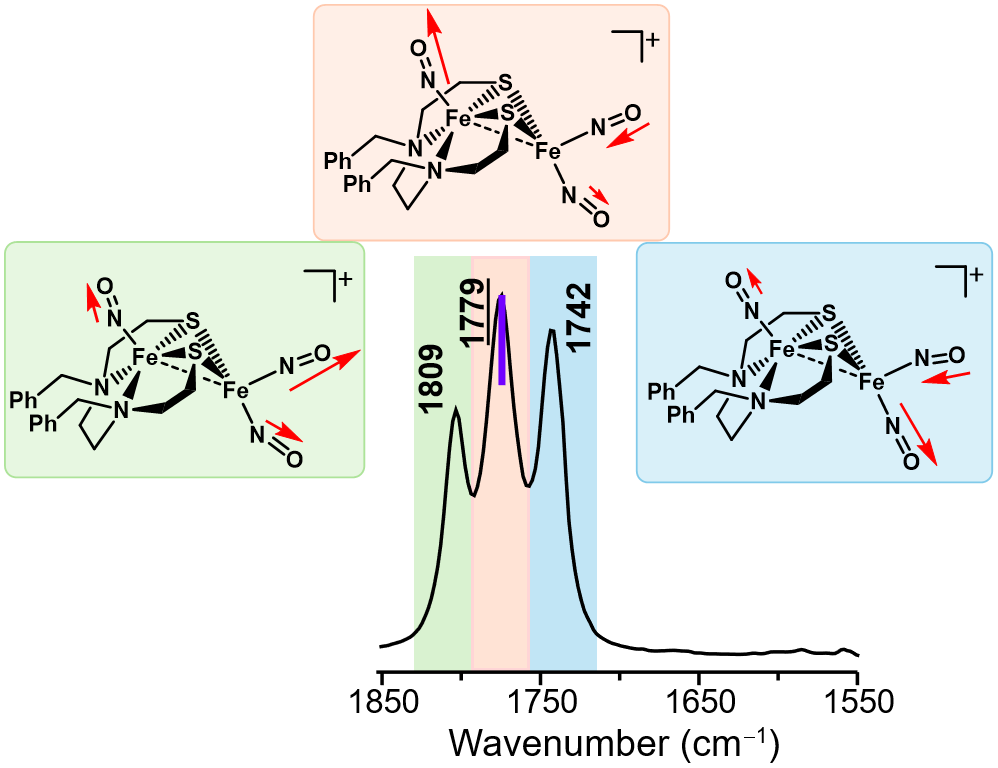


**Figure S11.** FTIR spectrum of **[^L3^Fe_2_(NO)_3_]^+^** with vibrational modes shown *(in animations and pictures)*. The largest arrow represents the strongest nitrosyl stretch, and smaller stretches and contractions are represented by smaller arrows. The length of the arrows is roughly drawn to scale*.*


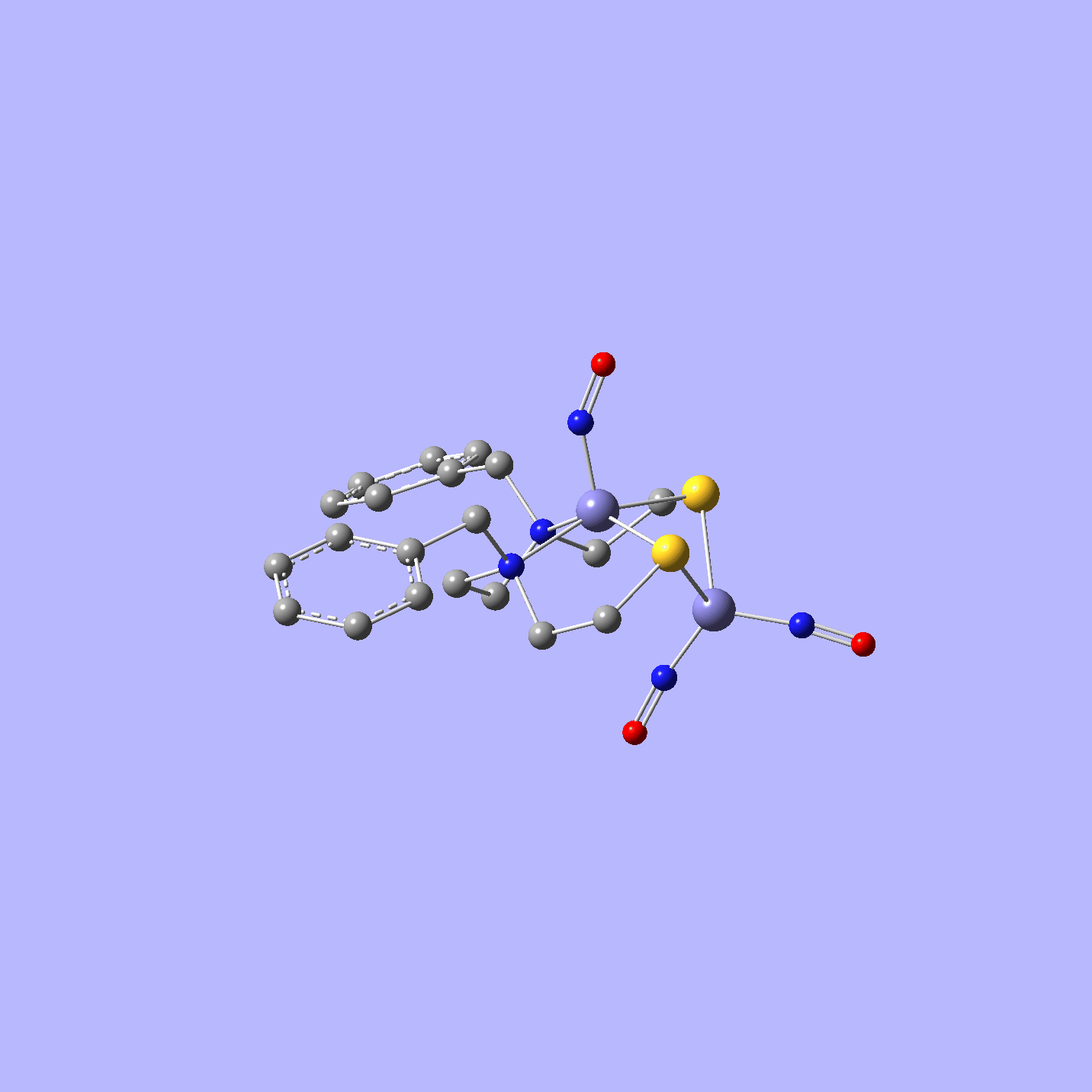

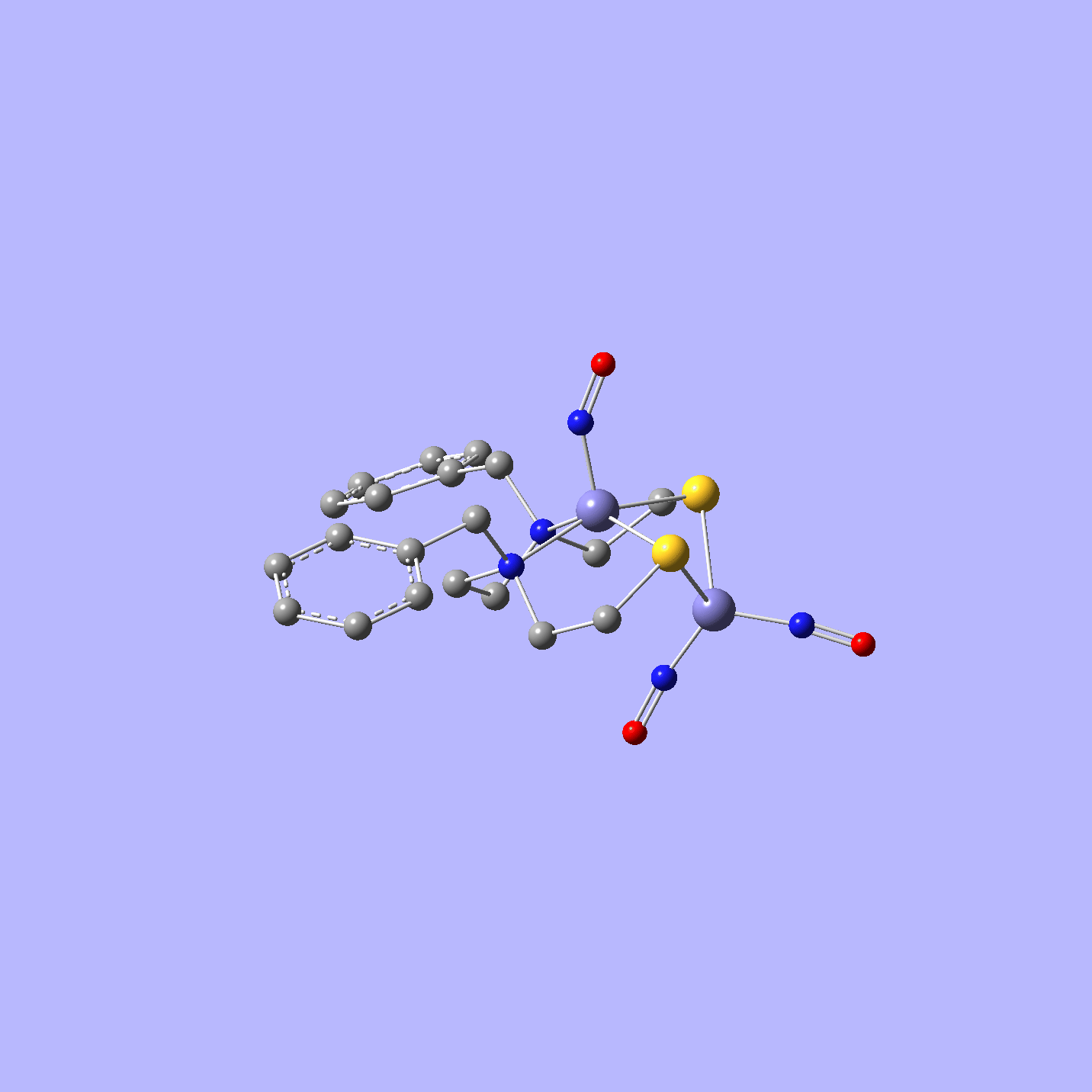

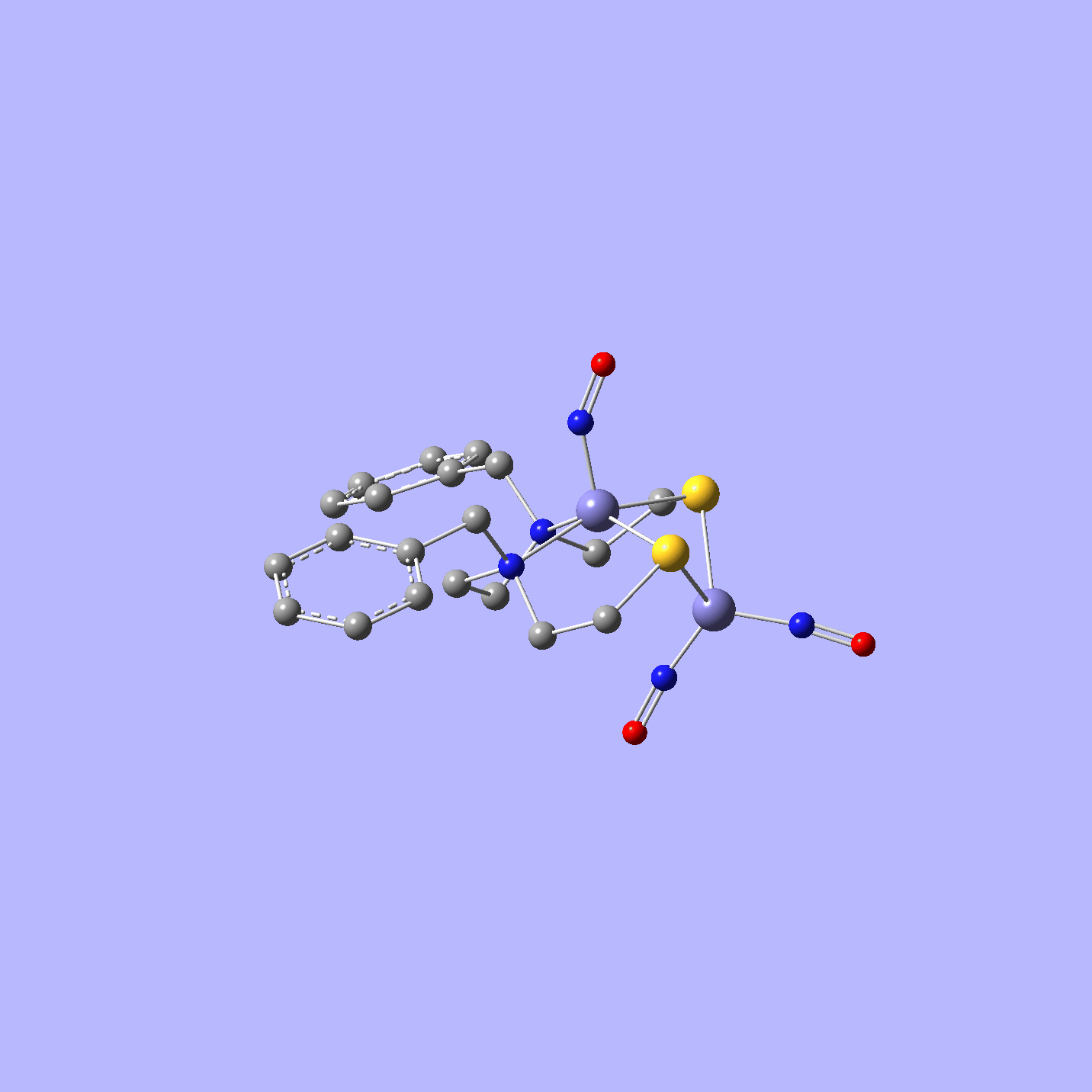


**
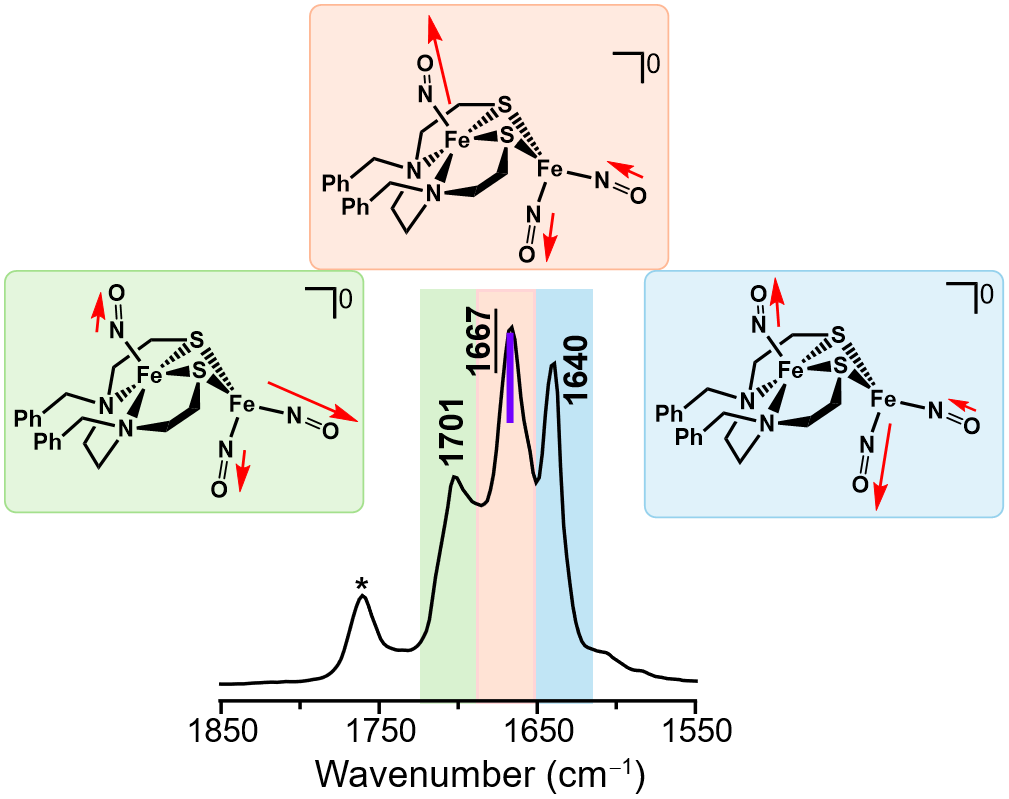
**

**Figure S12.** FTIR spectrum **[^L3^Fe_2_(NO)_3_]^0^** with vibrational modes shown *(in animations and pictures)*. The largest arrow represents the strongest nitrosyl stretch, and smaller stretches and contractions are represented by smaller arrows. The length of the arrows is roughly drawn to scale*.*


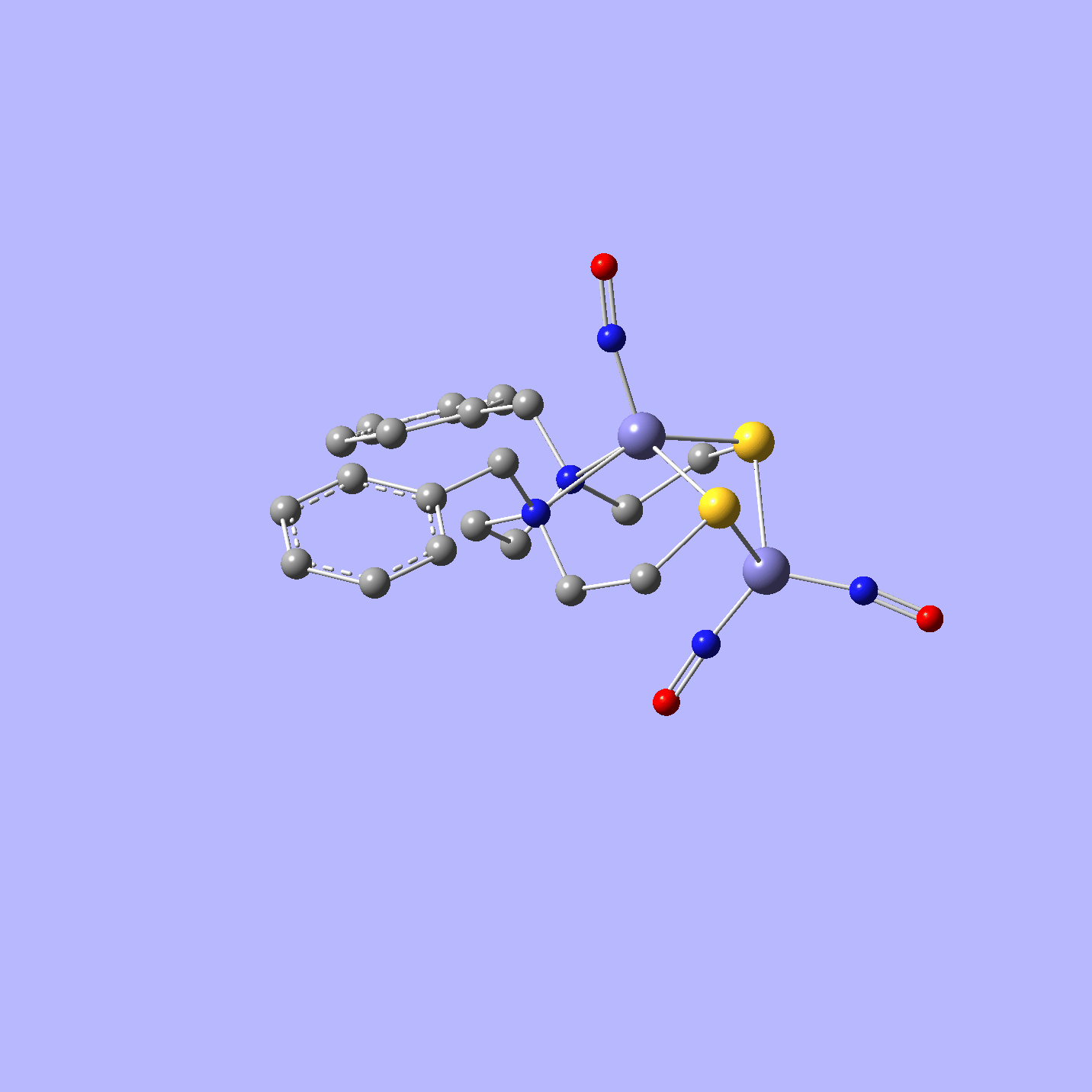

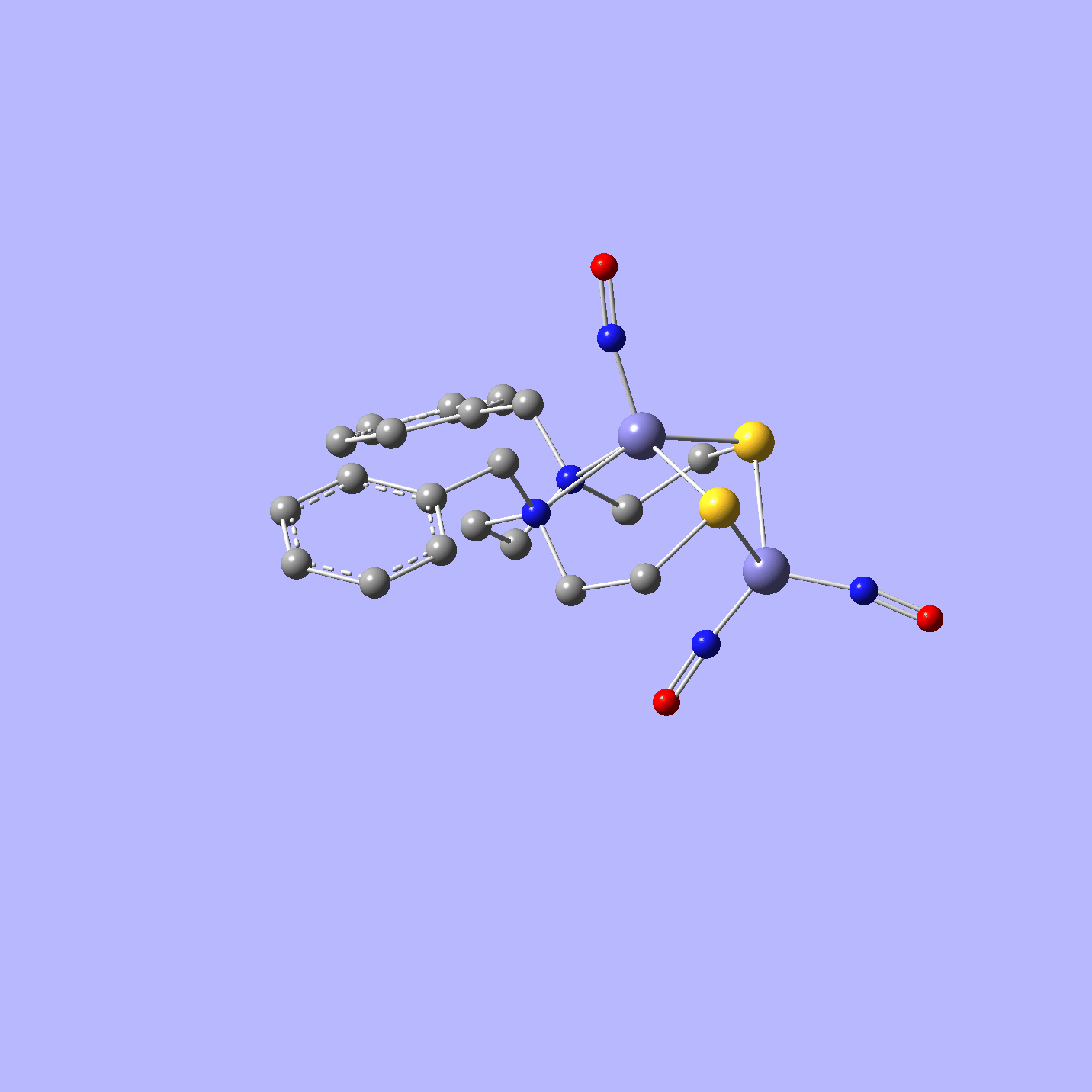

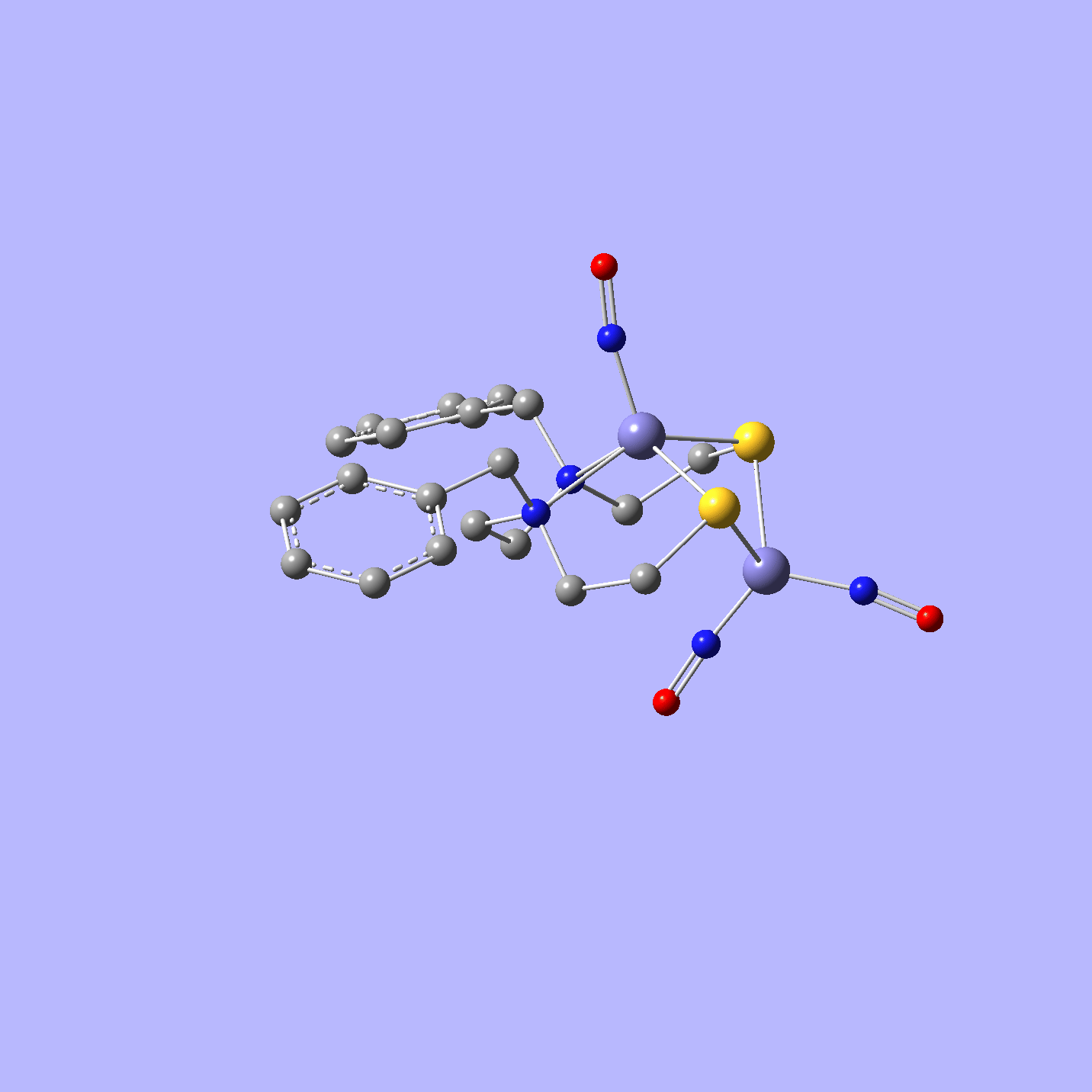


**
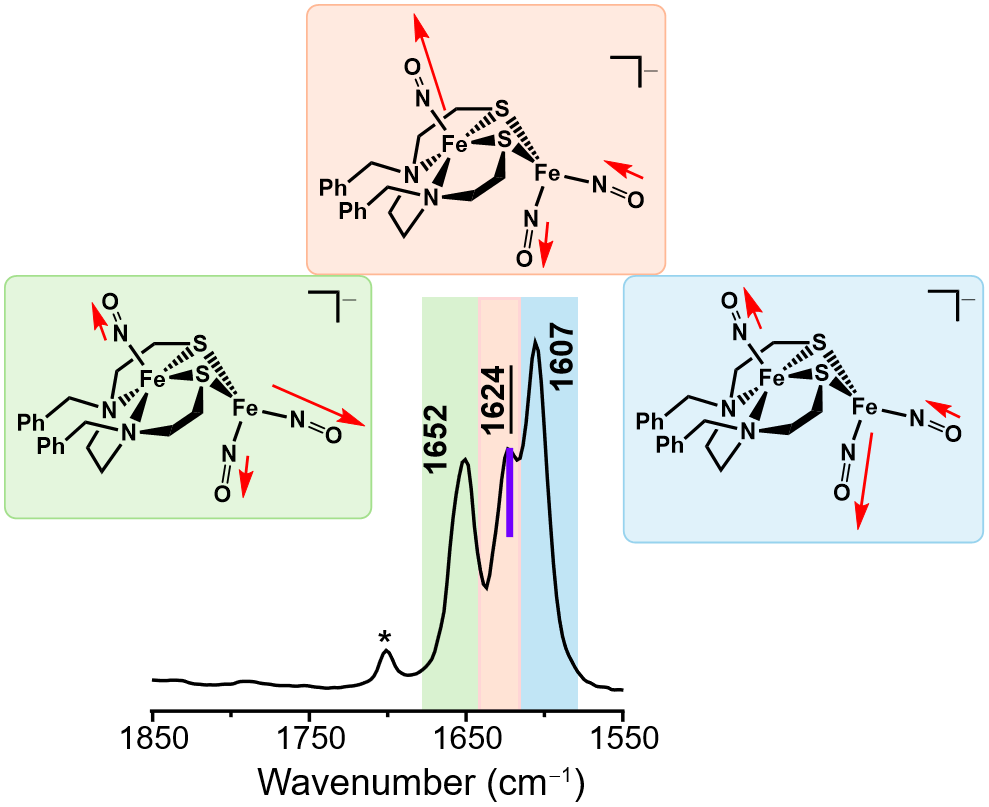
**

**Figure S13.** FTIR spectrum of **[^L3^Fe_2_(NO)_3_]^−^** with vibrational modes shown *(in animations and pictures)*. The largest arrow represents the strongest nitrosyl stretch, and smaller stretches and contractions are represented by smaller arrows. The length of the arrows is roughly drawn to scale*.*

**Table S7.** Experimental and DFT calculated ν(NO) stretches (in vacuum, x-scaling factor used 0.98) of **[^L^Fe_2_(NO)_3_]^+/0/−^** series.

| Complex | ν_expt_ (cm^−1^) | ν_calc_ (cm^−1^) | Ref. |
| --- | --- | --- | --- |
| **[^L1^Fe_2_(NO)_3_]^+^** | 1806, 1770, 1746 | 1812, 1773, 1743 | 17 |
| **[^L1^Fe_2_(NO)_3_]^0^** | 1690, 1662, 1640 | 1730, 1668, 1656 | 17 |
| **[^L2^Fe_2_(NO)_3_]^+^** | 1809, 1779, 1743 | 1812, 1773, 1742 | 9 |
| **[^L2^Fe_2_(NO)_3_]^0^** | 1696, 1668, 1640 | 1742, 1665, 1637 | 9 |
| **[^L2^Fe_2_(NO)_3_]^−^** | 1666, 1637, 1607 | 1722, 1675, 1615 | 9 |
| **[^L3^Fe_2_(NO)_3_]^+^** | 1809, 1779, 1742 | 1812, 1774, 1717 | Tw |
| **[^L3^Fe_2_(NO)_3_]^0^** | 1701, 1667, 1640 | 1751, 1654, 1639 | Tw |
| **[^L3^Fe_2_(NO)_3_]^−^** | 1655, 1624, 1607 | 1694, 1628, 1604 | Tw |

1. **ESI-Mass Spectrometry.**


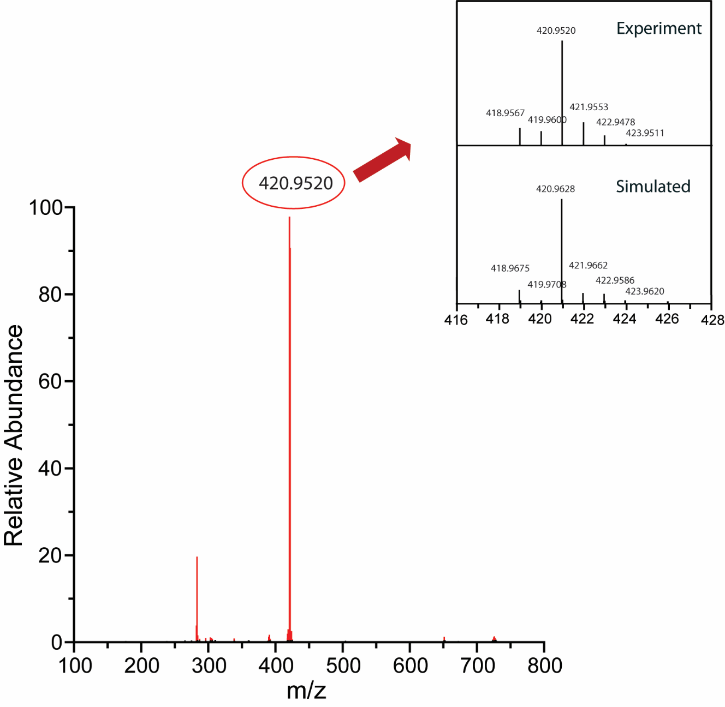


**Figure S14.** ESI-MS data of **[^L1^Fe(^15^NO)**·**Fe(^14^NO)_2_]^+^**.


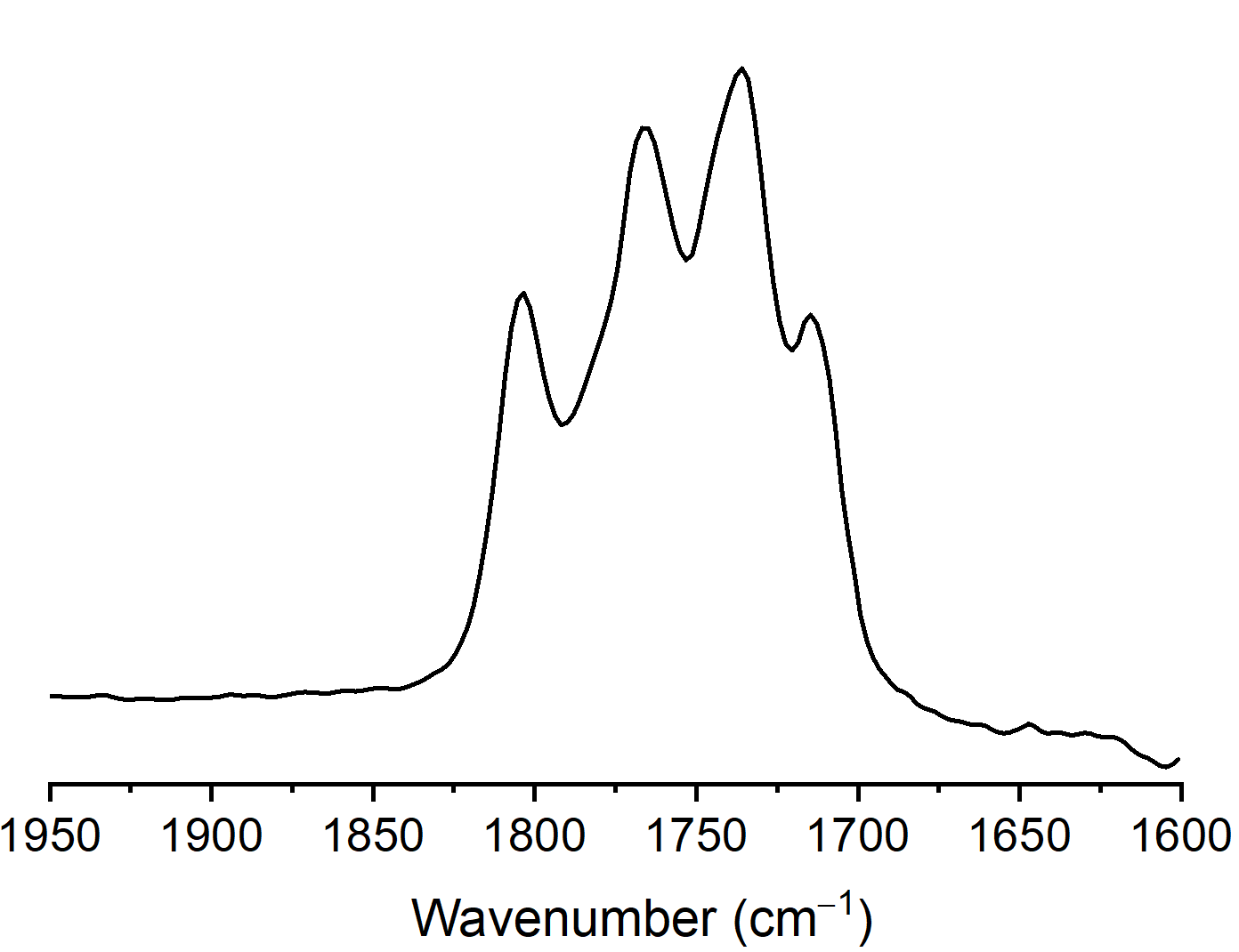


**Figure S15.** IR taken from the reaction solution of NO purging to **[^L^Fe_2_(^15^NO)_3_]^+^** at room temperature.

**
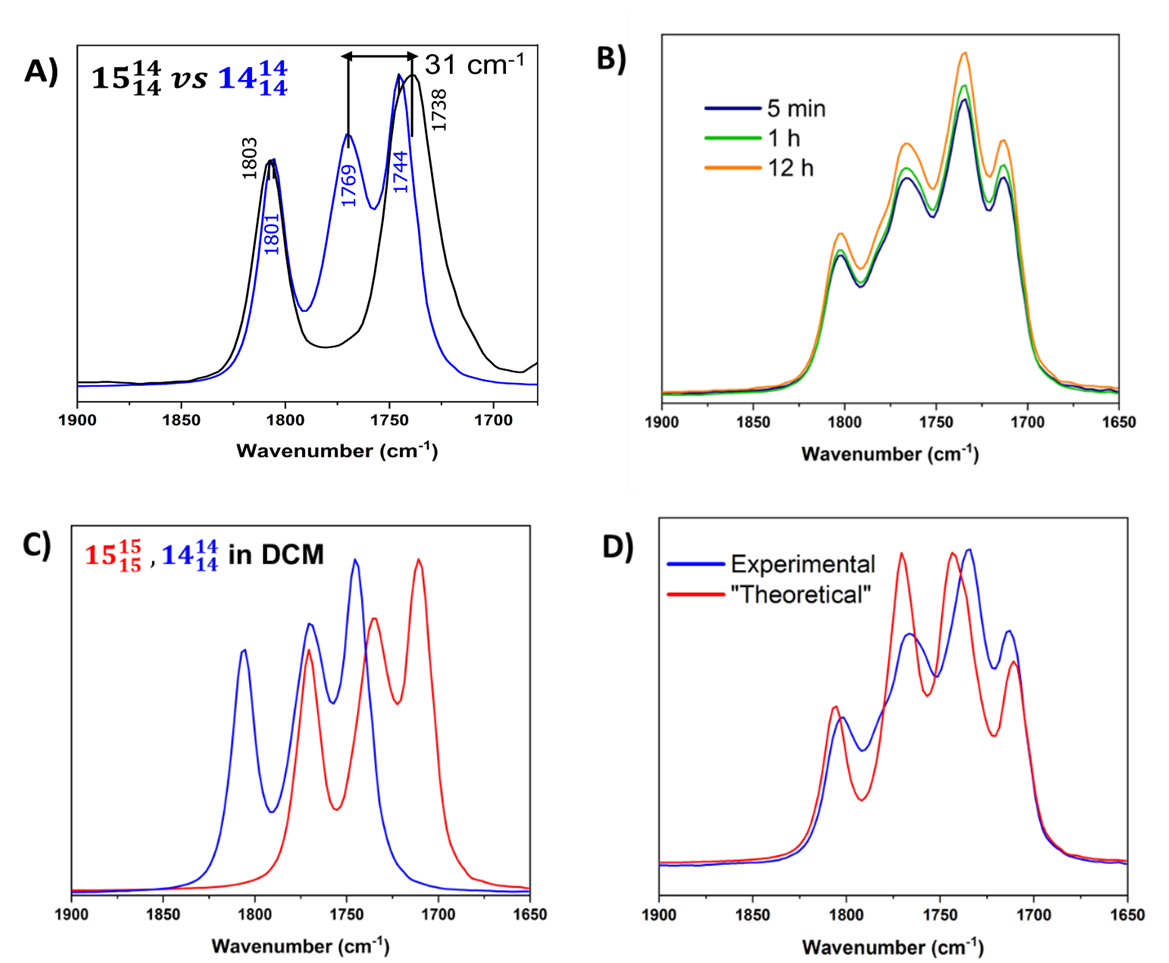
**

Figure S16. A) FTIR spectra comparison of singly labeled $\boldsymbol{15}_{\boldsymbol{14}}^{\boldsymbol{14}}$ and natural abundance $\boldsymbol{14}_{\boldsymbol{14}}^{\boldsymbol{14}}$. B) FTIR changes of the $\boldsymbol{15}_{\boldsymbol{14}}^{\boldsymbol{14}}$ product over time at room temperature. C) Comparison of the fully labeled $\boldsymbol{15}_{\boldsymbol{15}}^{\boldsymbol{15}}$ and natural abundance $\boldsymbol{14}_{\boldsymbol{14}}^{\boldsymbol{14}}$ species. D) Combined normalized spectra of fully labeled $\boldsymbol{15}_{\boldsymbol{15}}^{\boldsymbol{15}}$ and natural abundance $\boldsymbol{14}_{\boldsymbol{14}}^{\boldsymbol{14}}$ species compared to the experimental scrambled spectra.

Note: $\boldsymbol{15}_{\boldsymbol{14}}^{\boldsymbol{14}}$ = [^L1^Fe(^15^NO)·Fe(^14^NO)_2_]^+^; $\boldsymbol{14}_{\boldsymbol{14}}^{\boldsymbol{14}}$ = [^L1^Fe_2_(^14^NO)_3_]^+^ and $\boldsymbol{15}_{\boldsymbol{15}}^{\boldsymbol{15}}$ =[^L1^Fe_2_(^15^NO)_3_]^+^.


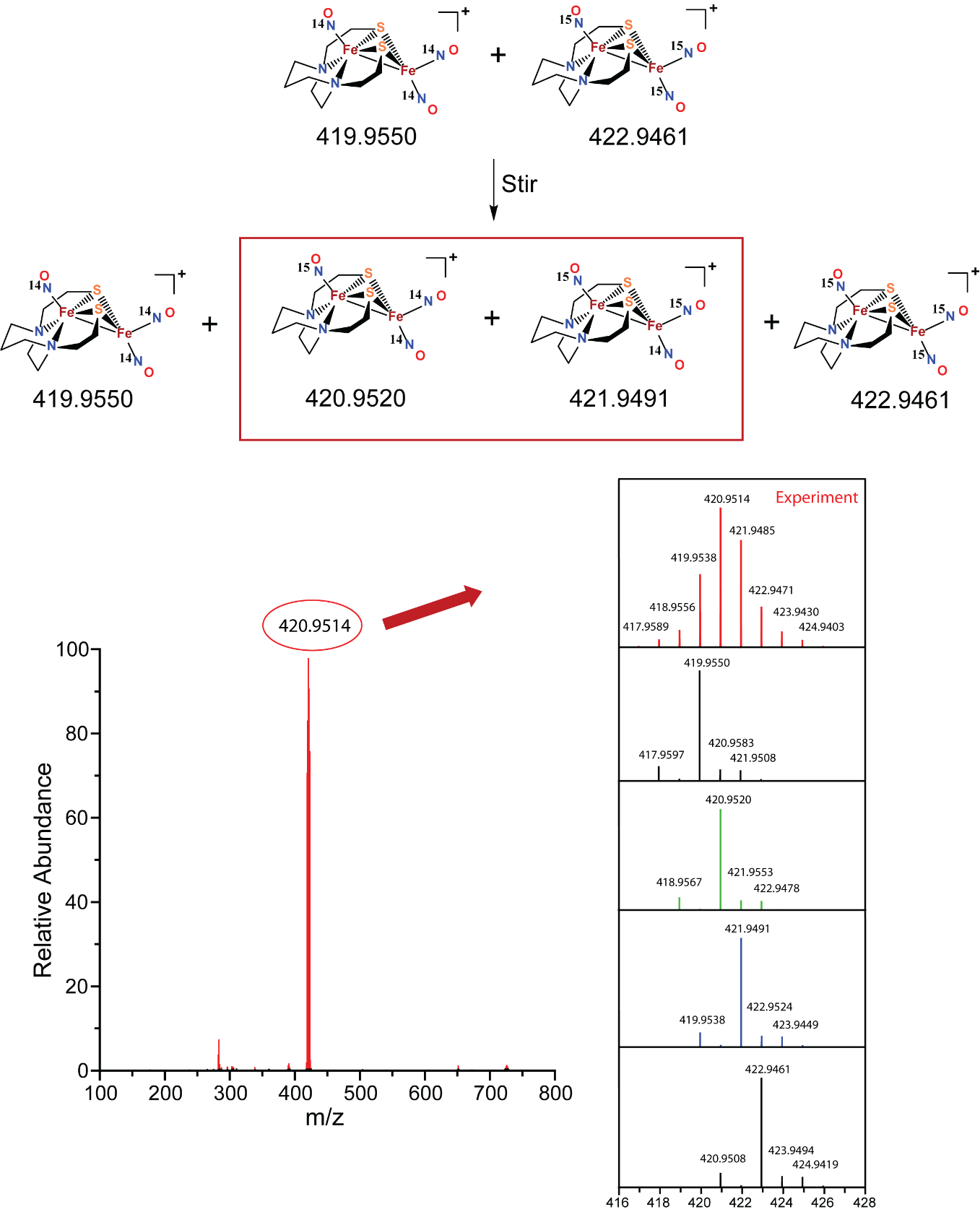


**Figure S17.** ESI-MS data from the mixture of **[^L1^Fe_2_(^14^NO)_3_]^+^** and **[^L1^Fe_2_(^15^NO)_3_]^+^** in CH_2_Cl_2_; showing NO scrambling.

1. **^15^N NMR Spectroscopy**.


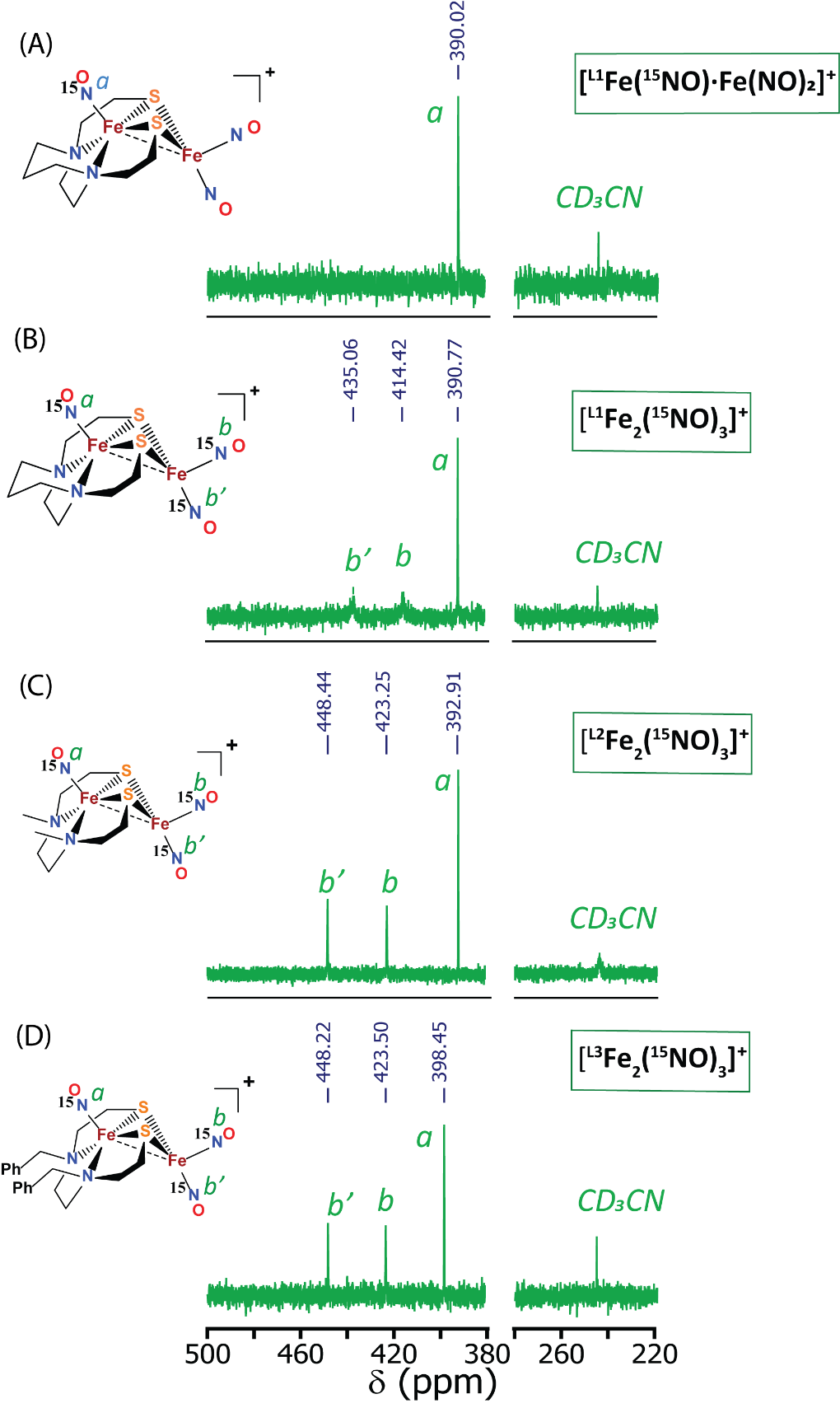


**Figure S18.** ^15^N NMR (CD_3_CN, 295K) of A) **[^L1^Fe(^15^NO)·Fe(^14^NO)_2_]^+^**, B) **[^L1^Fe_2_(^15^NO)_3_]^+^**, C) **[^L2^Fe_2_(^15^NO)_3_]^+^**, and D) **[^L3^Fe_2_(^15^NO)_3_]^+^**.

**Table S8.** N-15 NMR peak positions of the complexes in CD_3_CN.

| Complexes | N-15 NMR signals in ppm (recorded in CD_3_CN) | | |
| --- | --- | --- | --- |
|  | Fe(NO) unit | Fe(NO)_2_ units | |
| **[^L1^Fe(^15^NO)·Fe(^14^NO)_2_]^+^** | 390.02 | - | - |
| **[^L1^Fe_2_(^15^NO)_3_]^+^** | 390.77 | 414.42 | 435.06 |
| **[^L2^Fe_2_(^15^NO)_3_]^+^** | 392.91 | 423.25 | 448.44 |
| **[^L3^Fe_2_(^15^NO)_3_]^+^** | 398.45 | 423.50 | 448.22 |

1. **Calculation of Magnetic Susceptibility using the Evans Method**.

The magnetic susceptibility of **[^L3^Fe_2_(NO)_3_]^+^**, **[^L3^Fe_2_(NO)_3_]^0^**, and **[^L3^Fe_2_(NO)_3_]^−^** was determined by the Evans method. The ^19^F NMR of **[^L3^Fe_2_(NO)_3_]^+^**, **[^L3^Fe_2_(NO)_3_]^0^**, and **[^L3^Fe_2_(NO)_3_]^−^** were measured in a 400 MHz NMR machine at 295 K. In a NMR tube, 15.9 mg of **[^L3^Fe_2_(NO)_3_]^+^** was taken and dissolved in 0.5 mL of deuterated CD_3_CN, and 1.0 μL of C_6_F_6_ was added under an argon atmosphere. Next, an inner tube containing a mixture of 0.5 mL of deuterated THF and 1.0 μL of C_6_F_6_ was placed into the NMR tube, and then ^19^F NMR of **[^L3^Fe_2_(NO)_3_]^+^** was measured at 295 K. Similarly, we measured ^19^F NMR of **[^L3^Fe_2_(NO)_3_]^0^** and **[^L3^Fe_2_(NO)_3_]^−^** at 295 K using 9.8 and 10.07 mg of complexes, respectively. The calculated diamagnetic susceptibility, **χ**D, of **[^L3^Fe_2_(NO)_3_]^0^** and **[^L3^Fe_2_(NO)_3_]^−^** were around -0.00025 and -0.00027, respectively, which are close to [–(mol.wt.)/2]/1000000.^18^ Cationic species, **[^L3^Fe_2_(NO)_3_]^+^**, is showing almost no magnetic moment value indicating *S*=0, whereas the neutral, **[^L3^Fe_2_(NO)_3_]^0^**, and the anionic species, **[^L3^Fe_2_(NO)_3_]^−^**, are showing the magnetic moment values of 1.78 and 2.91 BM. These results imply that the cationic species is diamagnetic, whereas the neutral species contains one unpaired electron (*S*=1/2), and the anionic species contains two unpaired electrons (*S*=1).


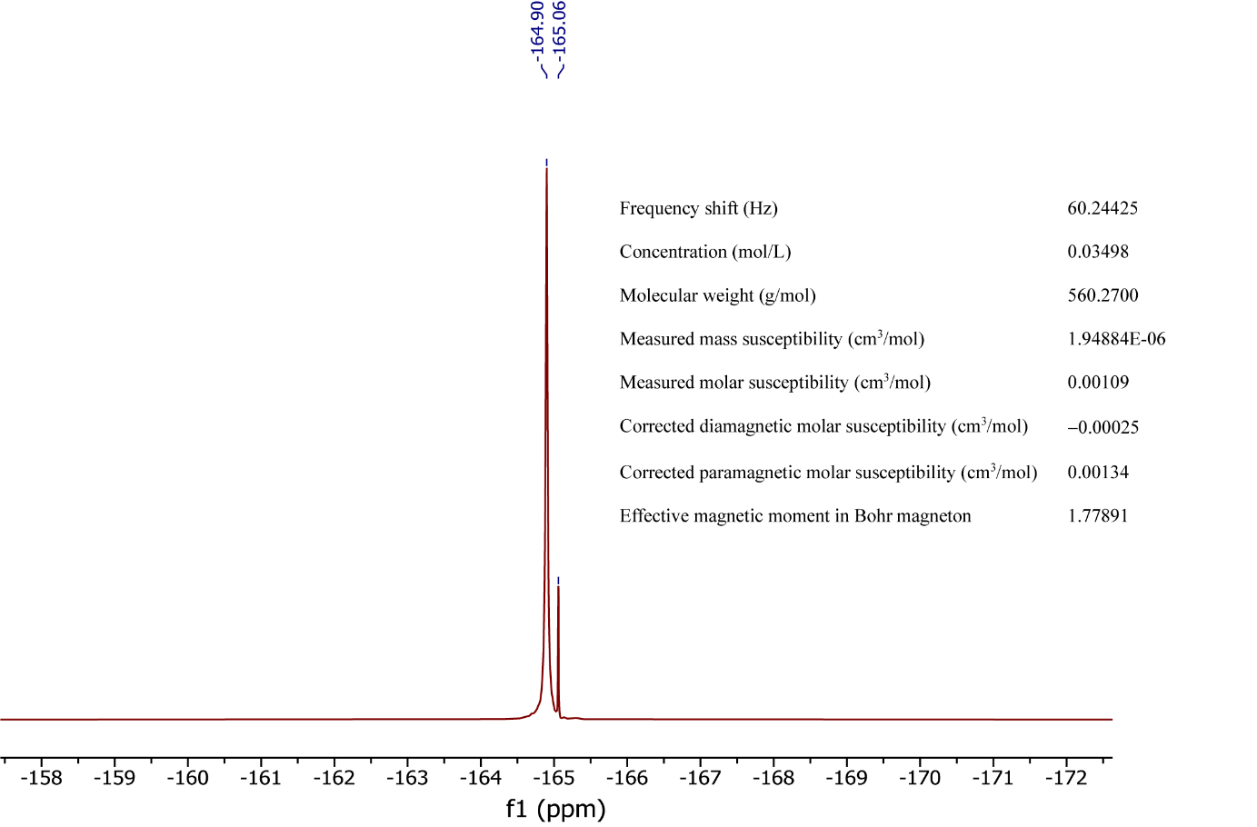


**Figure S19.** ^19^F NMR of **[^L3^Fe_2_(NO)_3_]^0^** at 295 K in THF-d_8_ using a 400 MHz NMR under Ar referenced to C_6_F_6_ at −164.90 ppm.


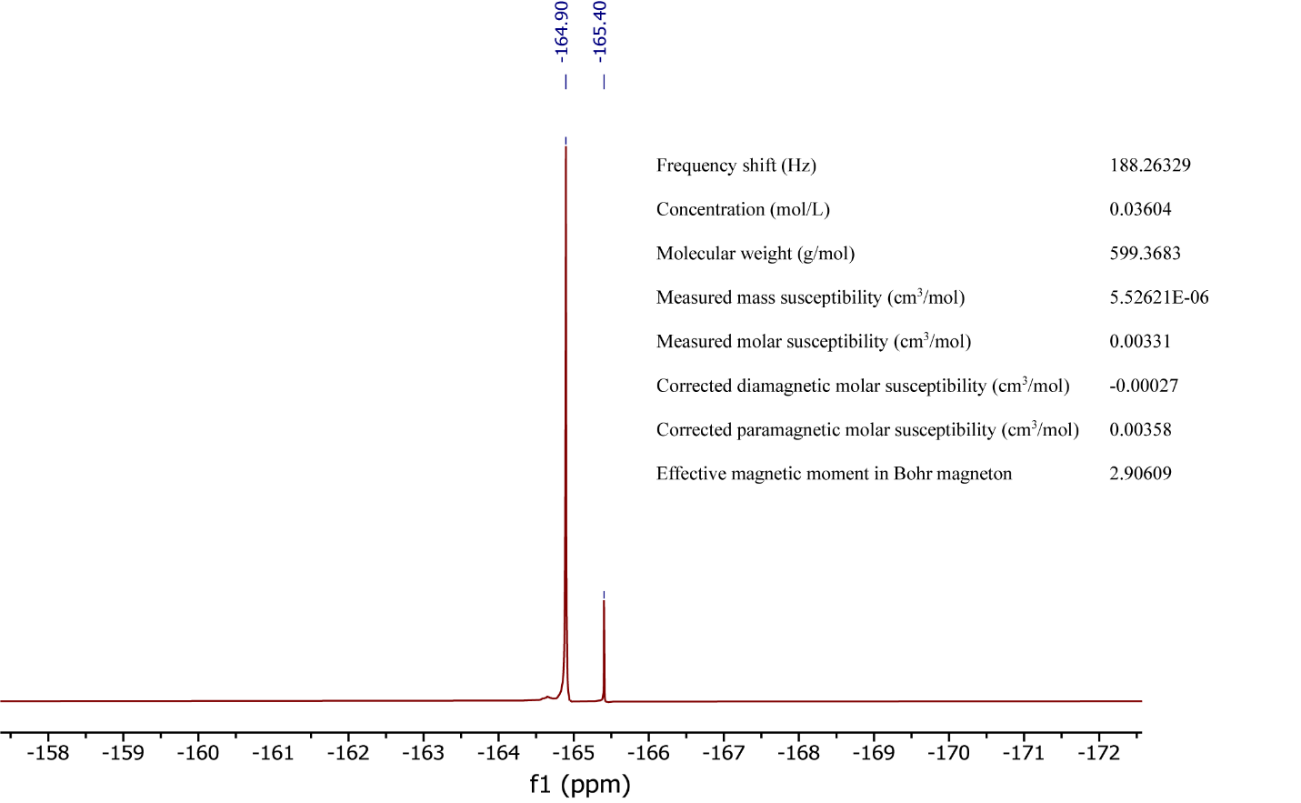


**Figure S20.** ^19^F NMR of **[^L3^Fe_2_(NO)_3_]^−^** at 295 K in THF-d_8_ using a 400 MHz NMR under Ar referenced to C_6_F_6_ at −164.90 ppm.

1. **EPR Spectroscopy**.

***EPR spectra of Cationic species (S=0).***


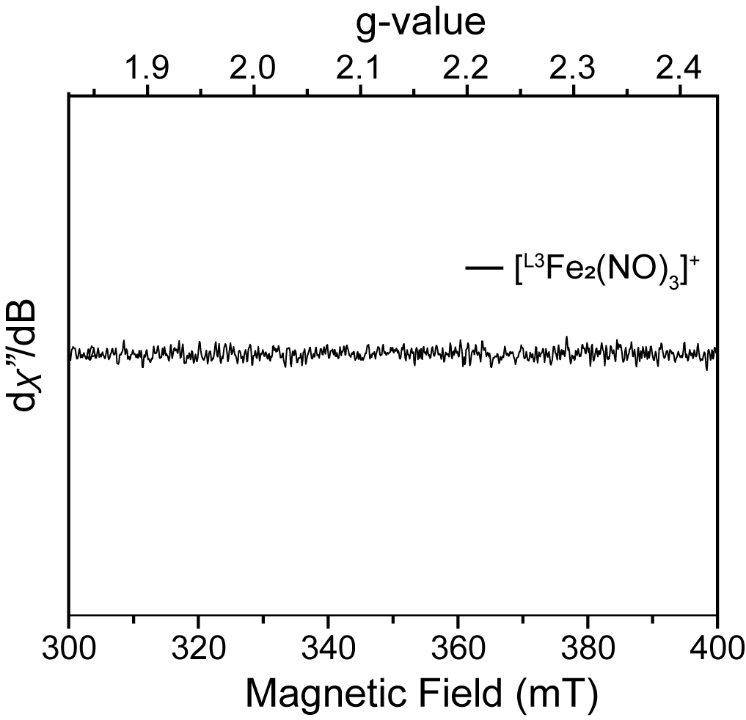


**Figure S21.** X-band EPR spectrum (black line) of **[^L3^Fe_2_(NO)_3_]^+^** in CH_3_CN at 295 K.

***EPR Analysis of S = 1/2 species***.


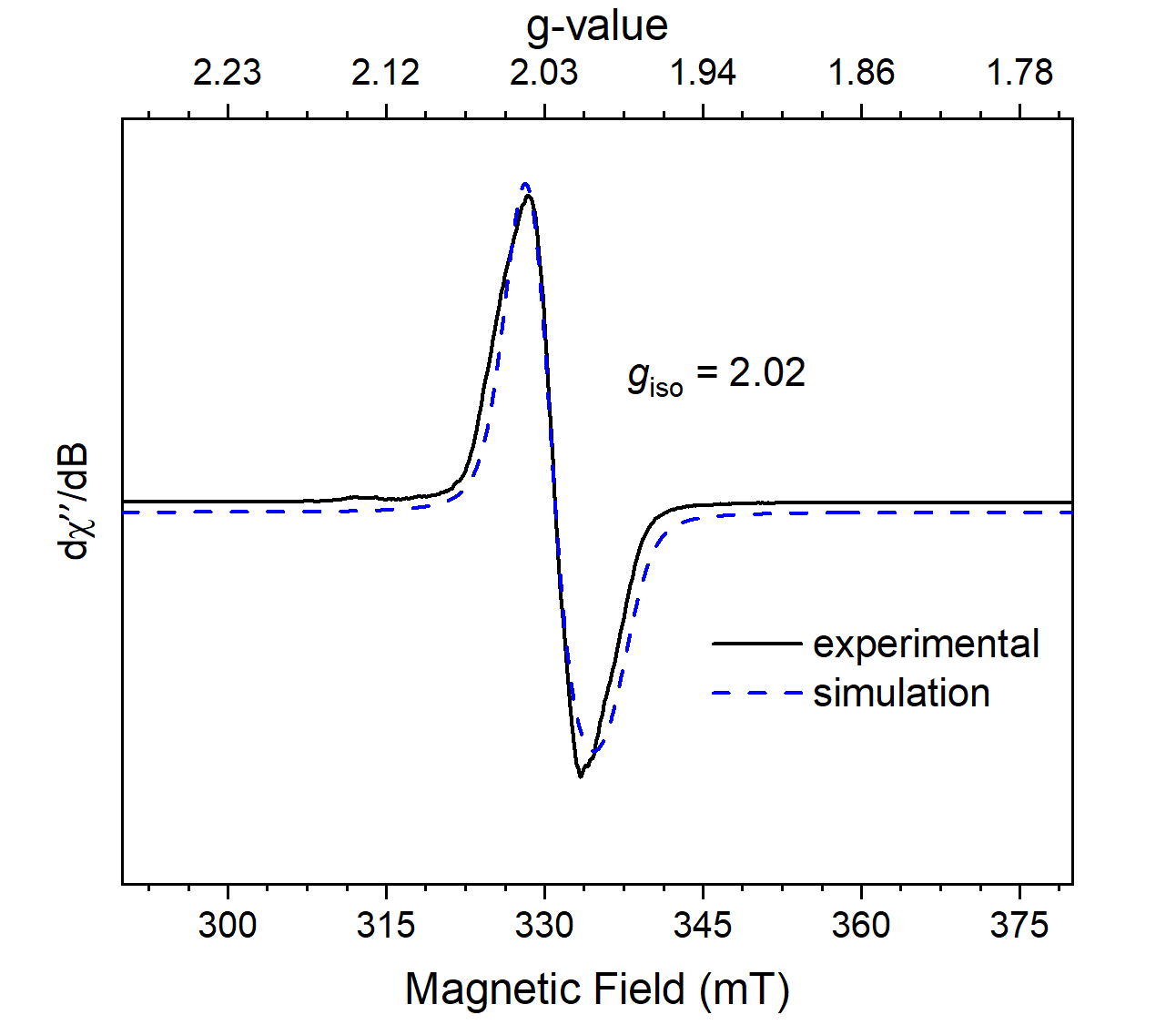


**Figure S22.** X-band EPR spectrum (black line) of **[^L3^Fe_2_(NO)_3_]^0^** in THF at 4 K (Microwave frequency 9.357987 GHz); 1 G modulation amplitude; 100 kHz modulation frequency; 40 ms conversion time; 632 mW microwave power. Simulation parameters (blue dashed line) *g* = [1.99 2.029 2.029] with lwpp broadening (in mT) = [3.79 1.19].


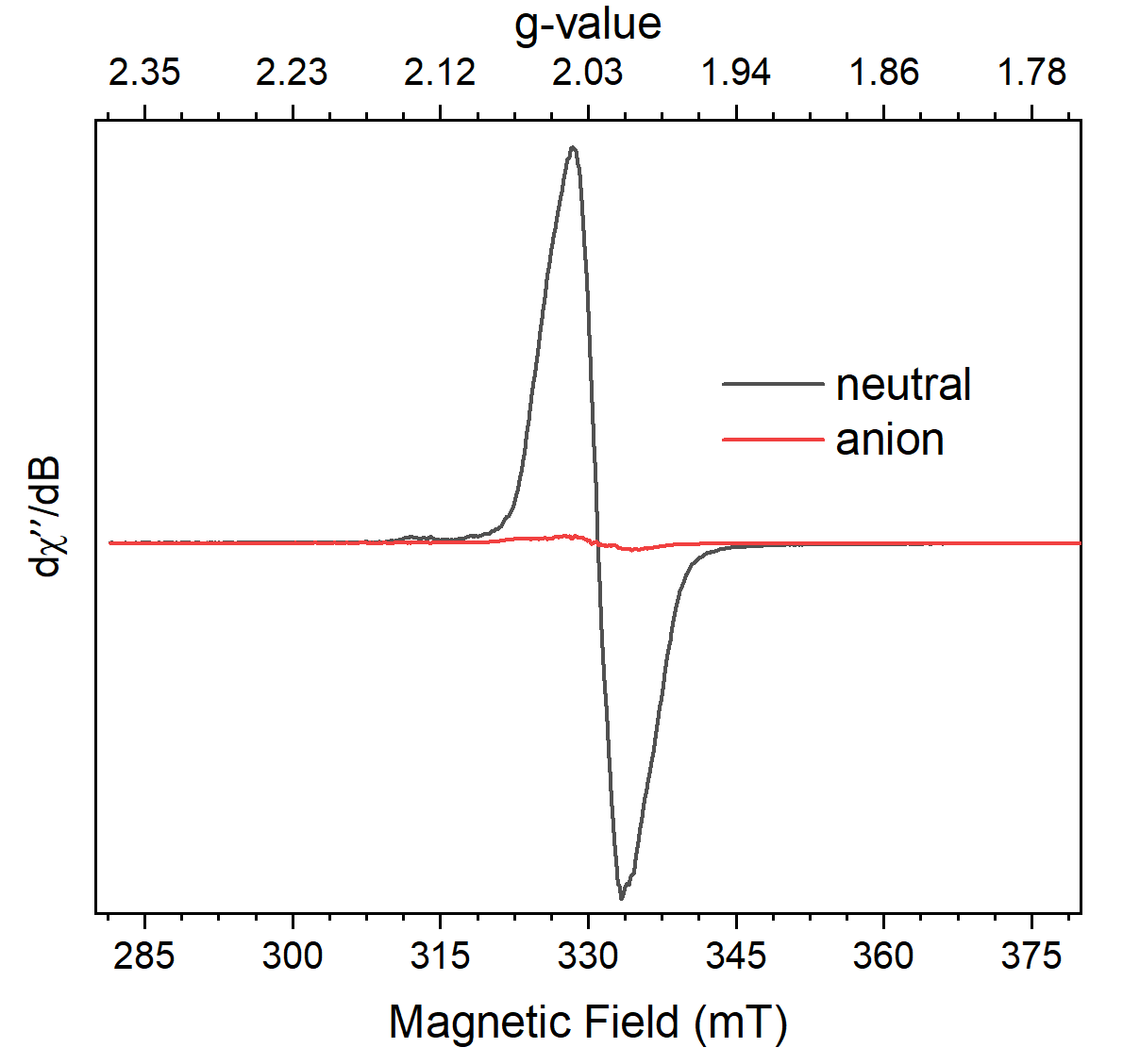


**Figure S23.** X-band EPR spectra of **[^L3^Fe_2_(NO)_3_]^0^** (black trace; neutral) and **[^L3^Fe_2_(NO)_3_]^−^** (red trace; anion) in THF at 4 K before and after adding 1 equiv. of KHBEt_3_ (Microwave frequency: 9.357987 GHz for **[^L3^Fe_2_(NO)_3_]^0^** and 9.353653 GHz for **[^L3^Fe_2_(NO)_3_]^−^**); 1 G modulation amplitude; 100 kHz modulation frequency; 40 ms conversion time; 632 mW microwave power.


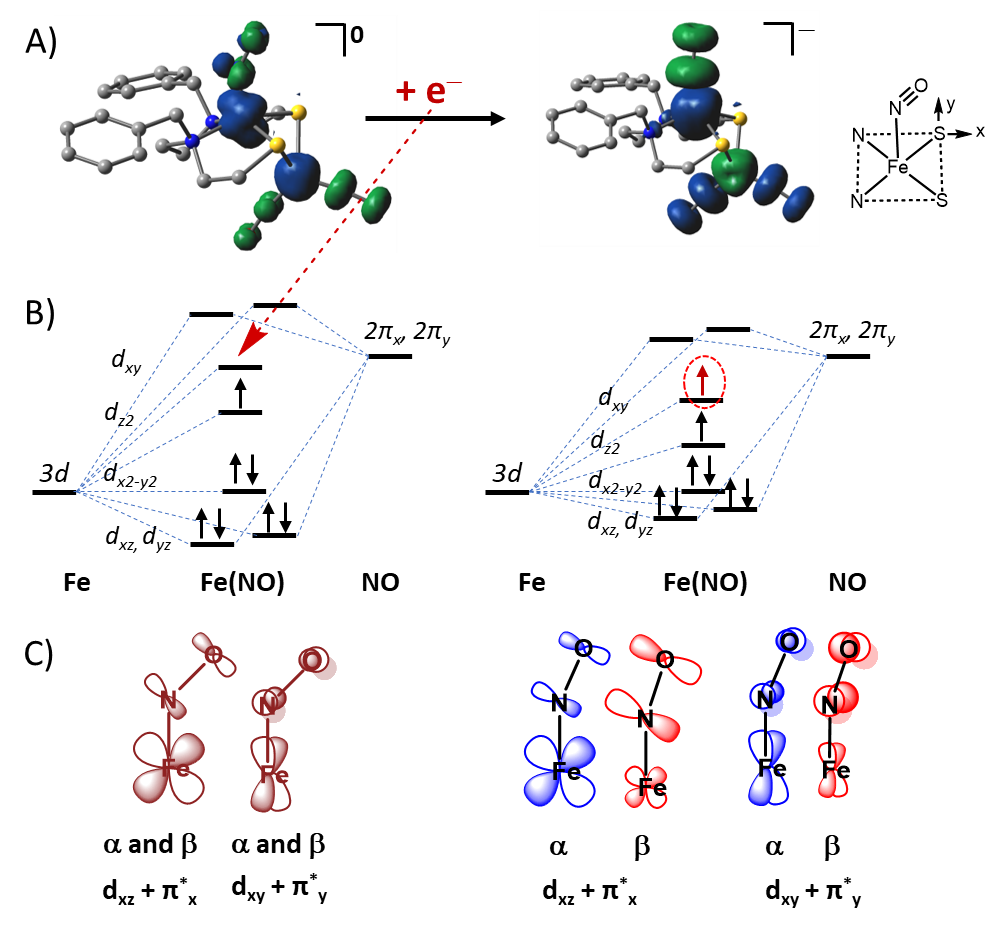


Figure S24. A) Spin density plots (isovalue=0.005 a.u.) of [^L3^Fe_2_(NO)_3_]^0^ and [^L3^Fe_2_(NO)_3_]^−^ calculated from DFT calculation; B) the geometric and electronic structure changes after the reduction of [^L3^Fe_2_(NO)_3_]^0^ to [^L3^Fe_2_(NO)_3_]^−^.

1. **Mössbauer Spectroscopy**.

In some spectra, the lowest temperature attainable was 7 K, and in these, **Fe_ox_** exhibited a sharp doublet as shown in the blue simulation line of Figure 5B (see main manuscript); i.e., there is no evidence of magnetic hyperfine. In spectra in which temperatures of 4.8 K could be obtained, **Fe_ox_** exhibited broadening that we assign to magnetic hyperfine interactions. One explanation is that those interactions collapsed between 4.8 K and 7 K. (Alternatively, some samples may not have been fully reduced into the neutral state.) The other difference among samples was the temperature at which the magnetic hyperfine interactions associated with **Fe_red_** collapsed; in some samples (Figure 5C, see main manuscript), this occurred by 93 K; in other samples (Figure S25), this was not evident by 104 K, but became more noticeable by 150 K (Figure S25).

**
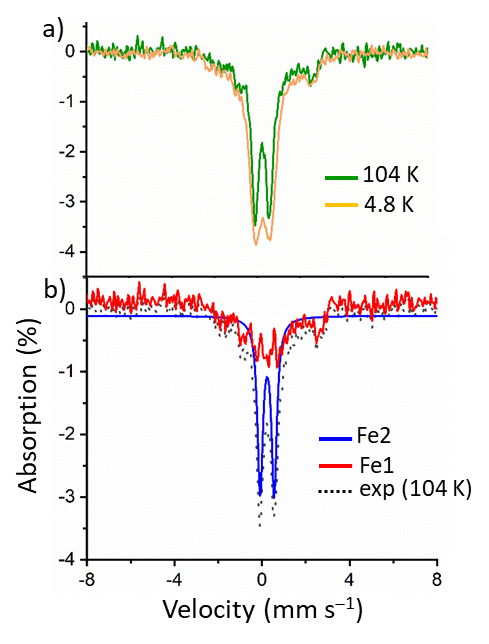
**

**Figure S25.** Temperature-dependent Mössbauer spectra of the neutral complex [^L3^Fe_2_(NO)_3_]^0^. Panel a), at 4.8 K, the spectrum (gold line) displays a broad doublet with evident magnetic hyperfine interactions, whereas at 104 K (green line), the doublet has sharpened significantly as hyperfine collapsed. Panel b) Fit of **Fe_ox_** at 104 K (blue line) with difference due to **Fe_red_** shown in Red.

1. **S K-edge XAS**.

Sulfur K-edge X-ray absorption spectroscopy (XAS) is a powerful technique for directly probing the chemical states of sulfur (e.g., thiolate, sulfenate, and sulfinate), its bonding modes, and the covalency of metal–sulfur bonds. Mononuclear and dinuclear iron complexes exhibit distinct XAS features. Additionally, XAS is sensitive to terminal thiolates, which induce a pre-edge peak shift, reflecting substantial charge contributions.


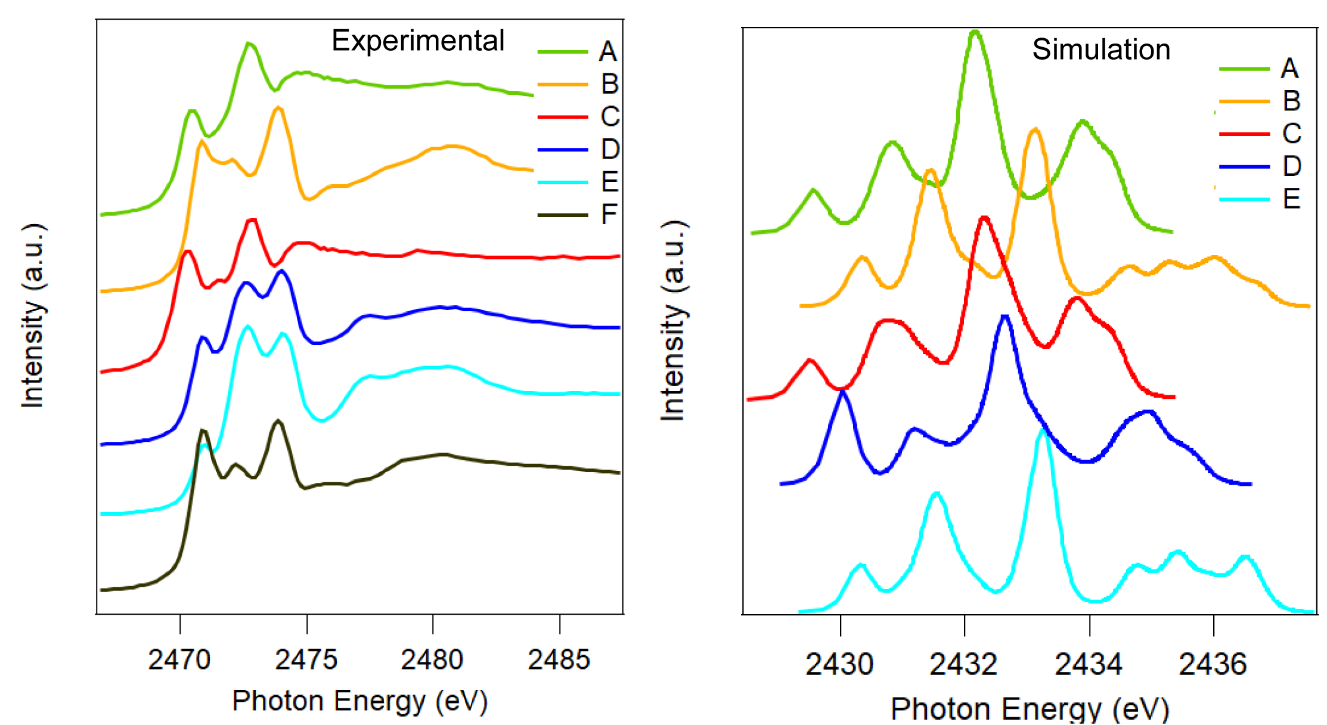


**Figure S26.** Left panel: Experimental S K-edge XAS spectra of **^L1^Fe(NO)** (A), **[^L1^Fe_2_(NO)_3_]^+^** (B), **^L2^Fe(NO)** (C), **[^L2^Fe_2_(NO)_3_]^+^** (D), **[^L2^Fe_2_(NO)_3_]^0^** (E), and **[^L3^Fe_2_(NO)_3_]^+^** (F); Right panel: Simulated spectra of the corresponding experimental spectra.

1. **Computational Modeling**.

| Complexes | TPSS | TPSSh |
| --- | --- | --- |
| **[^L3^Fe_2_(NO)_3_]^+^** | 0.2245 | 0.3499 |
| **[^L3^Fe_2_(NO)_3_]^0^** | 0.5132 | 0.5820 |
| **[^L3^Fe_2_(NO)_3_]^−^** | 1.0883 | 1.2158 |

**Table S9.** Spin values obtained from the singlet point calculation for **[^L3^Fe_2_(NO)_3_]^+/0/−^** species.

**Table S10.** Comparison of experimental and computed parameters of the {Fe(NO)}^7/8^ and {Fe(NO)}^9/10^ moieties in **[^L3^Fe_2_(NO)_3_]^+/0/−^** series.

|  | **[^L3^Fe_2_(NO)_3_]^+^** | | **[^L3^Fe_2_(NO)_3_]^0^** | | **[^L3^Fe_2_(NO)_3_]^−^** | |
| --- | --- | --- | --- | --- | --- | --- |
| multiplicity | BS singlet | | Doublet | | Triplet | |
| E-F count | **{Fe(NO)}^7^** | | **{Fe(NO)}^7^** | | **{Fe(NO)}^8^** | |
| d-electron configuration | *(d*_z_²)¹(d_xy_)^0^ | | *(d*_z_²)¹(d_xy_)^0^ | | *(d*_z_²)¹(d_xy_)¹ | |
| parameters | expt. | calcd | expt. | calcd | expt. | calcd |
| *d*(Fe-N(O))/ Å | 1.663 | 1.651 | 1.701 | 1.681 | 1.698 | 1.670 |
| Δ^Fe^_N2S2_/ Å | 0.50 | 0.49 | 0.54 | 0.50 | 0.83 | 0.82 |
| τ value | 0.25 | 0.20 | 0.15 | 0.19 | 0.21 | 0.21 |
| ∠Fe-N-O/ ° | 162.7 | 163.6 | 153.1 | 148.6 | 166.4 | 162.8 |
| ν(NO)/ cm^−1^ | 1779 | 1774 | 1667 | 1654 | 1624 | 1628 |
|  |  | |  | |  | |
| E-F count | **{Fe(NO)_2_}^9^** | | **{Fe(NO)_2_}^10^** | | **{Fe(NO)_2_}^10^** | |
| parameters | expt. | calcd | expt. | calcd | expt. | calcd |
| *d*(Fe2-N4(O2))  *d*(Fe2-N5(O3))/Å | 1.666  1.662 | 1.666  1.656 | 1.650  1.654 | 1.647  1.642 | 1.642  1.657 | 1.640  1.640 |
| *d*(Fe2-S1)  *d*(Fe2-S2)/ Å | 2.256  2.242 | 2.247  2.235 | 2.315  2.328 | 2.322  2.315 | 2.369  2.348 | 2.365  2.350 |
| τ value | 0.88 | 0.81 | 0.89 | 0.87 | 0.89 | 0.89 |
| ∠Fe2-N4-O2  ∠Fe2-N5-O3/ ° | 160.3  176.5 | 162.9  179 | 170.4  167.4 | 171.9  167.1 | 171.9  168.3 | 169.7  167.6 |
| ν(N4O2)  ν(N5O3)/ cm^−1^ | 1809  1742 | 1812  1717 | 1701  1640 | 1751  1639 | 1655  1607 | 1694  1604 |

*^*^*Hinge angle between Fe1(S)2 and Fe2(S)2 planes

**Table S11.** Mulliken charge and spin population of **[^L3^Fe_2_(NO)_3_]^+/0/−^** series calculated from single-point calculation using the TPSSh functional. Identification of atoms given in Figure 7 (see main manuscript).

| *Mulliken charge* | MNIU*^a^* | DNIU*^a^* | Fe1 | (NO)1 | Fe2 | (NO)2 | (NO)2′ | S1 | S2 |
| --- | --- | --- | --- | --- | --- | --- | --- | --- | --- |
| **[^L3^Fe_2_(NO)_3_]^+^** | 0.88 | 0.12 | 1.20 | −0.09 | 1.01 | −0.27 | −0.21 | −0.39 | −0.42 |
| **[^L3^Fe_2_(NO)_3_]^0^** | 0.61 | −0.61 | 2.10 | −0.28 | 1.39 | −0.45 | −0.56 | −0.95 | −1.05 |
| **[^L3^Fe_2_(NO)_3_]^−^** | −0.15 | −0.85 | 2.26 | −0.77 | 0.72 | −0.48 | −0.52 | −0.42 | −0.72 |
| *Mulliken spin population* |  |  |  |  |  |  |  |  |  |
| **[^L3^Fe_2_(NO)_3_]^+^** | 0.07 | −0.07 | 0.35 | −0.26 | 1.11 | −0.58 | −0.60 | 0.002 | 0.004 |
| **[^L3^Fe_2_(NO)_3_]^0^** | 0.87 | 0.11 | 1.26 | −0.37 | −0.03 | 0.11 | −0.00 | 0.03 | 0.03 |
| **[^L3^Fe_2_(NO)_3_]^−^** | 1.85 | 0.15 | 2.75 | −1.06 | 0.09 | 0.02 | −0.07 | 0.13 | 0.11 |

*^a^the sums of charges and spins of sulfur 1 and sulfur 2 are split between MNIU and DNIU.*

| **[^L3^Fe_2_(NO)_3_]^+^** | |
| --- | --- |
| Alpha orbital | Beta orbital |
| 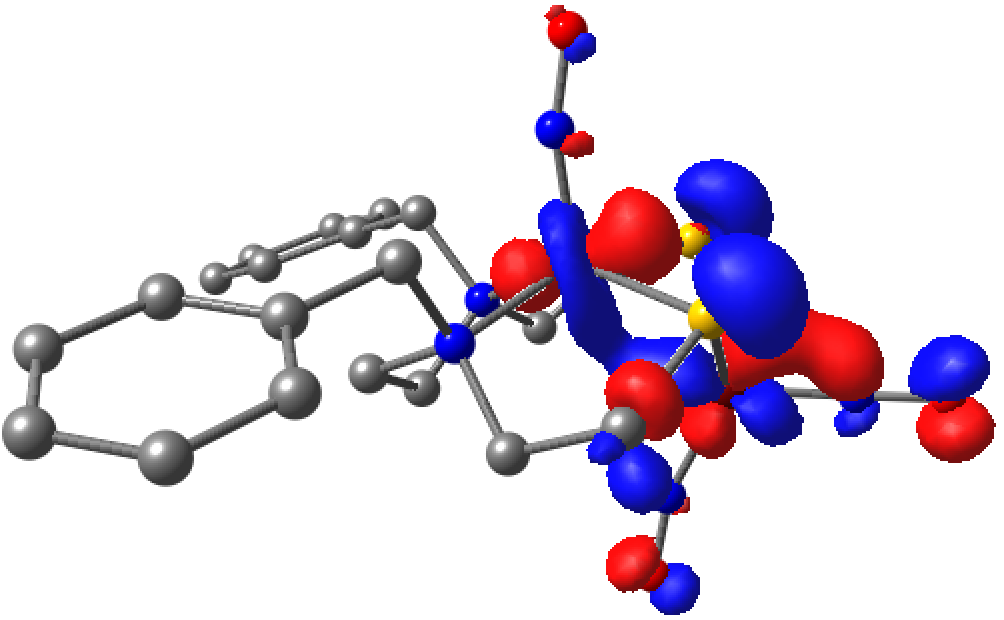  HOMO-2 | 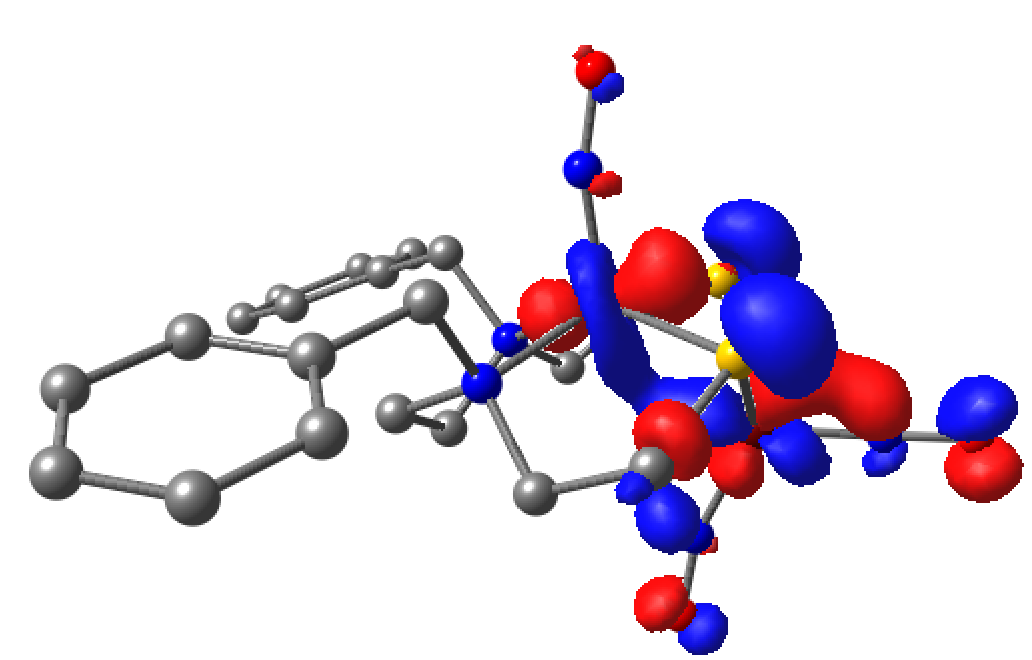  HOMO-2 |
| 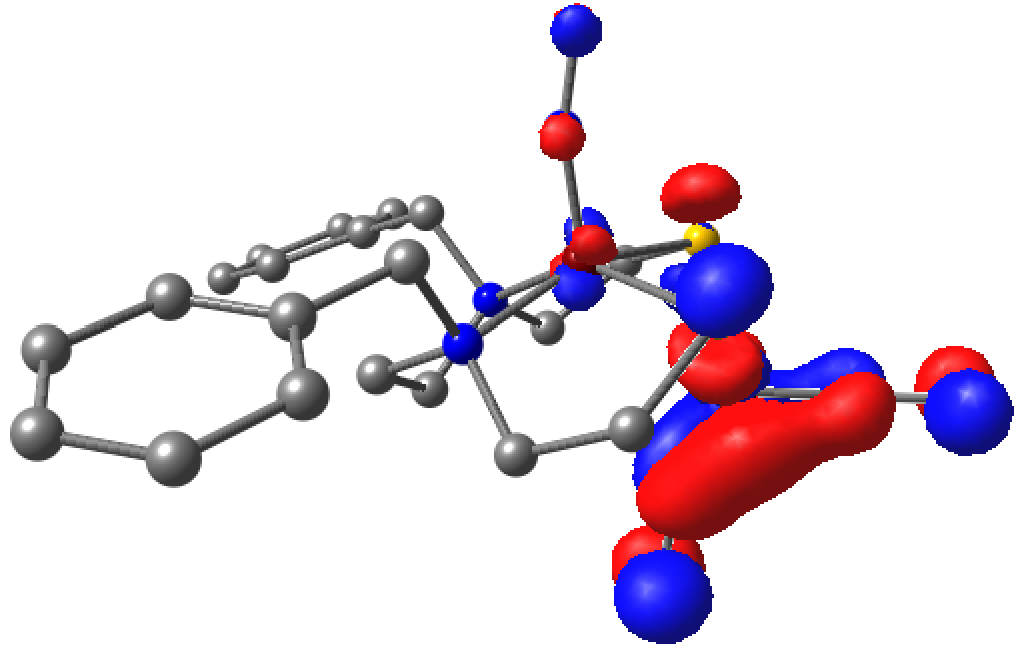  HOMO-1 | 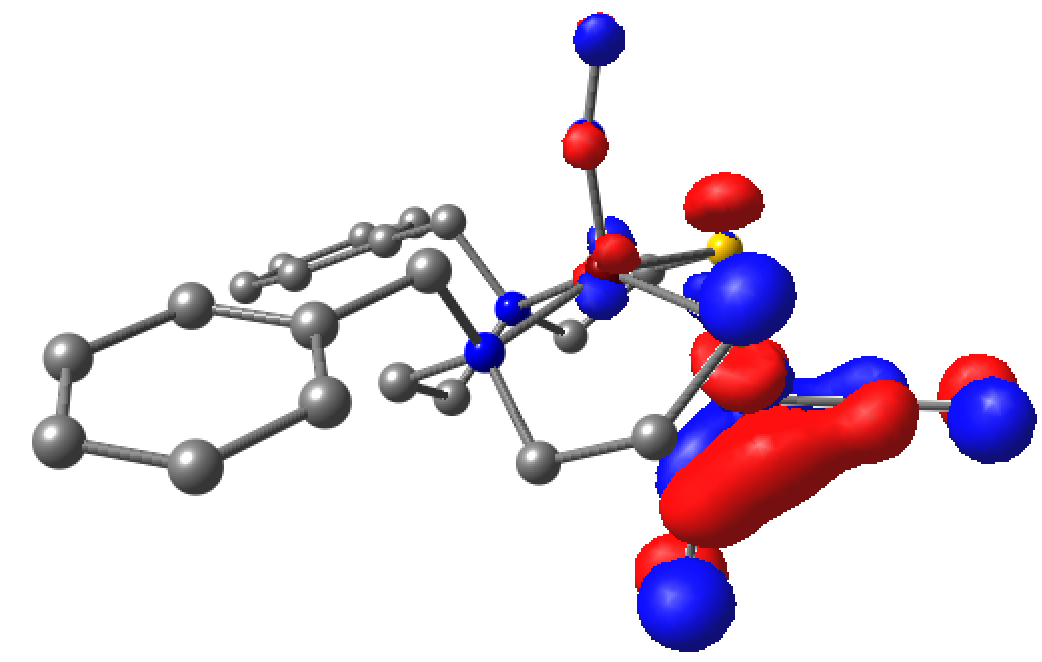  HOMO-1 |
| 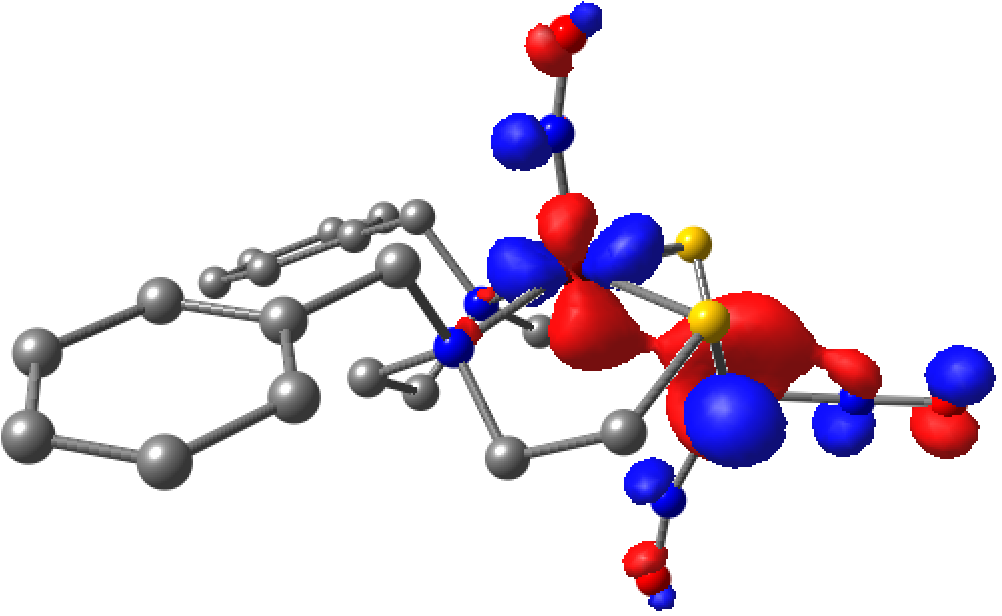  HOMO | 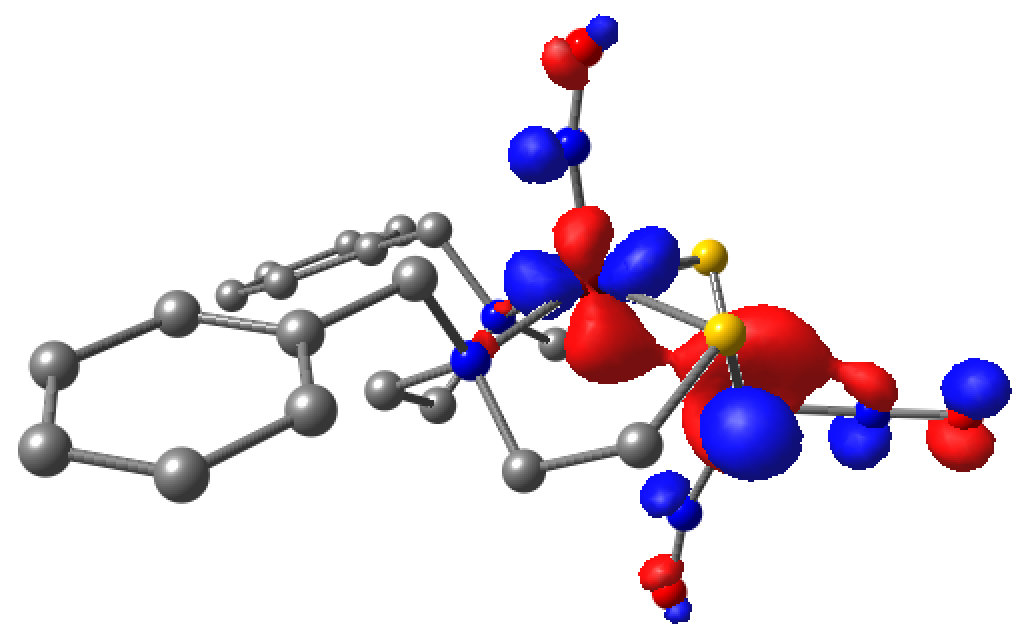  HOMO |
| 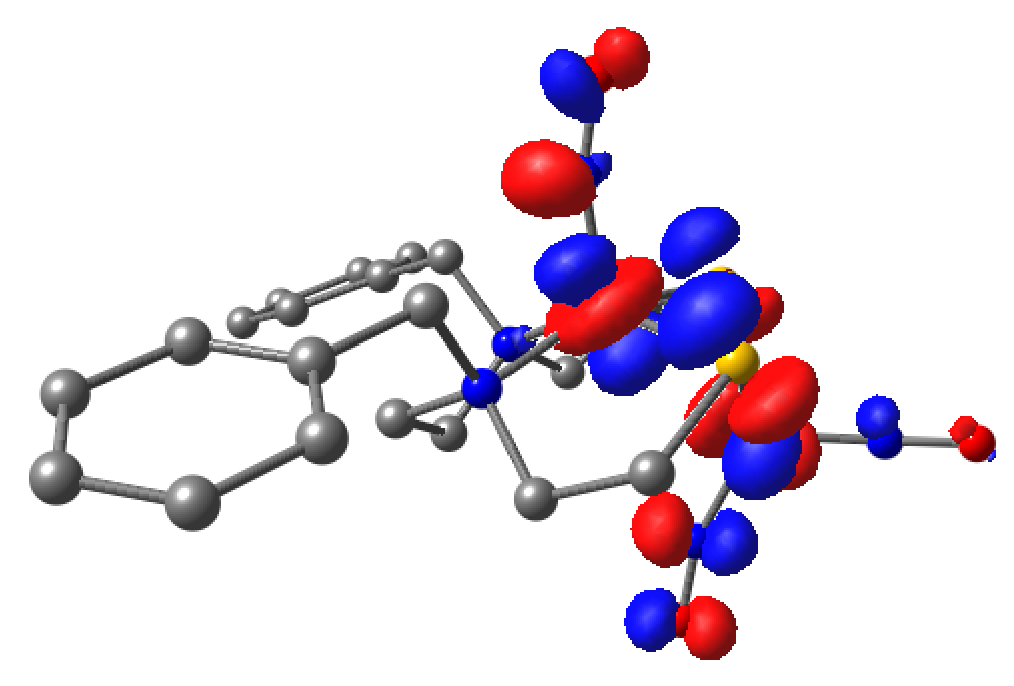  LUMO | 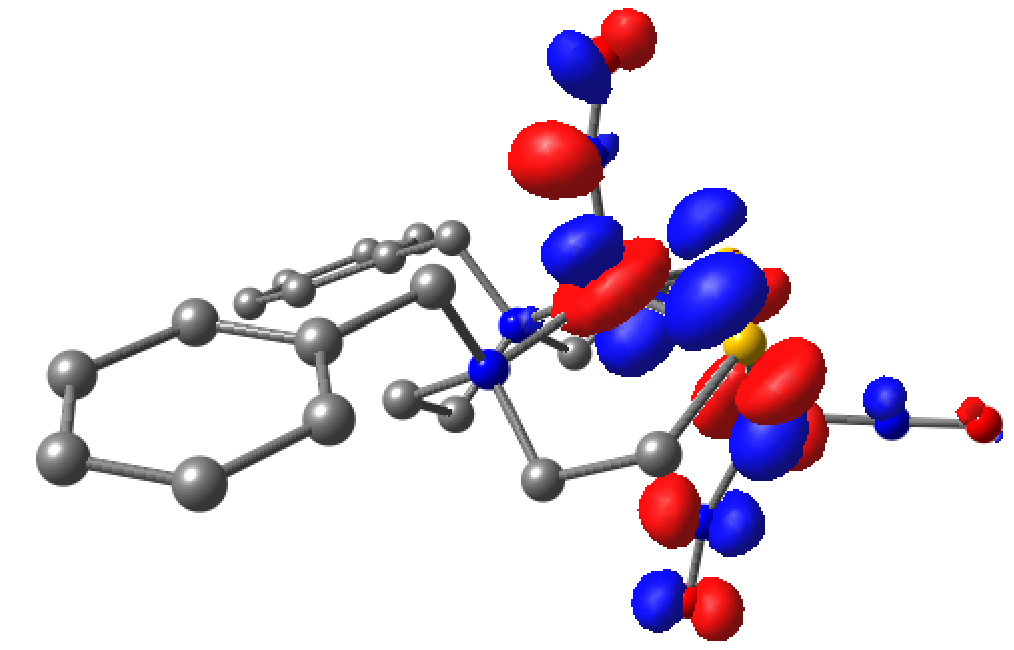  LUMO |
| 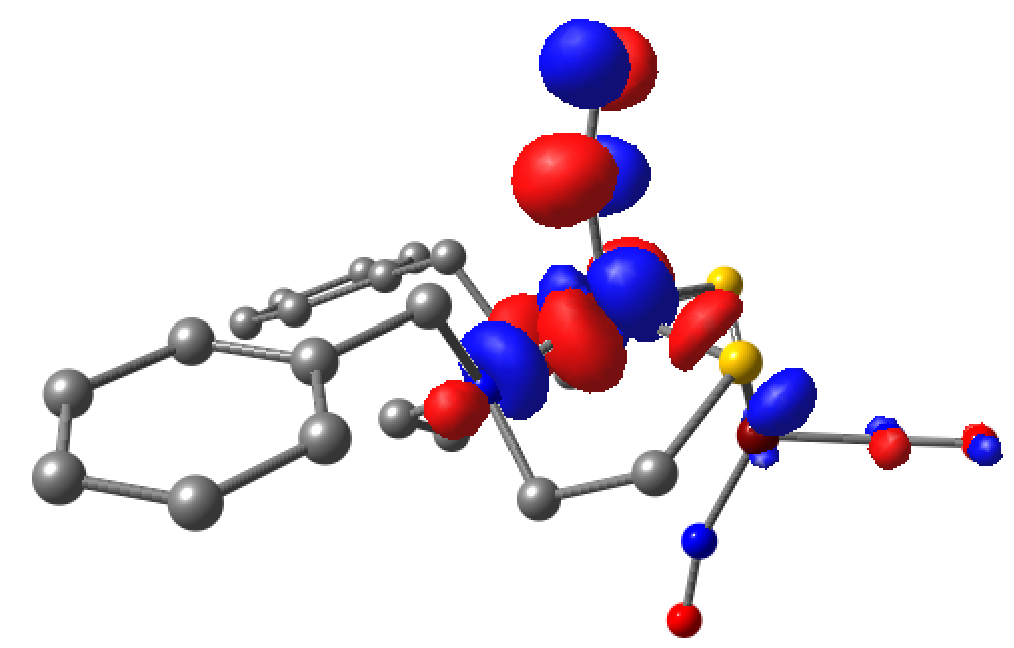  LUMO+1 | 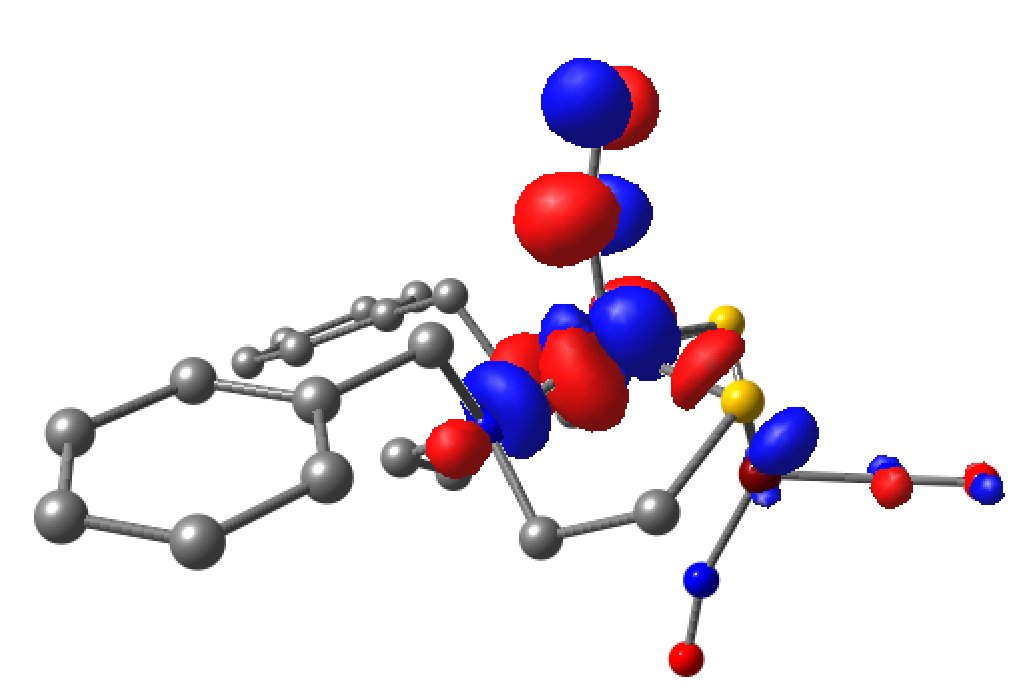  LUMO+1 |
| 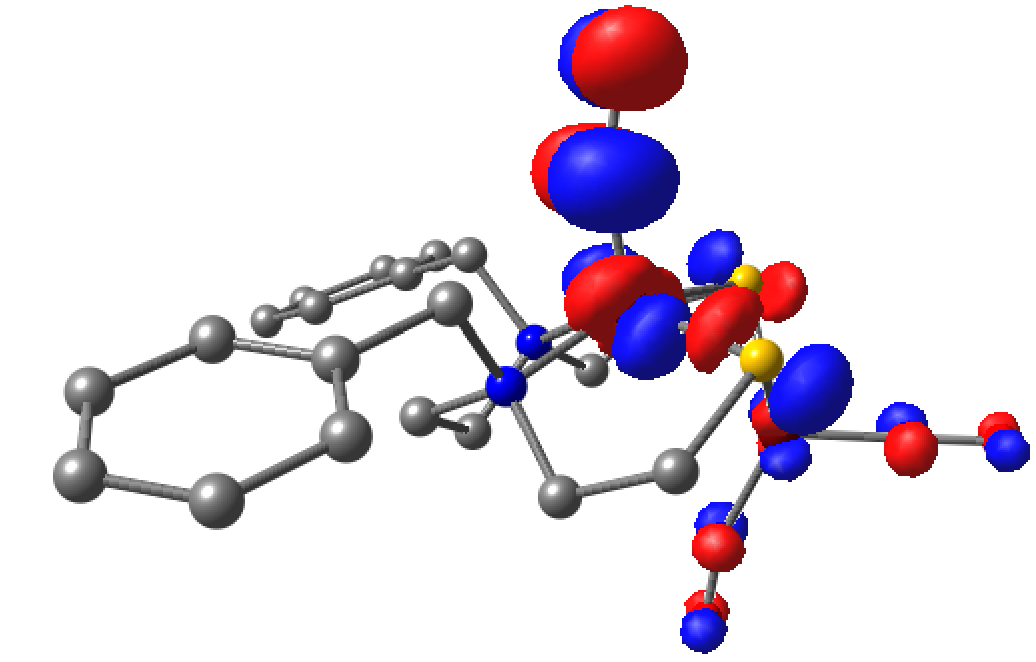  LUMO+2 | 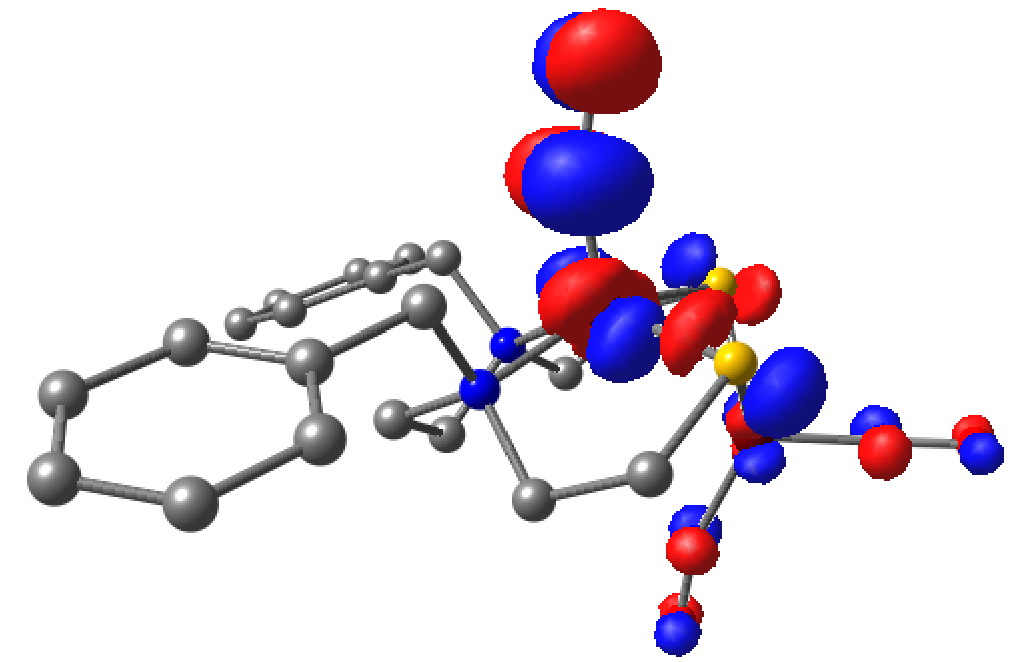  LUMO+2 |
| 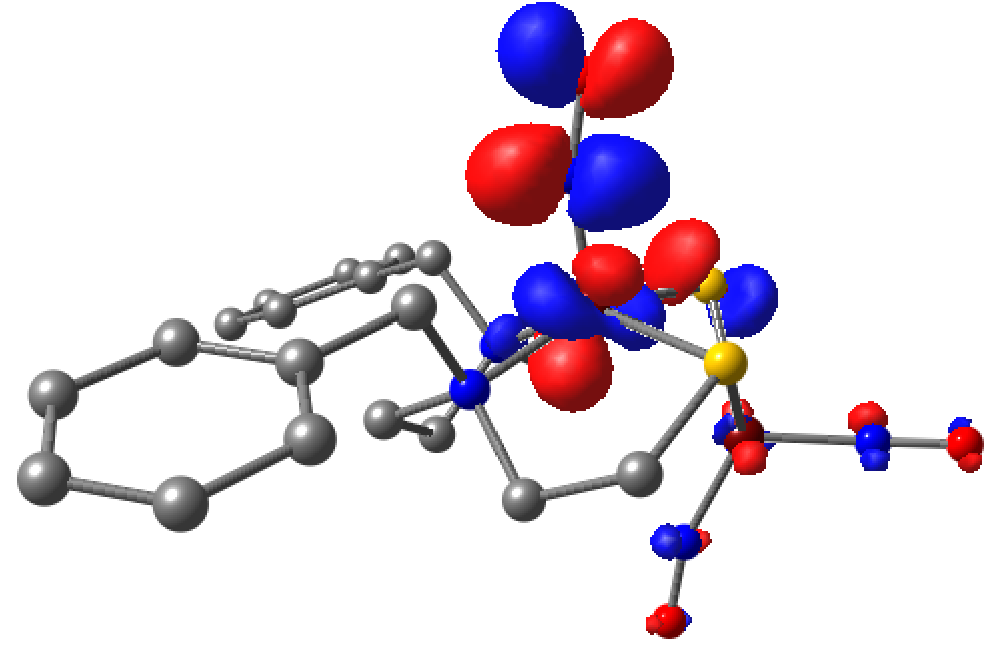  LUMO+3 | 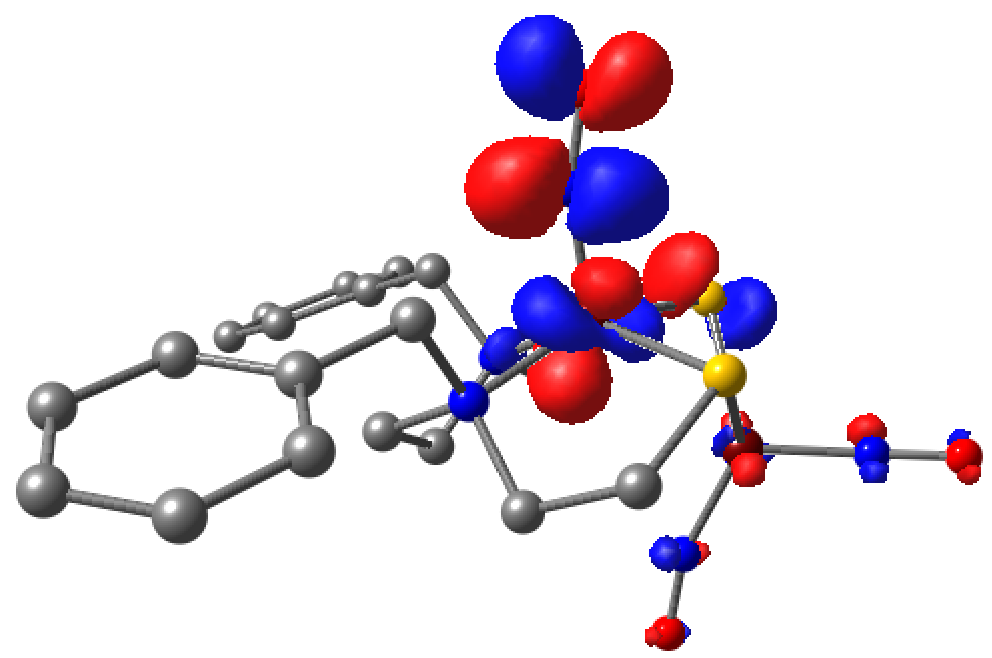  LUMO+3 |

**Table S12.** Alpha and Beta orbitals of **[^L3^Fe_2_(NO)_3_]^+^**, calculated at unrestricted TPSS/6-311++G(d,p) level of theory.

| **[^L3^Fe_2_(NO)_3_]^0^**  **Table S13.** Alpha and Beta orbitals of **[^L3^Fe_2_(NO)_3_]^0^**, calculated at unrestricted TPSS/6-311++G(d,p) level of theory.  . | |  |
| --- | --- | --- |
| Alpha orbital | Beta orbital |  |
| 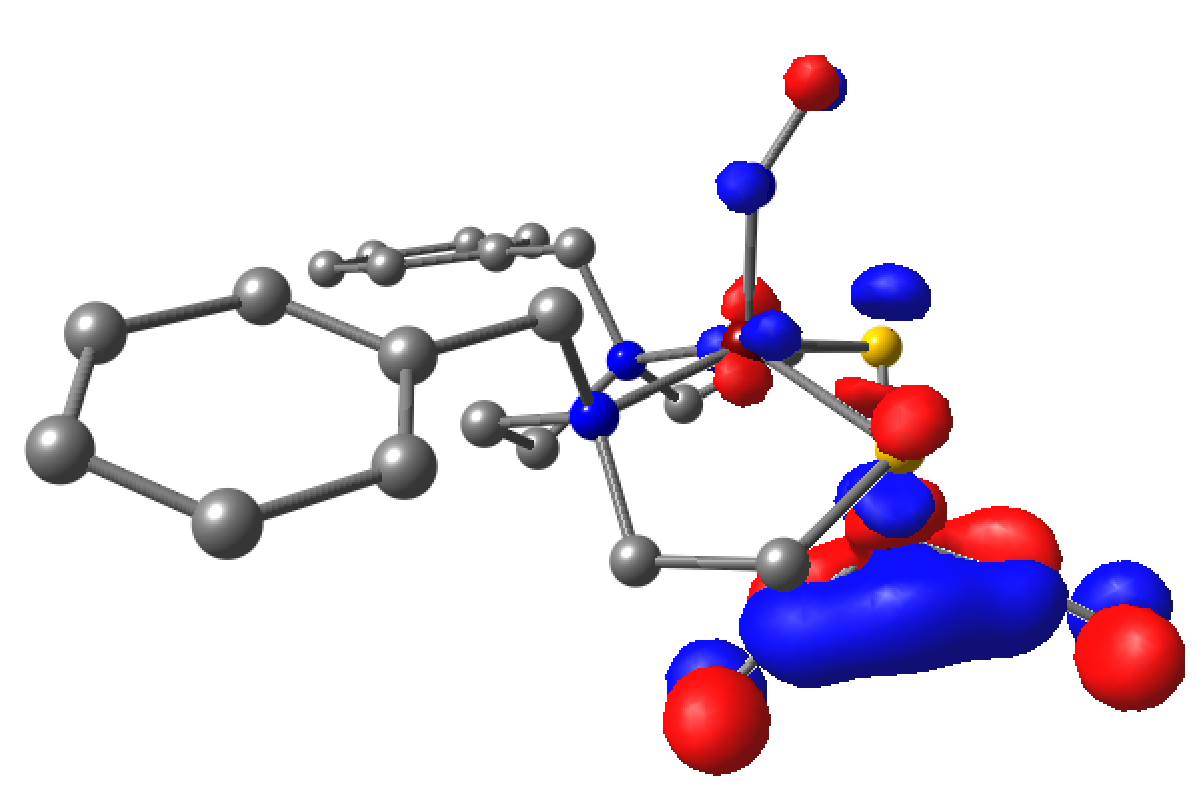  HOMO-2 | 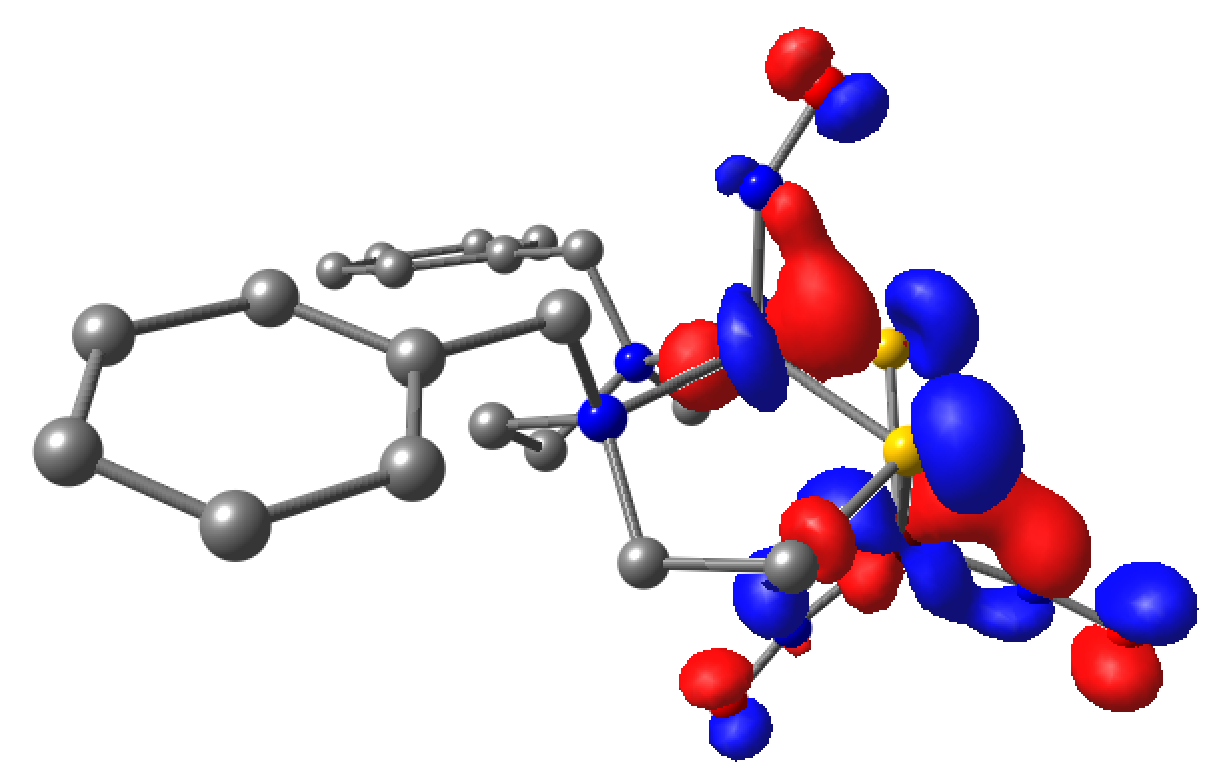  HOMO-2 |  |
| 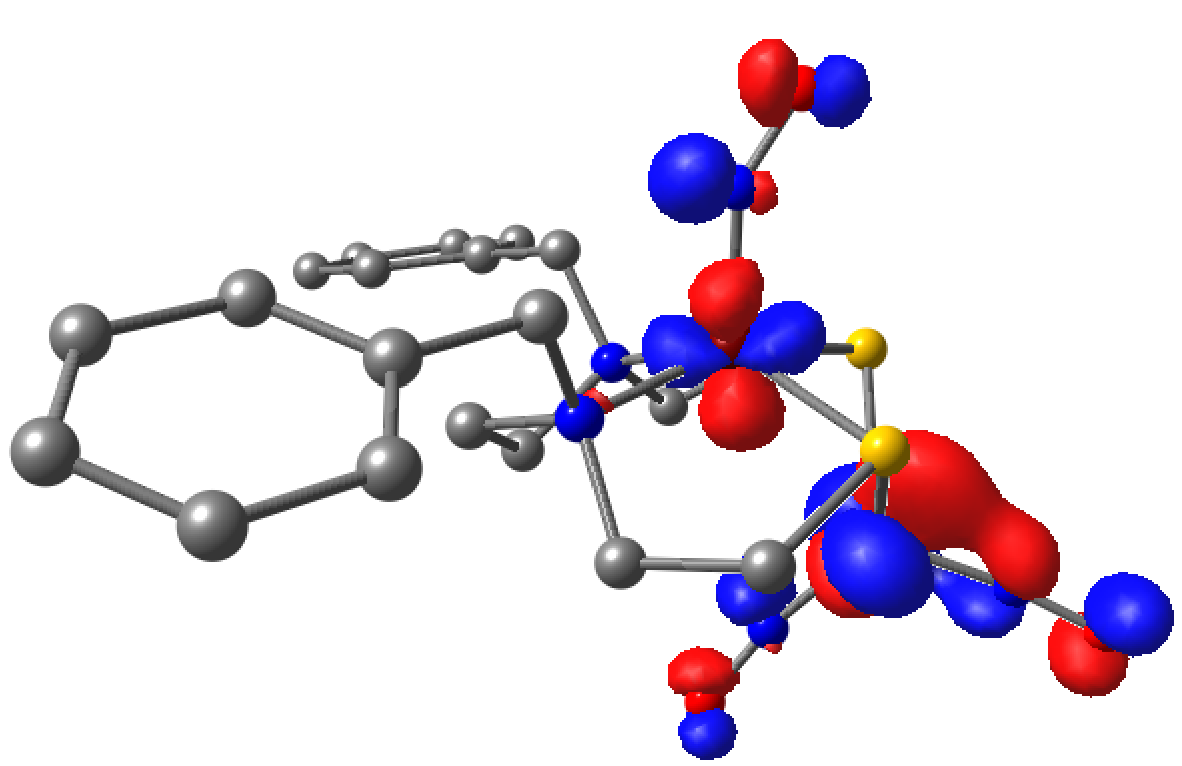  HOMO-1 | 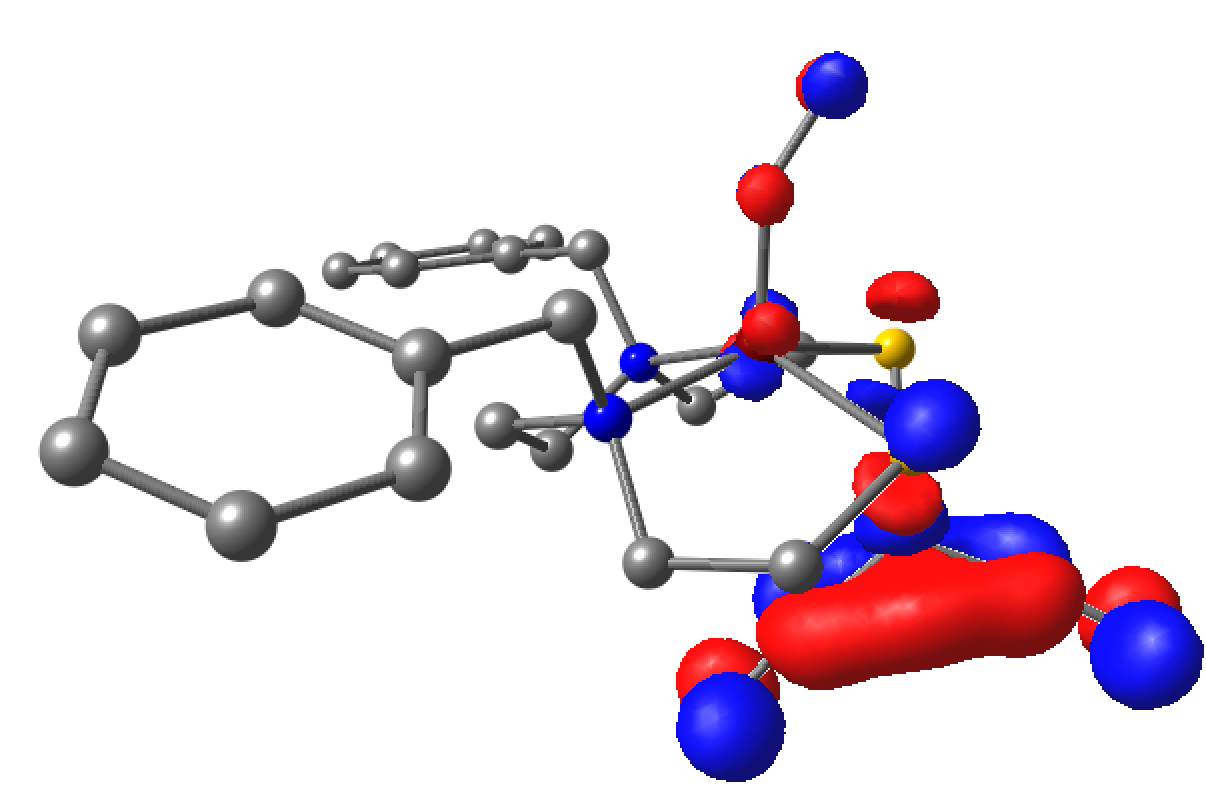  HOMO-1 |  |
| 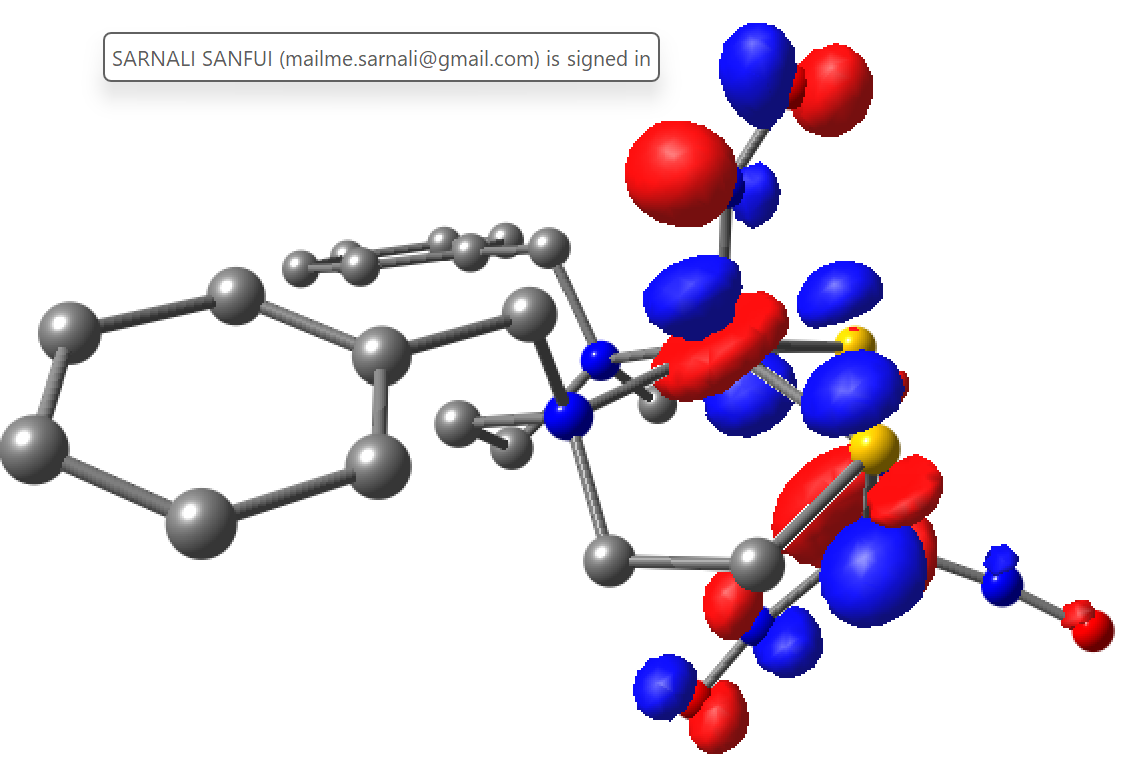  HOMO | 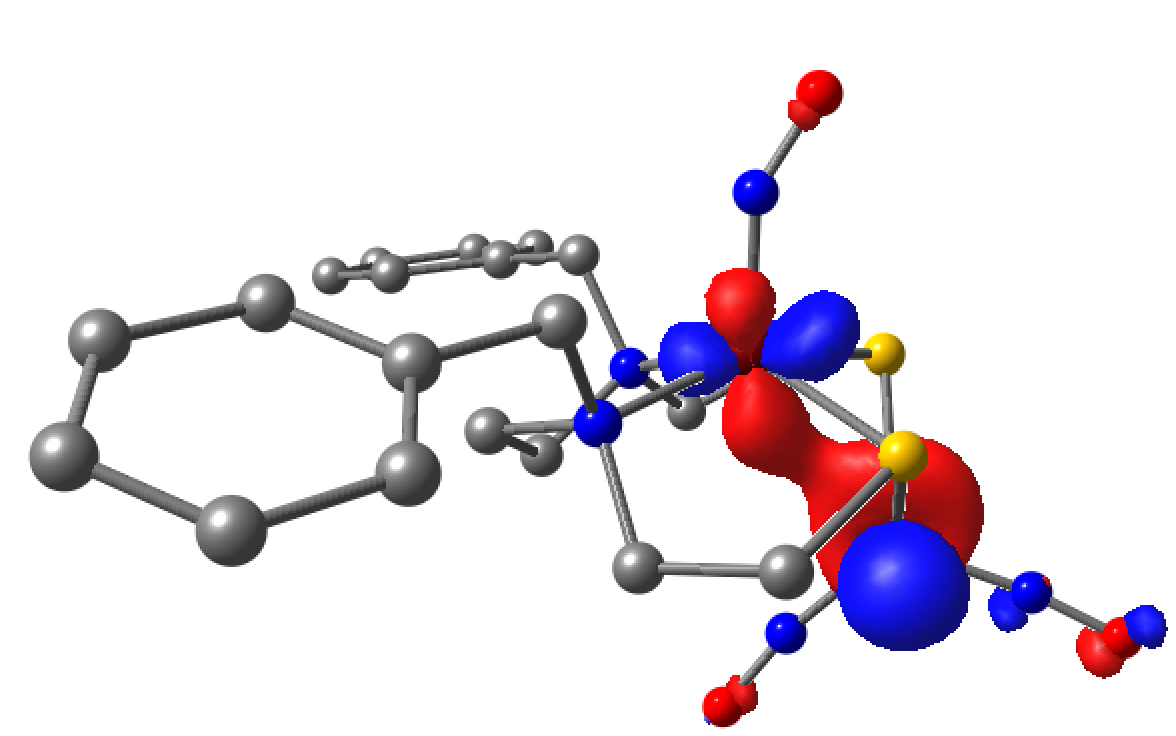  HOMO |  |
| 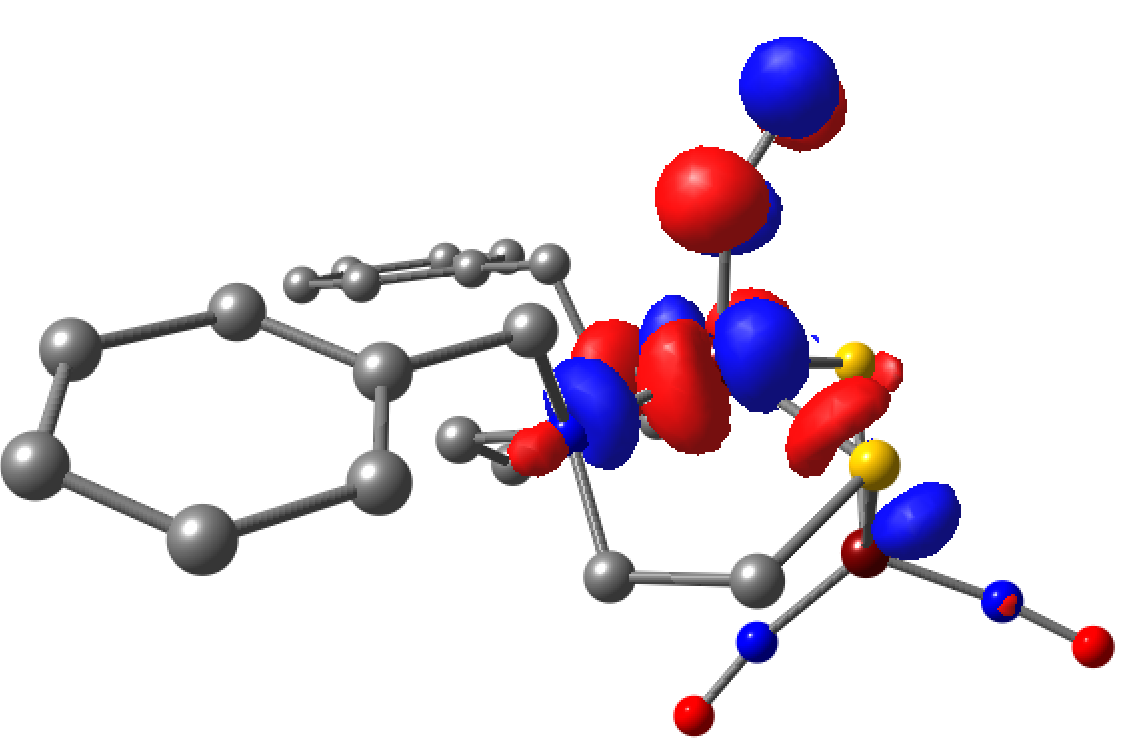  LUMO | 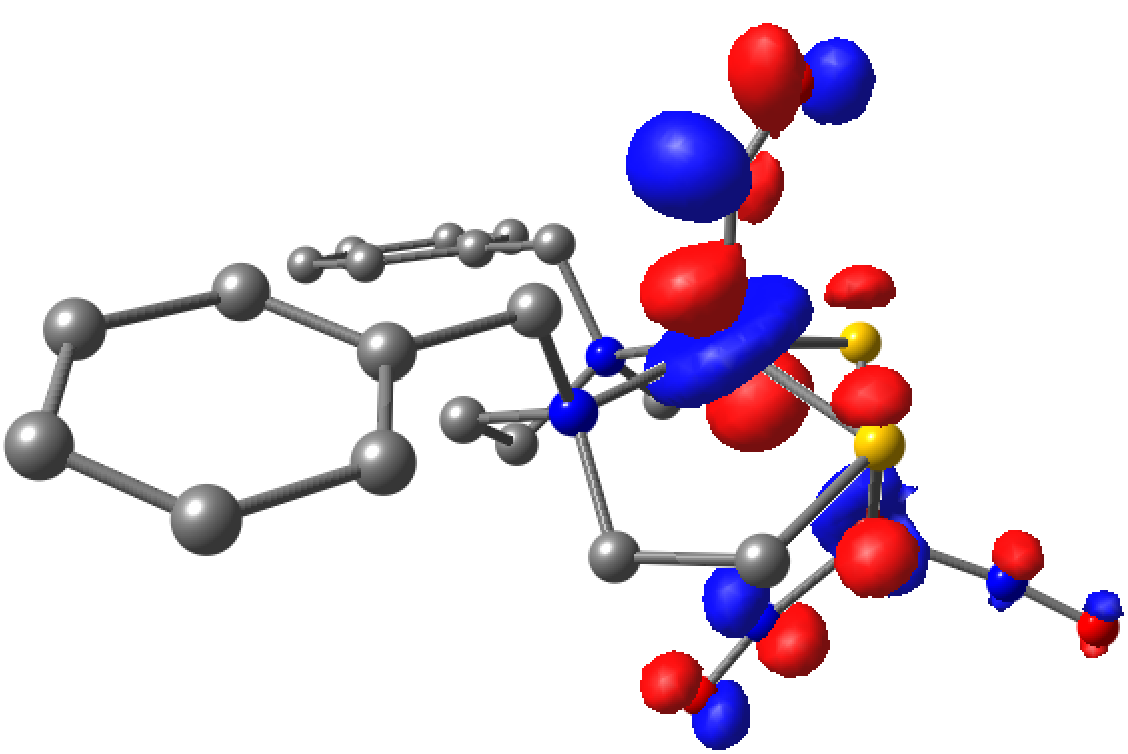  LUMO |  |
| 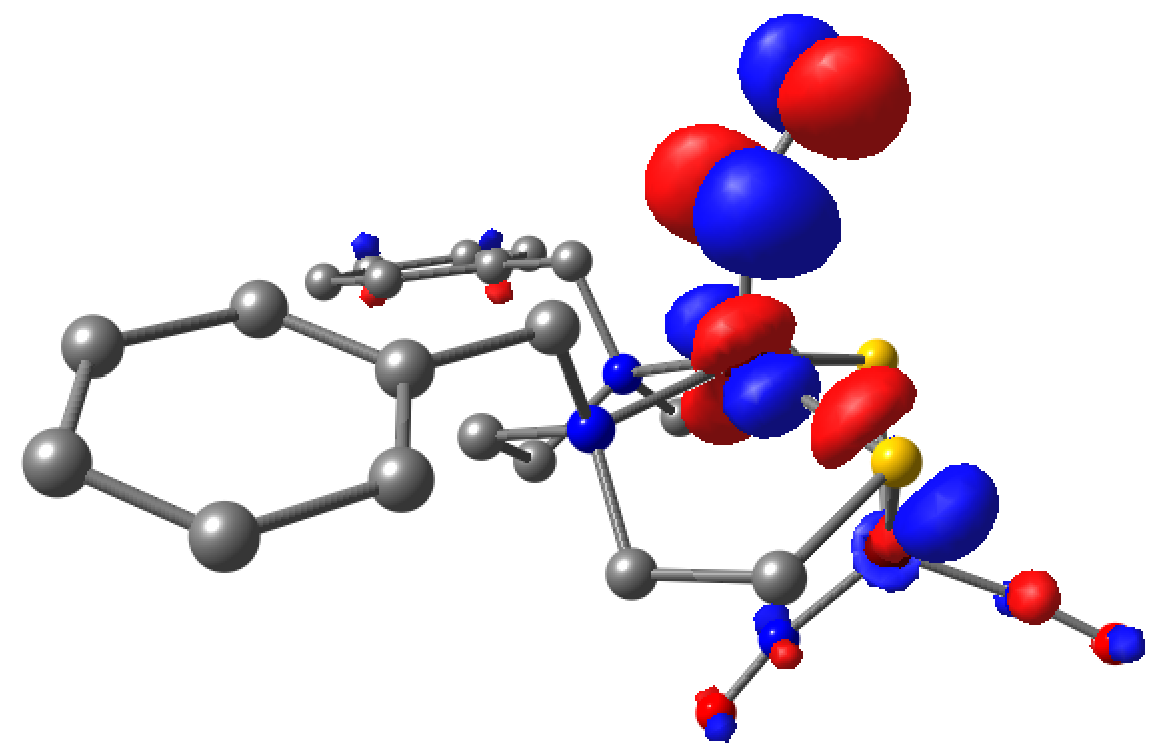  LUMO+1 | 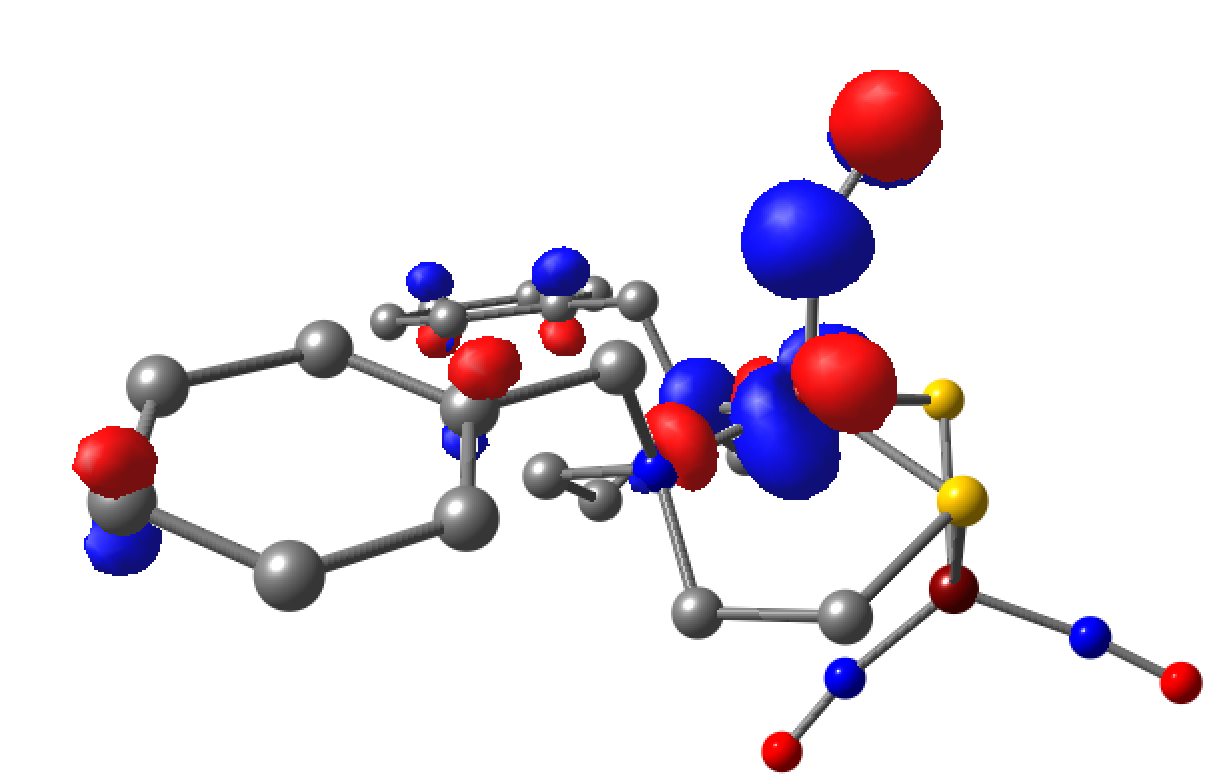  LUMO+1 |  |
| 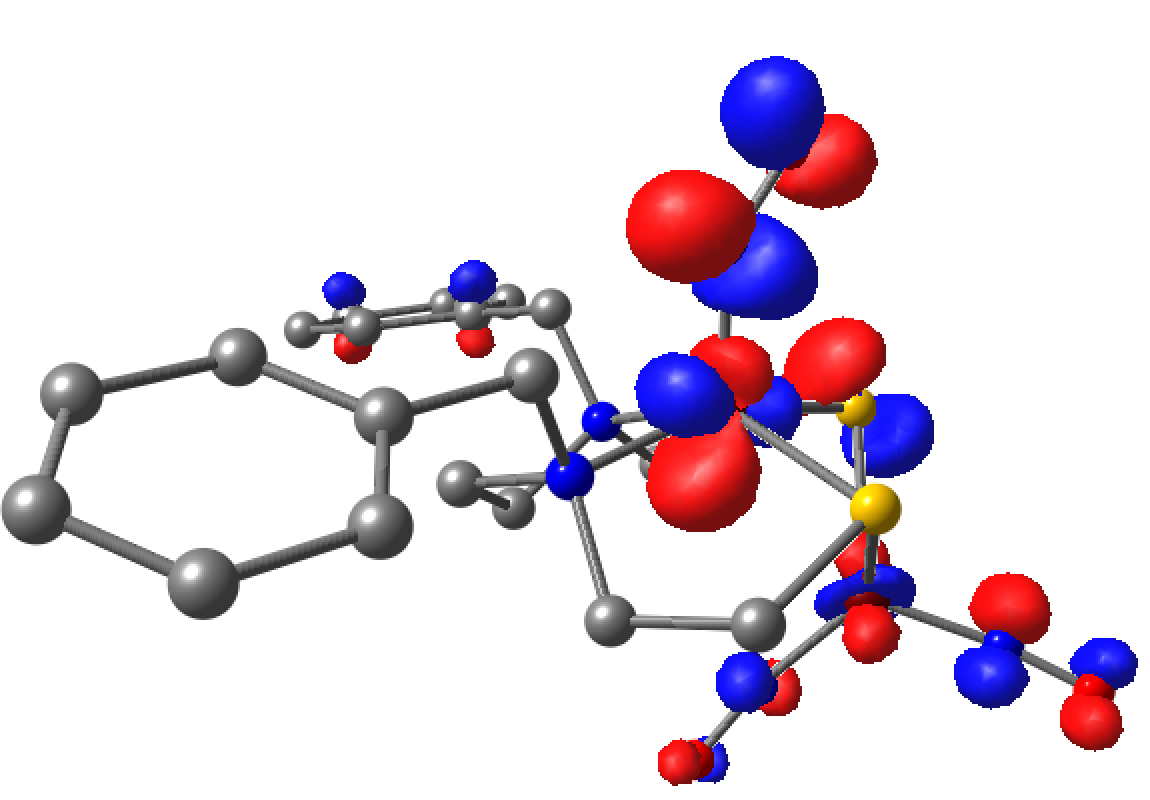  LUMO+2 | 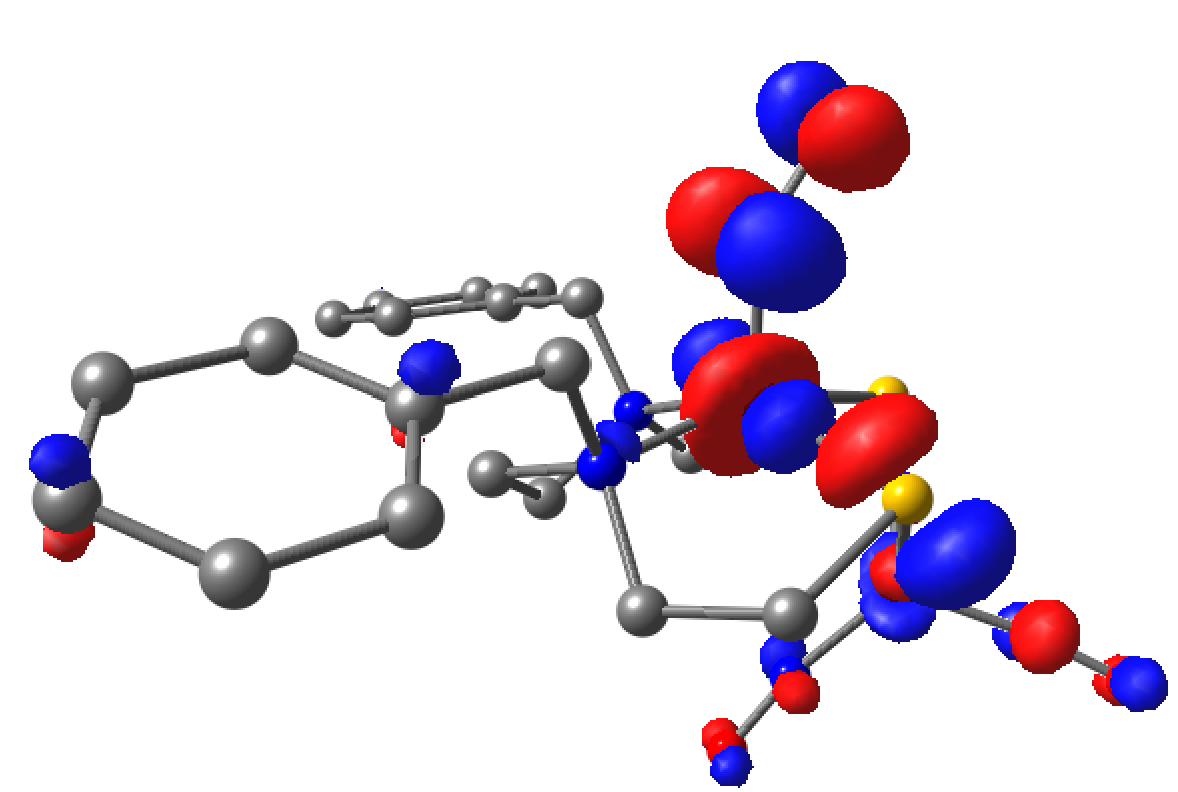  LUMO+2 |  |
| 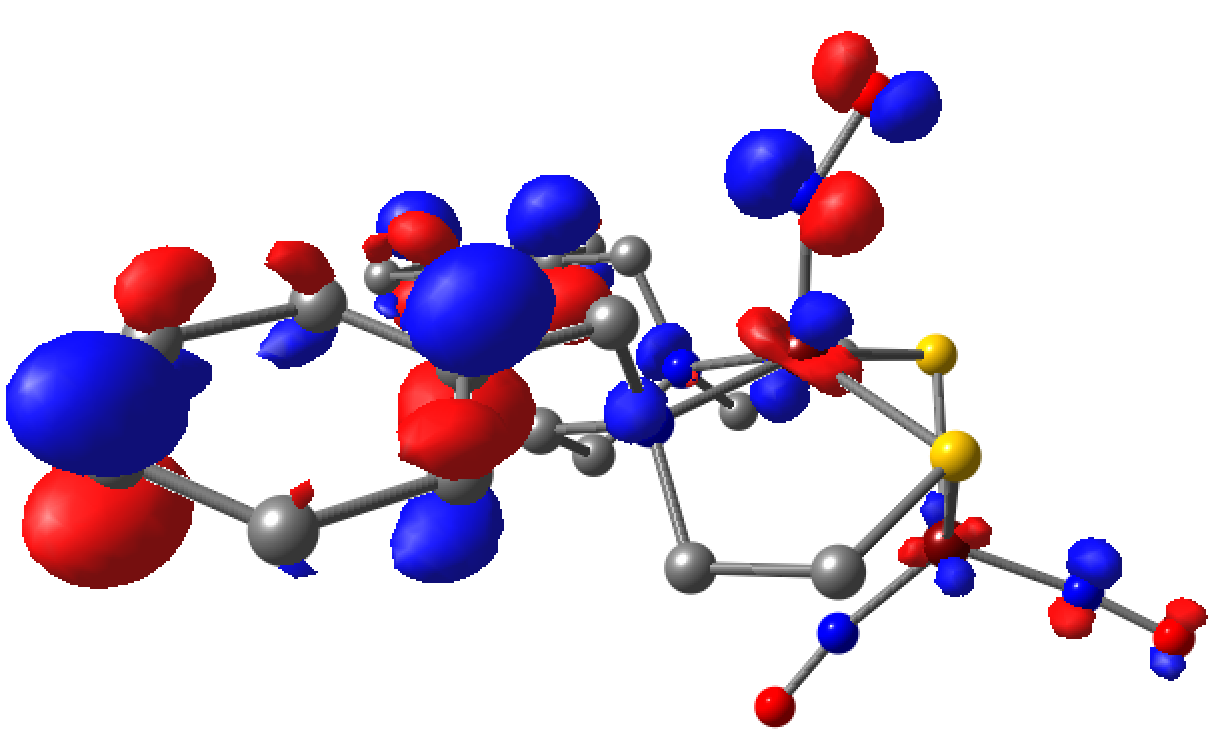  LUMO+3 | 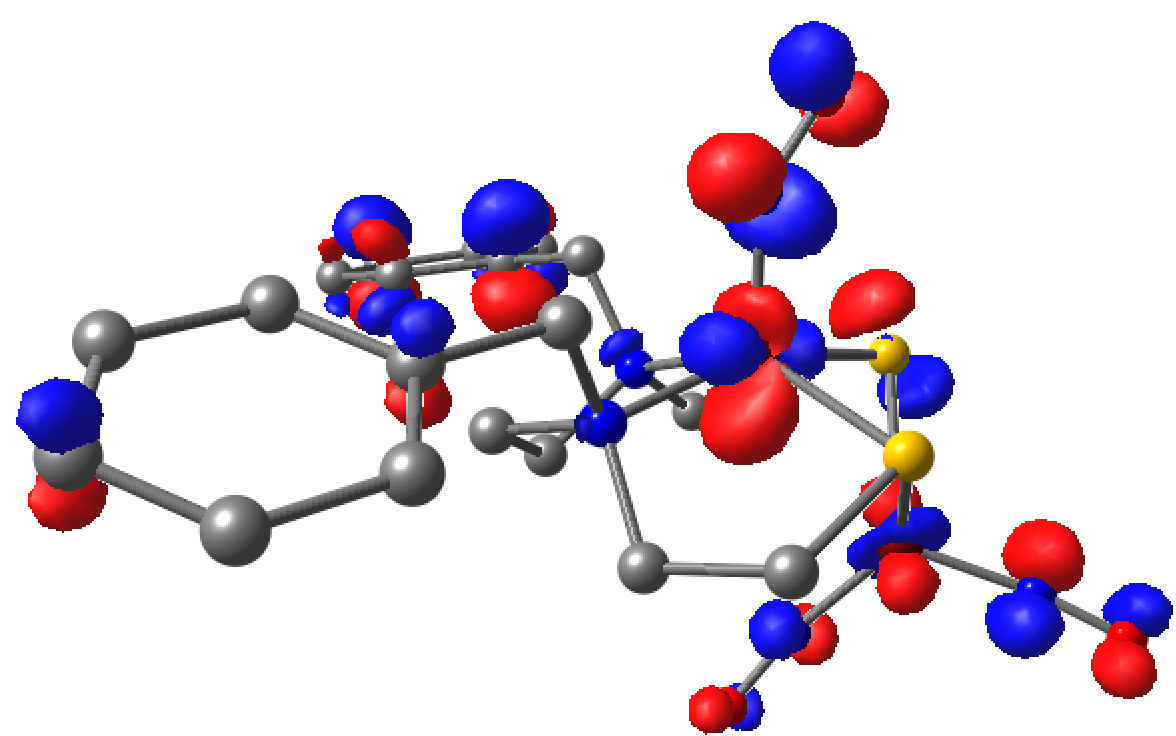  LUMO+3 |  |
| **[^L3^Fe_2_(NO)_3_]^−^** | | |
| Alpha orbital | | Beta orbital |
| 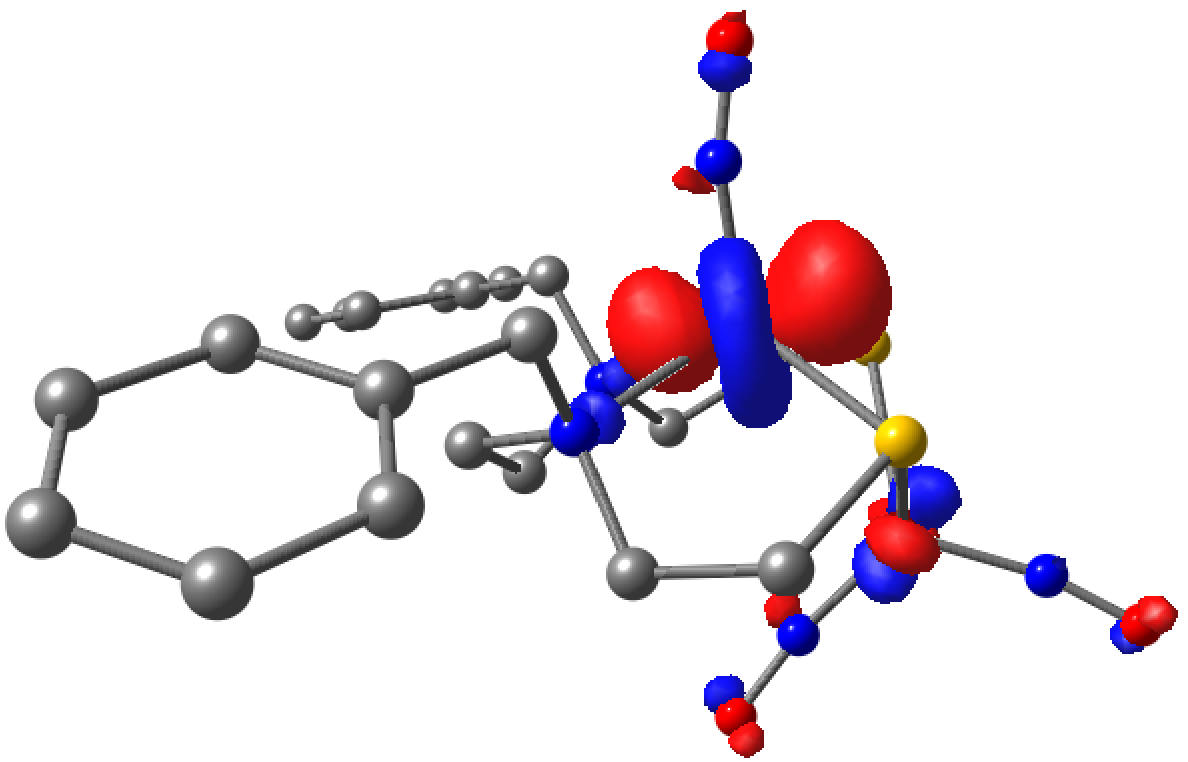  HOMO-2 | | 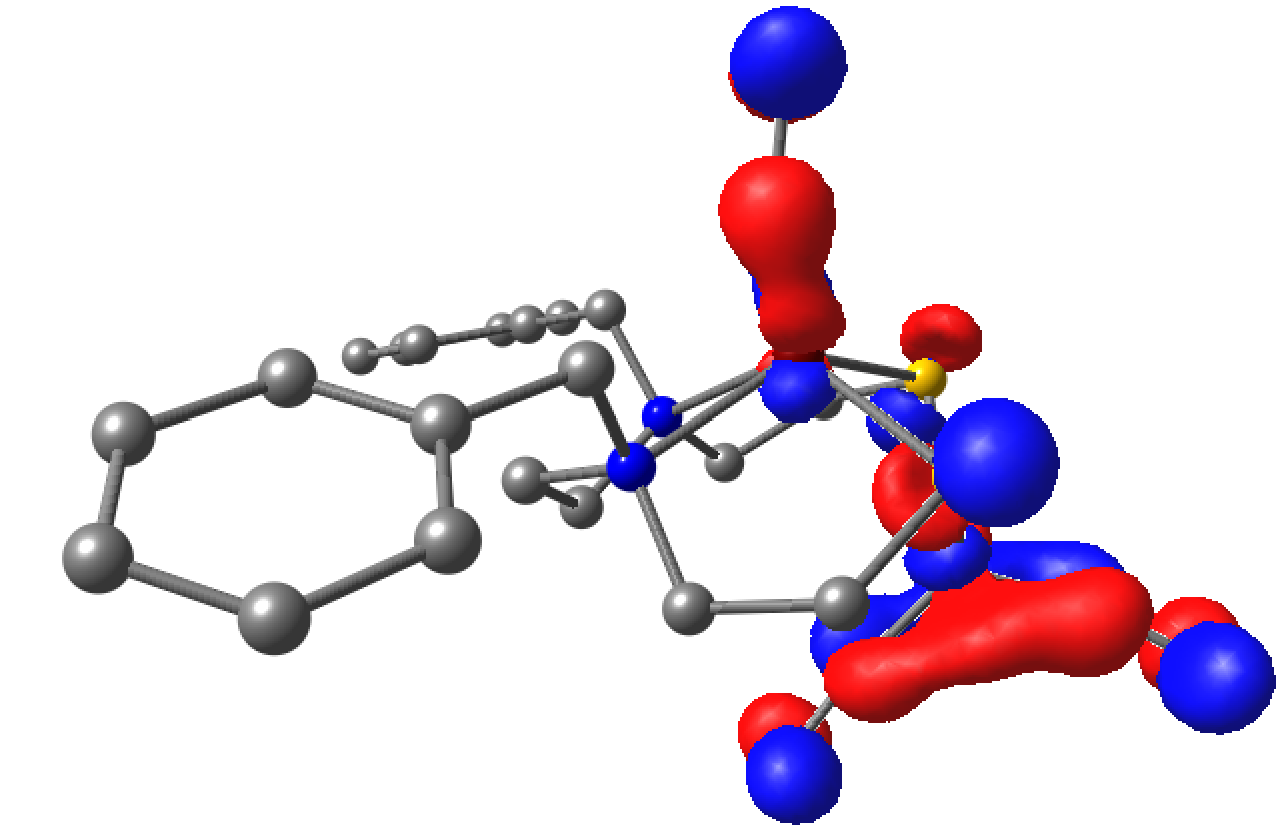  HOMO-2 |
| 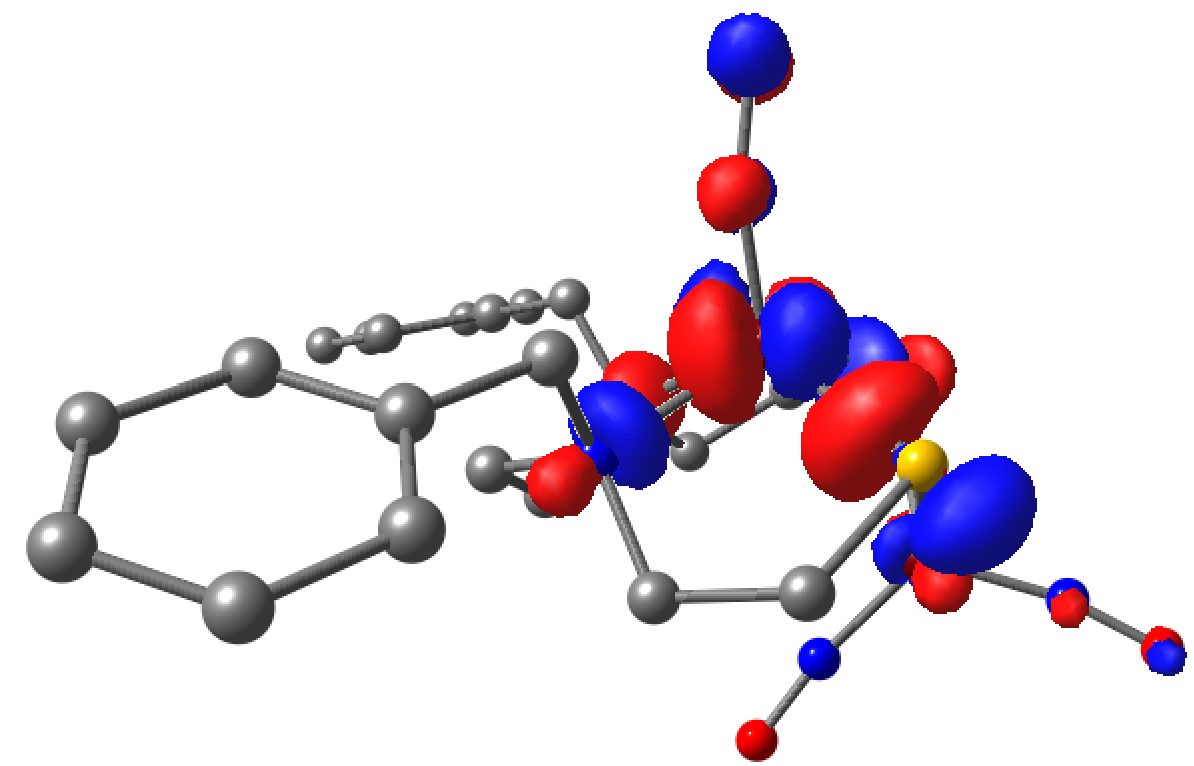  HOMO-1 | | 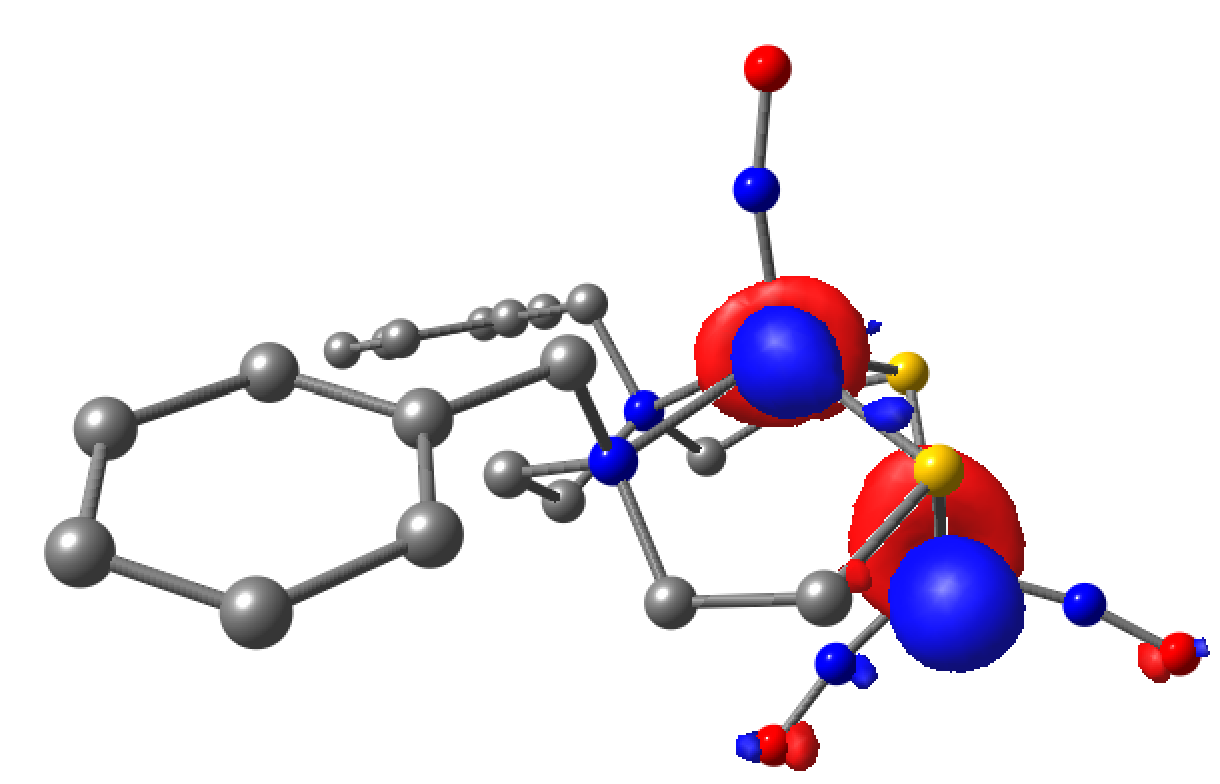  HOMO-1 |
| 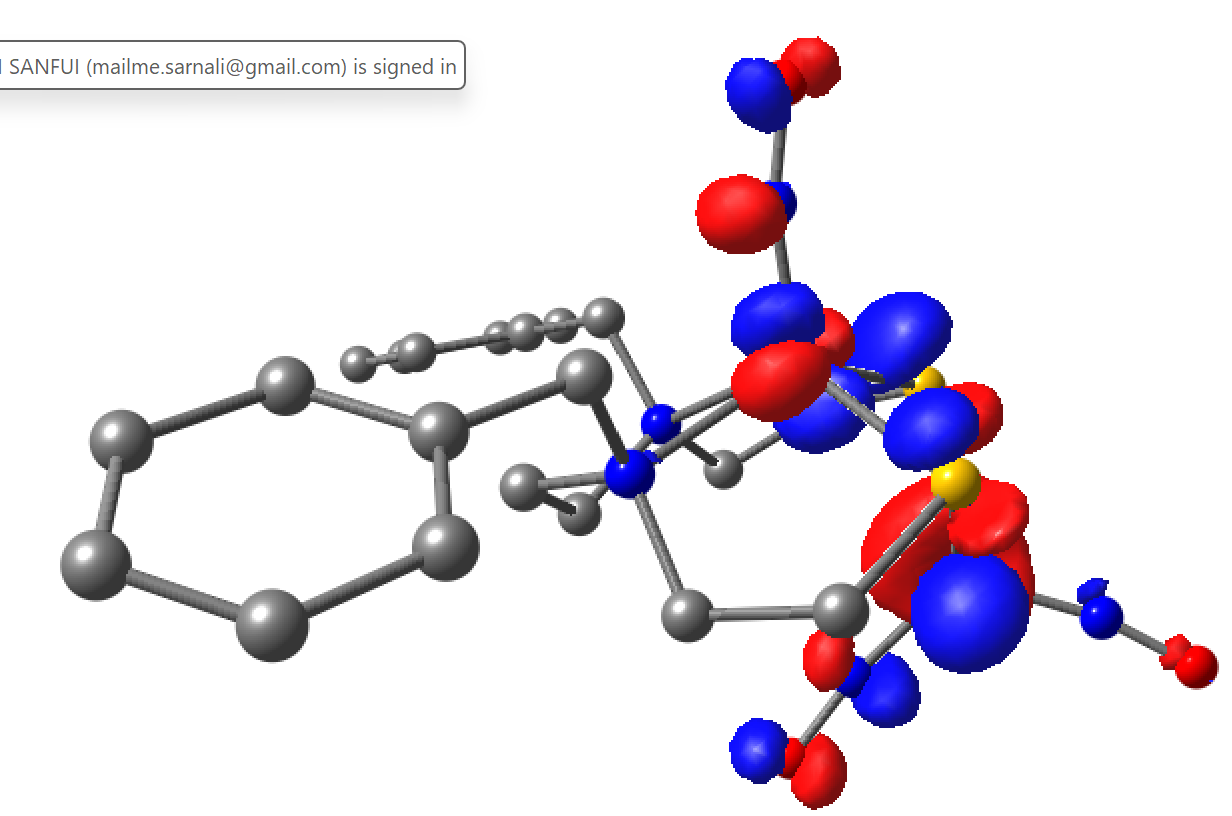  HOMO | | 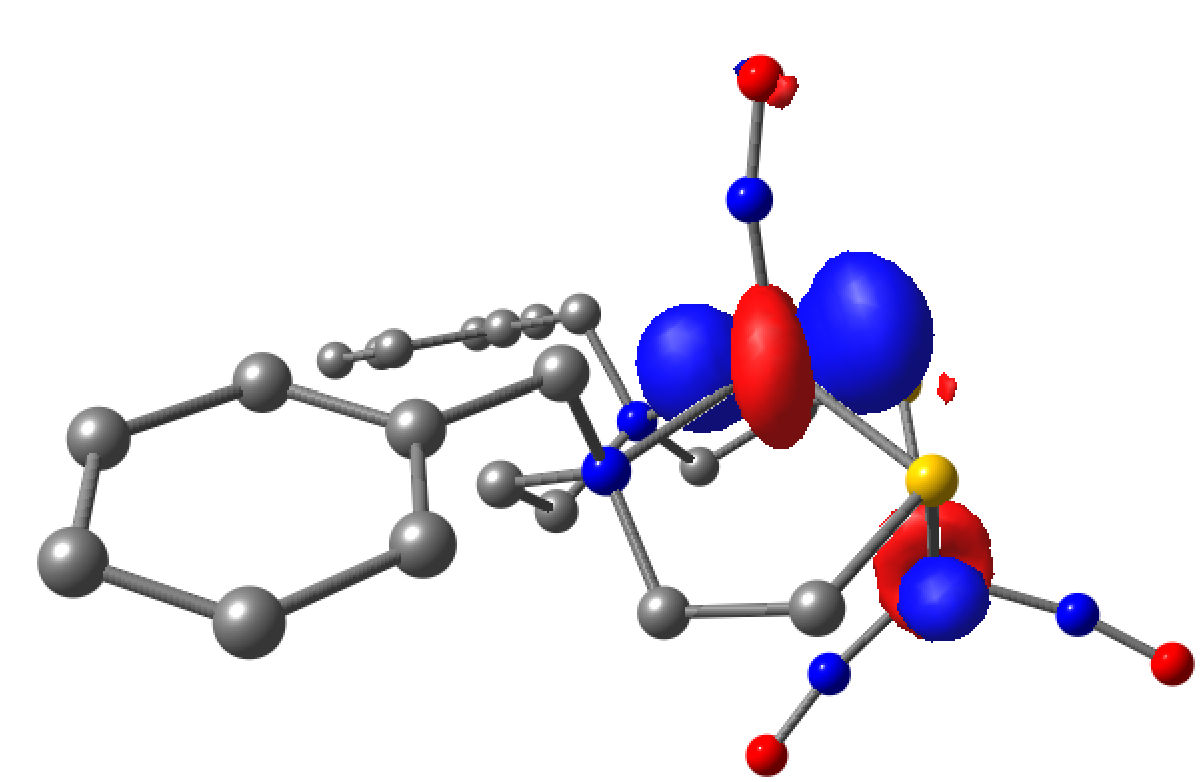  HOMO |
| 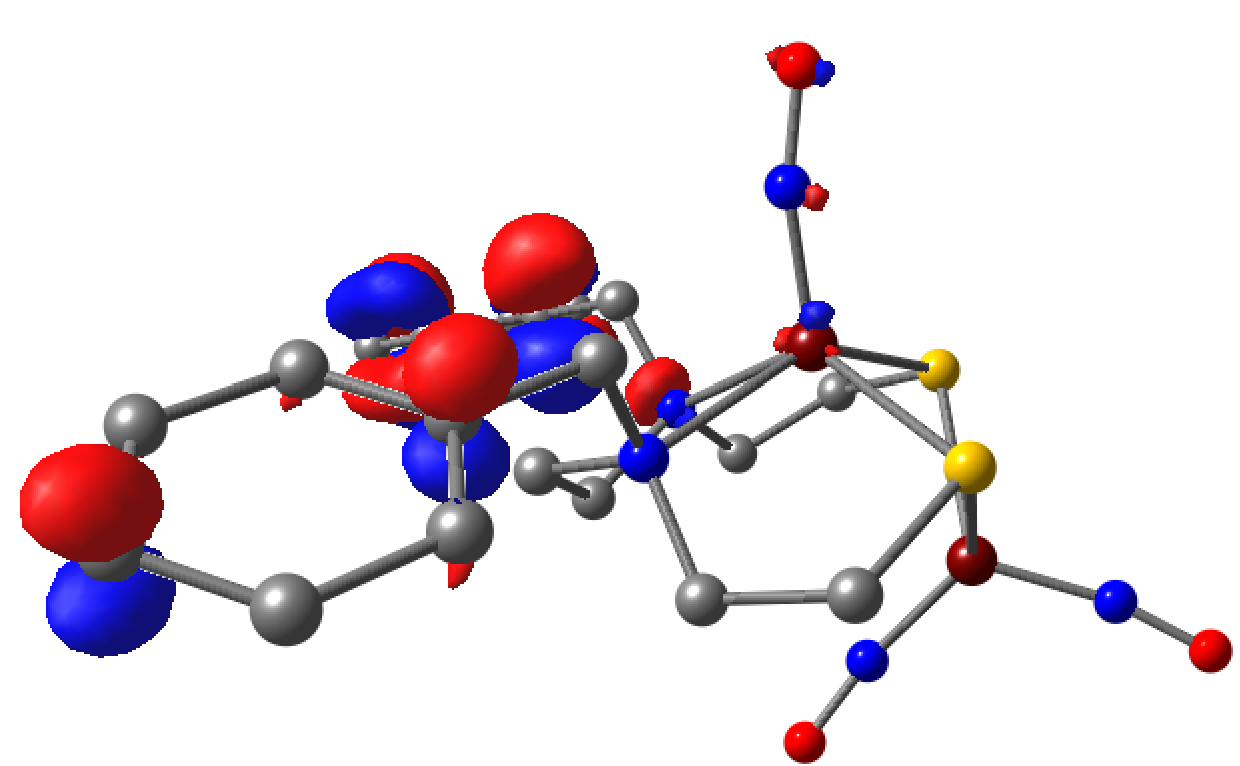  LUMO | | 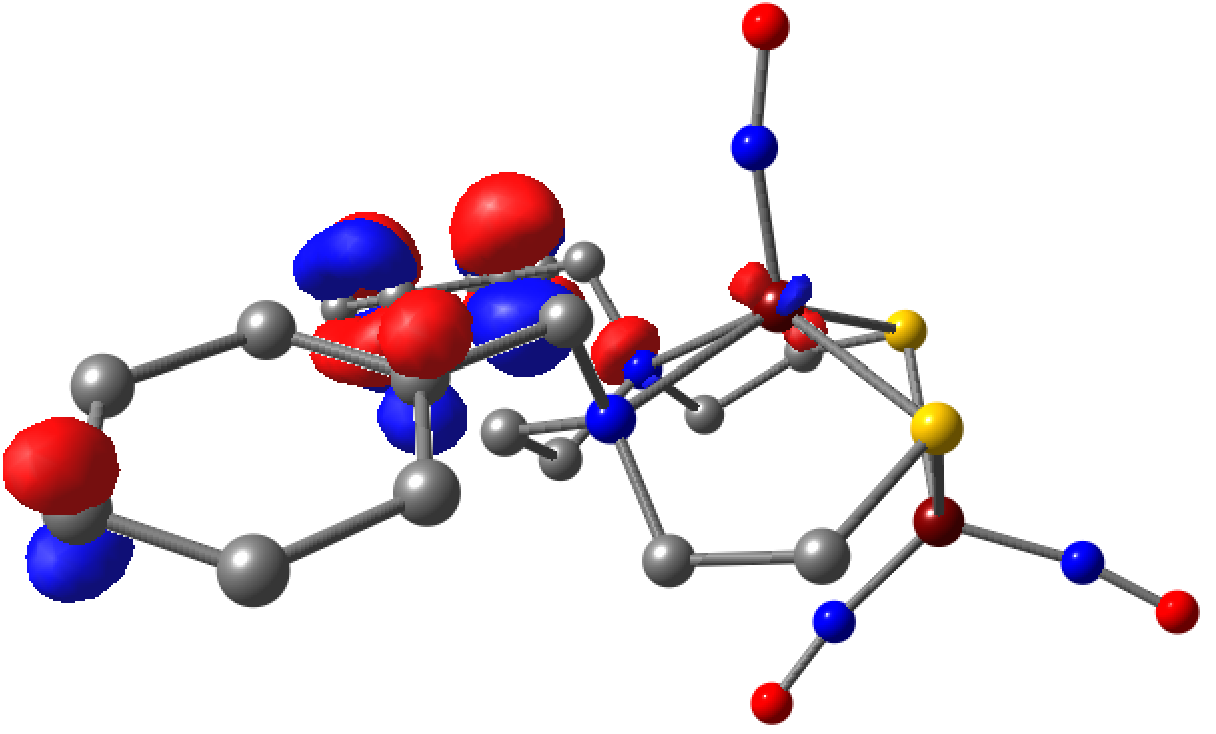  LUMO |
| 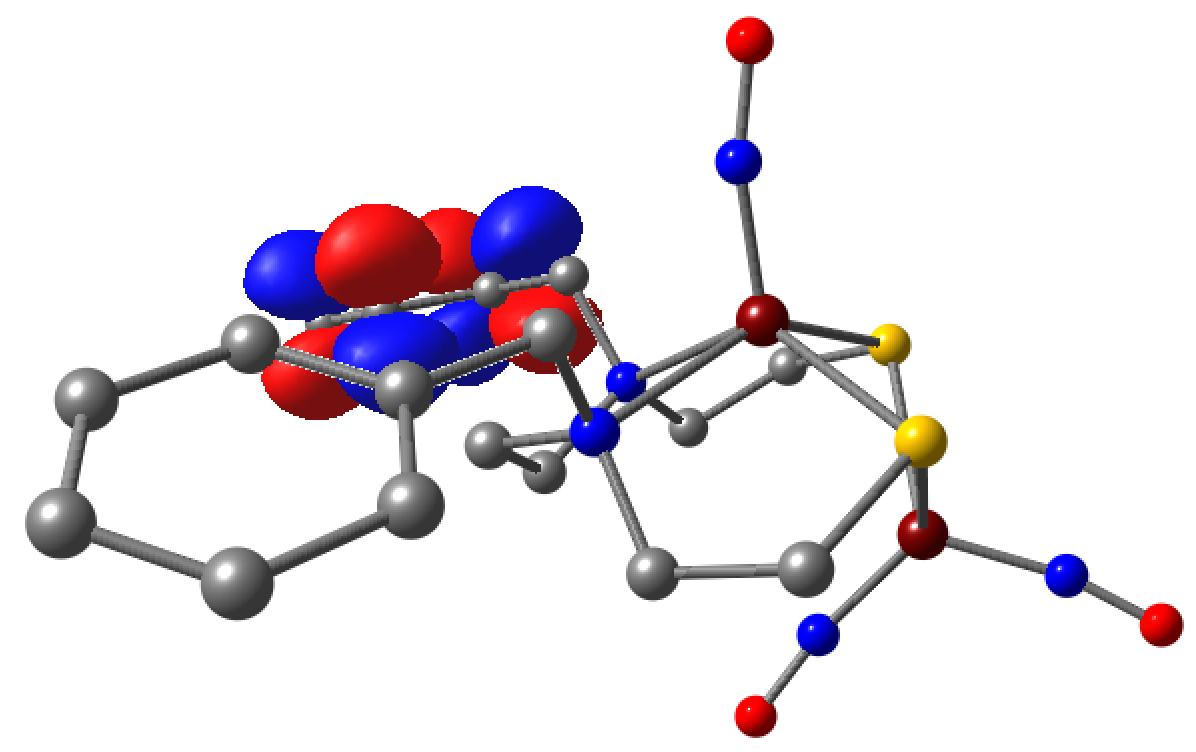  LUMO+1 | | 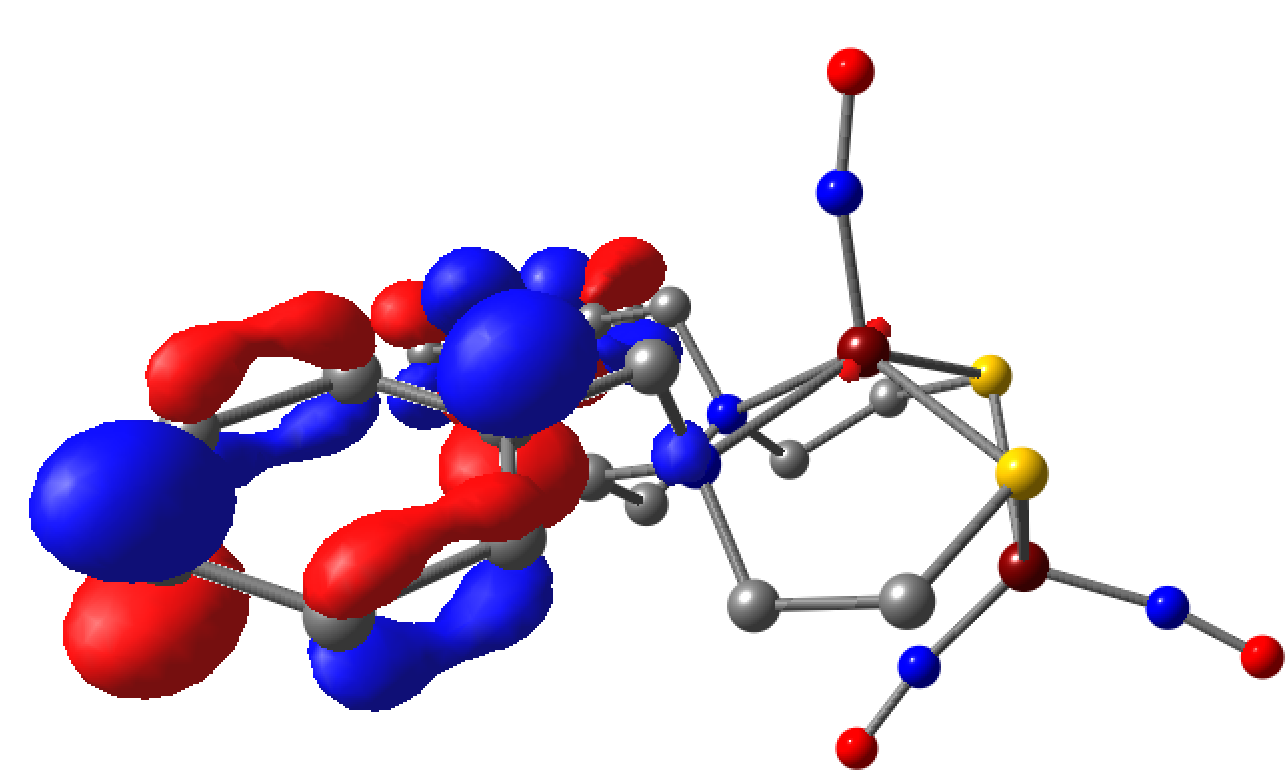  LUMO+1 |
| 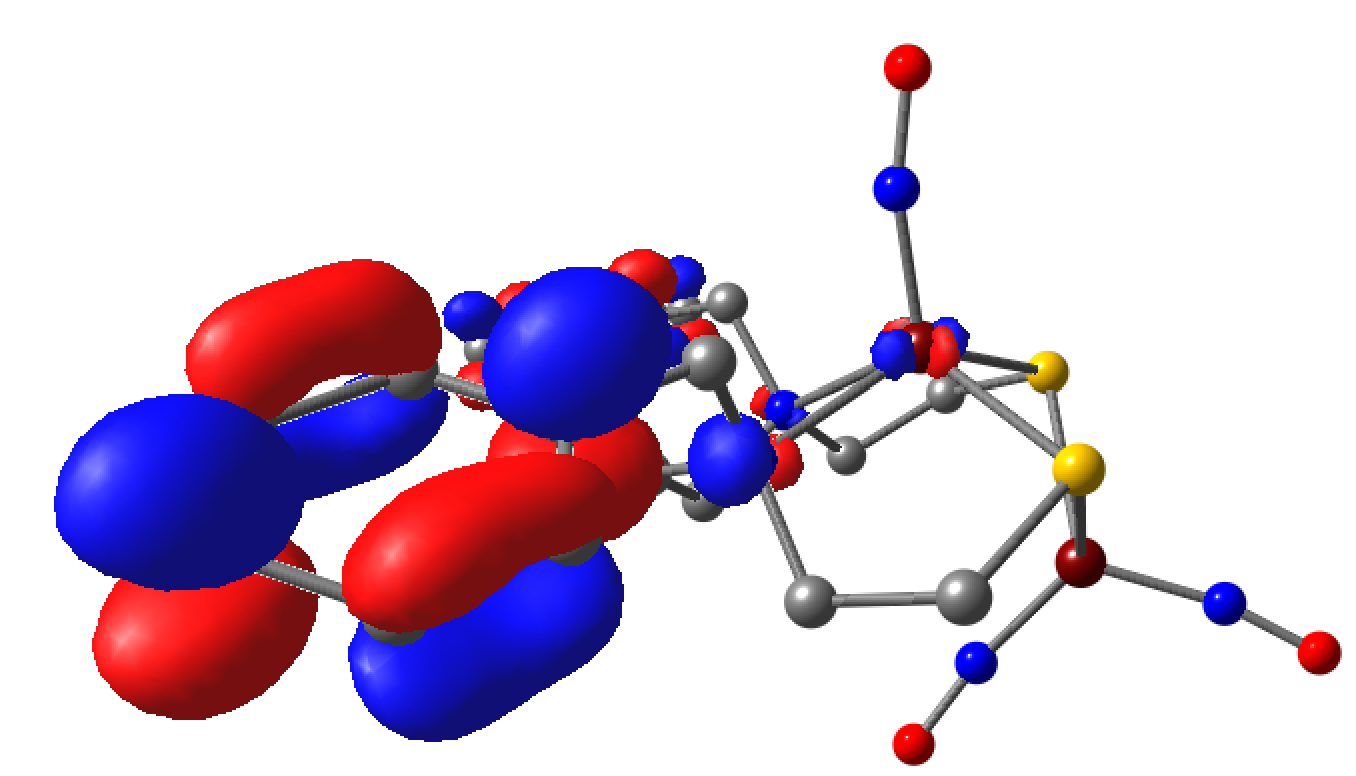  LUMO+2 | | 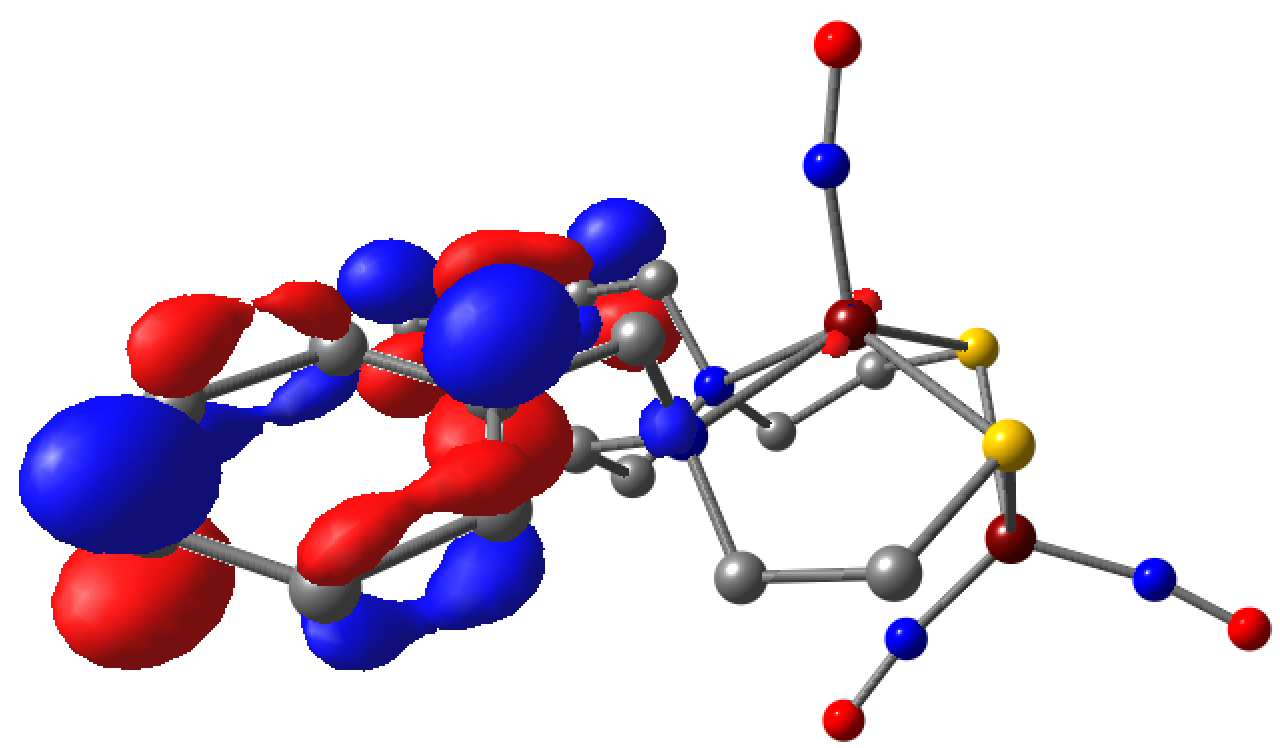  LUMO+2 |
| 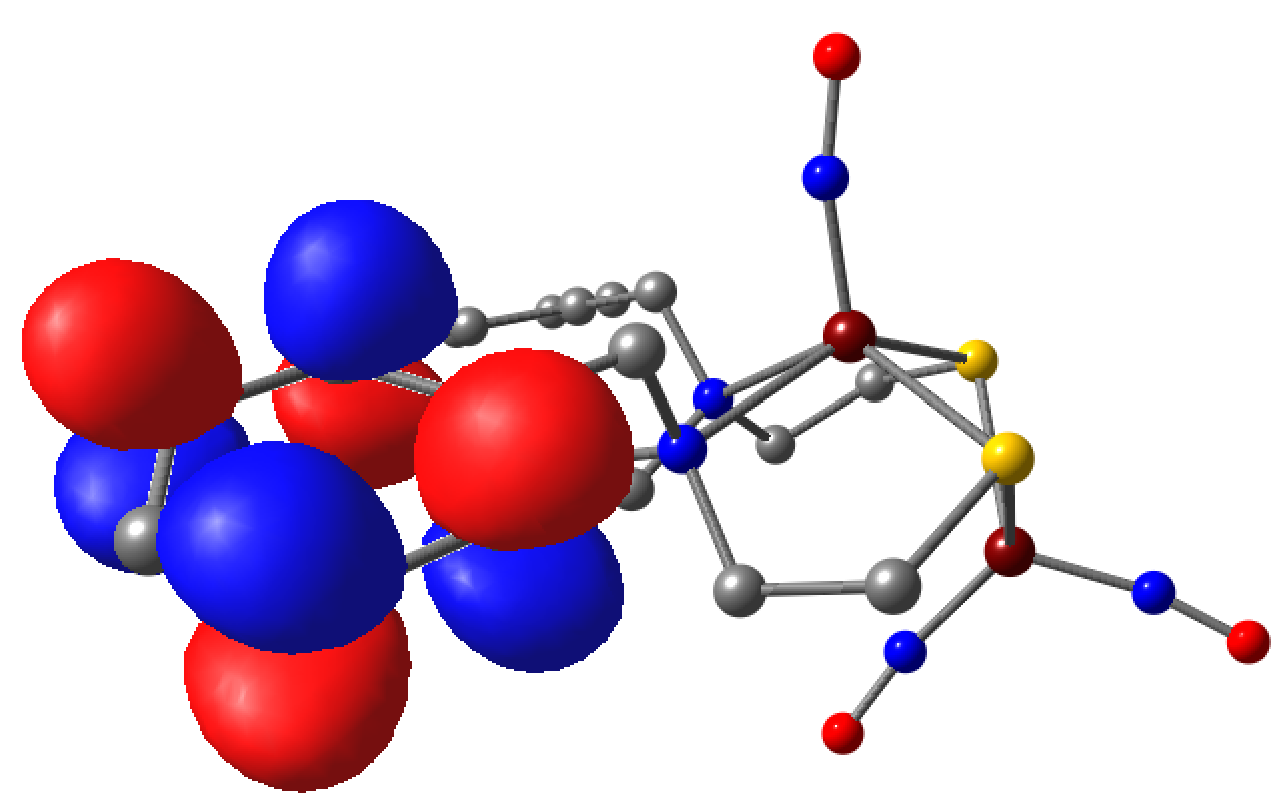  LUMO+3 | | 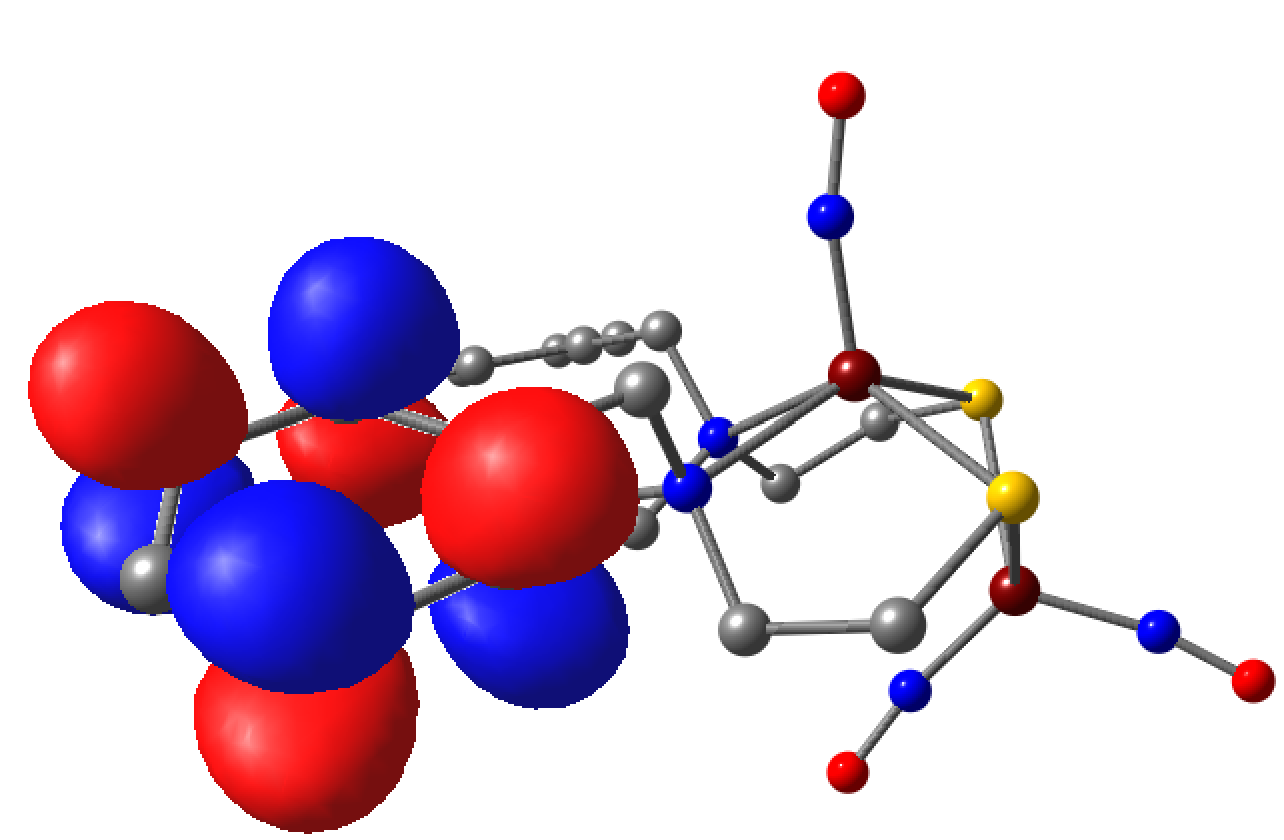  LUMO+3 |

**Table S14.** Alpha and Beta orbitals of **[^L3^Fe_2_(NO)_3_]^−^**, calculated at unrestricted TPSS/6-311++G(d,p) level of theory.

**Coordinates of the DFT optimized structure**

**[^L3^Fe_2_(NO)_3_]^+^ (BS singlet):**

| Fe 0.68072 -0.31518 0.83409  Fe 2.74726 -1.07987 -0.55749  S 2.77044 0.45726 1.08190  S 1.46104 -2.41054 0.69677  O 2.39680 -0.69026 -3.32257  O 0.19120 -0.14172 3.57647  N 0.24572 1.47862 -0.13907  N -1.05077 -1.04341 -0.10234  N 0.20781 -0.16357 2.40849  N 4.24045 -1.77160 -0.37115  N 2.36128 -0.76027 -2.14646  O 5.28413 -2.26530 -0.22484  C -0.61074 1.07281 -1.30727  H -1.05596 1.95493 -1.78641  H 0.05481 0.59250 -2.03053  C 0.17173 -3.18793 -0.39163  H 0.69574 -3.81344 -1.11936  H -0.42580 -3.83947 0.25238  C -0.64985 -2.11518 -1.08196  H -0.04819 -1.63226 -1.85649  H -1.55062 -2.53957 -1.54703  C -3.35897 -2.09658 0.38700  C -0.50844 2.40415 0.83217  H -1.34666 1.82052 1.22104  H 0.17907 2.57518 1.66460  C -2.25297 3.82554 -0.35018  H -2.90537 2.95499 -0.40533  C -0.99012 3.72048 0.26020  C -4.47264 -1.24003 0.32074 | H -4.39092 -0.21564 0.68199  C -2.04005 -1.59307 0.93648  H -2.21169 -0.76663 1.63219  H -1.51031 -2.37886 1.48105  C -1.89651 6.18601 -0.75955  H -2.24724 7.13742 -1.15068  C -1.68433 0.10702 -0.84131  H -2.27902 -0.25985 -1.68697  H -2.37412 0.59924 -0.15140  C 1.48279 2.16756 -0.65817  H 1.22745 3.18385 -0.98561  H 1.82421 1.59624 -1.52462  C -0.20123 4.87942 0.37423  H 0.75925 4.83268 0.88555  C -2.70228 5.04696 -0.86042  H -3.68355 5.11254 -1.32339  C -3.51759 -3.43439 -0.01788  H -2.68827 -4.13359 0.07363  C -5.70044 -1.69639 -0.16664  H -6.55334 -1.02336 -0.20099  C -0.64819 6.10242 -0.13389  H -0.02903 6.98977 -0.03092  C -4.74396 -3.89367 -0.50682  H -4.85082 -4.93260 -0.80784  C 2.57116 2.16839 0.39361  H 2.35731 2.81957 1.24726  H 3.53368 2.46699 -0.03248  C -5.83551 -3.02287 -0.58974  H -6.79131 -3.38136 -0.96278 |
| --- | --- |

**[^L3^Fe_2_(NO)_3_]^0^ (Doublet):**

| Fe 0.62357 -0.32513 0.88881  Fe 2.79464 -1.22273 -0.58763  S 2.75810 0.30287 1.14502  S 1.38961 -2.43418 0.76848  O 1.94821 -0.63715 -3.23704  O 0.35176 -0.11574 3.64002  N -1.12016 -1.01263 -0.06529  N 2.21501 -0.82213 -2.07923  N 0.28429 1.48860 -0.10592  N 0.09227 -0.10723 2.47663  N 4.27274 -1.95173 -0.55101  O 5.32715 -2.47431 -0.66936  C -2.12049 -1.54476 0.94939  H -2.26672 -0.73155 1.66627  H -1.61544 -2.35513 1.48083  C -0.70499 -2.08306 -1.03918  H -0.07362 -1.60201 -1.79002  H -1.59626 -2.49210 -1.53996  C -4.87718 -3.74067 -0.56922  H -5.00394 -4.76718 -0.90522  C -0.41295 2.45843 0.83954  H -1.27758 1.92802 1.24698  H 0.28147 2.61057 1.66965  C -3.45384 -2.00421 0.38763  C -3.64377 -3.32438 -0.05860  H -2.82631 -4.03946 0.01103  C -0.58302 1.09219 -1.26544  H 0.06667 0.57800 -1.97926  H -1.00312 1.97538 -1.76835 | C -1.69666 0.16540 -0.79579  H -2.31142 -0.16059 -1.64655  H -2.35880 0.68637 -0.09745  C -0.83364 3.79035 0.24682  C -2.09610 3.95002 -0.35135  H -2.78913 3.11031 -0.38062  C 1.57452 2.07576 -0.61955  H 1.88599 1.45160 -1.46028  H 1.40093 3.09903 -0.98249  C 0.01612 4.90853 0.31643  H 0.98326 4.81724 0.80809  C -0.37198 6.14097 -0.21771  H 0.29830 6.99466 -0.15046  C -5.94831 -2.84346 -0.63546  H -6.90908 -3.16736 -1.02837  C 0.09373 -3.17529 -0.34224  H 0.60502 -3.79700 -1.08281  H -0.53467 -3.82538 0.27655  C 2.65591 2.01998 0.44367  H 2.47000 2.70000 1.28309  H 3.62796 2.27119 0.00699  C -4.54705 -1.12142 0.33397  H -4.43778 -0.10756 0.71656  C -5.78248 -1.53297 -0.17520  H -6.61692 -0.83585 -0.20126  C -1.62335 6.27781 -0.82790  H -1.92764 7.23629 -1.24144  C -2.48841 5.18029 -0.88808  H -3.47051 5.28413 -1.34361 |
| --- | --- |

**[^L3^Fe_2_(NO)_3_]^−^ (Triplet):**

| Fe 0.57575 -0.46702 1.21933  Fe 2.52836 -1.83591 -0.58636  S 2.87645 -0.25329 1.12305  S 0.82973 -2.73704 0.74706  O -0.13900 0.15709 3.91508  O 1.72717 -1.06794 -3.21229  O 4.71515 -3.60991 -0.83540  N 0.59366 1.44591 -0.14434  N -1.40600 -0.75017 -0.06841  N -0.00435 -0.04908 2.72997  N 2.01210 -1.32069 -2.05801  N 3.80409 -2.87056 -0.59112  C 3.06342 1.40726 0.31523  H 3.07802 2.15452 1.12019  H 4.03121 1.44561 -0.19937  C 1.95812 1.66976 -0.70448  H 2.08198 0.95088 -1.51775  H 2.04522 2.68635 -1.12681  C -0.38144 1.20703 -1.23668  H 0.11392 0.55130 -1.95915  H -0.64242 2.13804 -1.77028  C -1.66936 0.54750 -0.73041  H -2.15530 1.19401 0.01053  H -2.37188 0.44423 -1.57685  C -1.14681 -1.83152 -1.05584  H -0.40789 -1.44915 -1.76394  H -2.06781 -2.06929 -1.62108  C -0.57682 -3.09634 -0.40988  H -0.21370 -3.76180 -1.20074 | H -1.33822 -3.64024 0.16546  C 0.18270 2.54143 0.79803  H -0.76805 2.23838 1.24670  H 0.91899 2.53916 1.60738  C 0.06031 3.93170 0.19462  C 1.15515 4.81560 0.18012  H 2.09608 4.50229 0.62935  C 1.04896 6.08934 -0.38731  H 1.90948 6.75587 -0.38509  C -0.16138 6.50954 -0.95134  H -0.24610 7.50061 -1.39266  C -1.26495 5.64922 -0.93269  H -2.21463 5.97126 -1.35618  C -1.15271 4.37666 -0.36276  H -2.02360 3.72317 -0.33574  C -2.47786 -1.10204 0.91503  H -2.47736 -0.30289 1.66412  H -2.14639 -2.01207 1.42494  C -3.87280 -1.28103 0.33583  C -4.77442 -0.20171 0.28493  H -4.47409 0.76100 0.69658  C -6.05199 -0.34787 -0.26458  H -6.73259 0.50154 -0.28691  C -6.45921 -1.58621 -0.77516  H -7.45309 -1.70383 -1.20245  C -5.58095 -2.67469 -0.71985  H -5.89200 -3.64555 -1.10142  C -4.30486 -2.52259 -0.16779  H -3.63692 -3.38026 -0.11402 |
| --- | --- |

**13. References**.

1. Golden, M.; Darensbourg, M. Y.; Irwin, J.; Frost, B. *N*,*N′*‐Bis(Mercaptoethyl)‐1,4‐Diazacycloheptane (H_2_BME‐DACH) and its Nickel Complex: A Model for Bioinorganic Chemistry. *Inorg. Synth.* **2014**, *36*, 231–240.
2. Smee, J. J.; Miller, M. L.; Grapperhaus, C. A.; Reibenspies, J. H.; Darensbourg, M. Y. Subtle Bite-Angle Influences on N_2_S_2_Ni Complexes. *Inorg. Chem.* **2001**, *40*, 3601–3605.
3. Mills, D. K.; Hsiao, Y. M.; Farmer, P. J.; Atnip, E. V.; Reibenspies, J. H.; Darensbourg, M. Y. Applications of the N_2_S_2_ Ligand, N,N’-Bis(mercapitoethyl)-l,5-diazacyclooctane, toward the Formation of Bi- and Heterometallics: [(BME-DACO)Fe]_2_ and [(BME-DACO)NiFeCl_2_]_2_. *J. Am. Chem. Soc.* **1991**, *113*, 1421–1423.
4. Karlin, K. D.; Lippard, S. J. Sulfur-Bridged Binuclear Iron(II) Complexes. Effect of Ligand Constraints on Their Physical Properties; Reactions with Carbon Monoxide and Alkyl Isocyanides *J. Am. Chem. Soc.* **1976**, *98*, 6951–6957.
5. Mills, D. K.; Font, I.; Farmer, P. J.; Hsiao, Y.-M.; Tuntulani, T.; Buonomo, R. M.; Goodman, D. C.; Musie, G.; Grapperhaus, C. A.; Maguire, M. J.; Lai, C.-H.; Hatley, M. L.; Smee, J. J.; Bellefeuille, J. A.; Darensbourg, M. Y.; Hancock, R. D.; Eng, S.; Martell, A. E. Inorganic Syntheses, John Wiley & Sons, Ltd, 1998, pp. 89–98.
6. Chiang, C. -Y.; Lee, J.; Dalrymple, C.; Sarahan, M. C.; Reibenspies, J. H.; Darensbourg, M. Y. Synthesis and Molecular Structures of Mononitrosyl (N_2_S_2_)M(NO) Complexes (M = Fe, Co). *Inorg. Chem.* **2005**, *44*, 9007–9016.
7. Karlin, K. D.; Rabinowitz, H. N.; Lewis, D. L.; Lippard, S. J.  Synthesis and Characterization of the Pentacoordinate Mononitrosyliron Complexes Fe (NO)[SCH_2_CH_2_N(CH_3_)(CH_2_)_n_N(CH_3_)CH_2_CH_2_S], n= 2, 3. *Inorg. Chem.* **1977**, *16*, 3262–3267.
8. Hsieh, C. -H.; Darensbourg, M. Y. A {Fe(NO)_3_}^10^ Trinitrosyliron Complex Stabilized by an N-Heterocyclic Carbene and the Cationic and Neutral {Fe(NO)_2_}^9/10^ Products of Its NO Release. *J. Am. Chem. Soc.* **2010**, *132*, 14118–14125.
9. Ghosh, P.; Ding, S.; Quiroz, M.; Bhuvanesh, N.; Hsieh, C. -H.; Palacios, P. M.; Pierce, B. S.; Darensbourg, M. Y.; Hall, M. B. Structural and Electronic Responses to the Three Redox Levels of Fe(NO)N_2_S_2_‐Fe(NO)_2_. *Chem. Eur. J.***2018**, *24*, 16003–16008.
10. SAINT (Version 7.34A); Bruker AXS Inc.: Madison, WI, USA, 2007.
11. Sheldrick, G. M. Crystal Structure Sefinement with SHELXL. *Acta Crystallogr., Sect. A* **2008**, *64*, 112–122.
12. Dolomanov, O. V.; Bourhis, L. J.; Gildea, R. J.; Howard, J. A. K.; Puschmann, H. *OLEX2*: A Complete Structure Solution, Refinement and Analysis Program. *J. Appl. Cryst.* **2009**, *42*, 339–341.
13. Connelly, N. G.; Draggett, P. T.; Green, M.; Kuc, T. A. Synthesis and Reactivity of the Tetrakis(acetonitrile)nitrosylrhodium Dication. *J. Chem. Soc, Dalton Trans* **1977**, *0*, 70–73.
14. Denny, J. A.; Darensbourg, M. Y. Metallodithiolates as Ligands in Coordination, Bioinorganic, and Organometallic Chemistry. *Chem. Rev.* **2015**, *115*, 5248–5273.
15. Denny, J. A.; Darensbourg, M. Y. CCDC 1046569: Experimental Crystal Structure Determination, 2016, DOI: 10.5517/ccdc.csd.cc14419m.
16. Hu, W.-J.; Lippard, S. J. Effect of Ligand Constraints on the Geometry of Two Sulfur-Bridged Binuclear Iron(II) Complexes. Structures of Bis {μ-[N,N’-Dimethyl-N,N’-Bis(β-Mercaptoethyl)Ethylenediamine]}-Diiron(II) and Bis{μ-[N,N’-Dimethyl-N,N’-Bis(β-Mercaptoethyl)-1,3-Propanediamine]}-Diiron(II). *J. Am. Chem. Soc.* **1974**, *96*, 2366–2372.
17. Brothers, S. M.; Darensbourg, M. Y.; Hall. M. B. Modeling Structures and Vibrational Frequencies for Dinitrosyl Iron Complexes (DNICs) with Density Functional Theory. *Inorg. Chem.* **2011**, *50*, 8532–8540.
18. Bain, G. A.; Berry, J. F. Diamagnetic Corrections and Pascal’s Constants. *J. Chem. Educ.* **2008**, *85*, 532–536.
